# Tunable electrochemiluminescence of TADF luminophores: manipulating efficiency and unveiling water-soluble emitters†

**DOI:** 10.1039/d4sc04986a

**Published:** 2024-10-03

**Authors:** Alessandro Fracassa, Francesco Calogero, Giulio Pavan, Pavlos Nikolaou, Andrea Fermi, Paola Ceroni, Francesco Paolucci, Pier Giorgio Cozzi, Thomas Scattolin, Nicola Demitri, Fabrizia Negri, Andrea Gualandi, Alessandro Aliprandi, Giovanni Valenti

**Affiliations:** a Dipartimento di Chimica “Giacomo Ciamician”, Alma Mater Studiorum – Università di Bologna Via Gobetti 85 40129 Bologna Italy g.valenti@unibo.it andrea.gualandi10@unibo.it; b Center for Chemical Catalysis – C3, Alma Mater Studiorum – Università di Bologna Via Gobetti 85 40129 Bologna Italy; c Dipartimento di Scienze Chimiche, Università Degli Studi di Padova Via Marzolo 1 35131 Padova Italy alessandro.aliprandi@unipd.it; d Elettra-Sincrotrone Trieste S.S 14 Km 163.5 in Area Science Park 34149 Basovizza – Trieste Italy

## Abstract

Thermally Activated Delayed Fluorescent (TADF) luminophores offer the potential to achieve 100% Internal Quantum Efficiency (IQE) by harvesting both singlet and triplet excitons *via* reverse intersystem crossing from T_1_ to S_1_. This class of molecules has therefore been embraced in the pursuit of cheaper and more efficient electrochemiluminescent (ECL) labels. The present study explores how tuning the electron-donating (D) and -accepting (A) strengths of peripheral substituents affects the ECL emission of mono- and dicyanoarene-based TADF dyes. To this end, we synthesized two series of TADF compounds, independently manipulating electron donors and acceptors by (i) halogenating electron-rich diphenylamine moieties, or (ii) mono- or di-substituting the electron-poor cyanoarene core with either fluorine or imidazole. Through a comparative analysis, we elucidate the role of each substituent in shaping the photophysics of the investigated luminophores. Despite only achieving a relative *Φ*_ECL_ as high as 1.27%, this framework identifies several molecular features that boost the ECL efficiency to pave the way for designing highly efficient TADF-based ECL emitters. Ultimately, imidazole substituents are exploited as a platform for functionalization with triethylene glycol units. The resulting water-soluble TADF luminophores are characterized under conditions usual to commercial ECL bioanalysis, proving their potential as a cost-effective alternative replacement to [Ru(bpy)_3_]^2+^ in clinical diagnostic.

## Abstract 2

The ECL efficiency of several TADF dyes was evaluated by tuning the electron-donating and -accepting strengths of peripheral substituents. Introducing TEG chains provides water-solubility and expresses TADF potential as ECL labels for bioanalysis.

## Graphical abstract

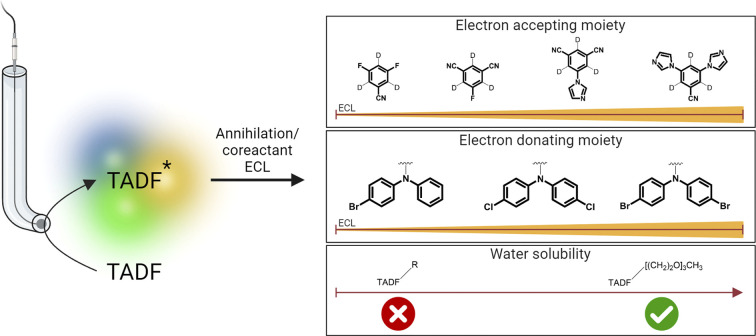

## Introduction

Electrochemiluminescence (ECL) is the process of emitting photons from an excited luminophore through an electrochemically driven process. As the name suggests, the ECL process is initiated by a heterogeneous electrochemical reaction, which in turn triggers a homogeneous electron transfer process. This sequence results in the excitation of a given luminophore, ultimately leading to its emission. This technique finds applications across a variety of fields, from clinical diagnosis and bioanalytical chemistry to the imaging of micro- and nano-sized objects, thanks to its superior sensitivity and well-resolved spatial resolution.^1–4^

Since the discovery of ECL, extensive research efforts have been devoted to the development of novel ECL luminophores exhibiting ever-increasing emission intensities.^5^ However, the implementation of efficient dyes remains limited to a few candidates, basically because of the underlying spin statistics that yield 75% of triplet excitons upon charge recombination. Due to this constraint, molecular design has traditionally focused on metal complexes, the only class of molecules capable of efficient emission directly from the lowest excited triplet state (T_1_).

Despite its poor photoluminescence quantum yield (PLQY, *Φ*_PL_ = 0.095),^6^ tris(2,2′-bipyridine)ruthenium(ii) ([Ru(bpy)_3_]^2+^) is still the most widely employed label since its very first application in the ECL field, primarily thanks to its superior ECL efficiency and its well-known bioconjugation chemistry.^7,8^ Over the last decade, cyclometalated Ir(iii) complexes have been drawing significant attention as alternative ECL luminophores to the conventional Ru(ii) polypyridine chelates due to their intrinsically greater PLQY and finely tunable properties.^9–12^ Characteristics such as solubility, emission wavelengths, and electrochemical potentials can be controlled by manipulating the ligands structure, paving the way to design novel approaches for ECL applications such as multi-color and/or potential-resolved ECL systems or redox-mediated enhanced ECL.^13–17^

While Ir(iii) complexes intrinsically address common problems associated with Ru(ii) complexes, such as poor emission tunability and modest PLQY, both of these metals are among the scarcest ones, and the consequent cost hampers large-scale production and commercial viability. Moreover, the biocompatibility of metal-containing molecules is not guaranteed, further limiting their range of applicability.

On the other hand, organic molecules offer many advantages compared to metal complexes, such as tailorable synthesis, inexpensive starting materials, tunable molecular properties, and biocompatibility (*i.e.*, metal-free molecular structure). Thus, they would be ideal candidates to replace coordination complexes as convenient and scalable ECL luminophores. Unfortunately, the radiative transition from T_1_ to the ground state (S_0_) is spin-forbidden for most organic luminophores. Therefore, the ECL efficiency of organic fluorophores is restricted to 25%, corresponding to the population of the lowest excited singlet state (S_1_) upon charge recombination.

To overcome the limited efficiency, cyanoarene derivatives manifesting Thermally Activated Delayed Fluorescence (TADF) have proven to be effective.^18–20^ Electron-accepting (A) cyanoarenes surrounded by electron-donating (D) groups display spatially separated highest occupied molecular orbital (HOMO) and lowest unoccupied molecular orbital (LUMO), resulting in a very small energy gap (∼0.1 eV) between S_1_ and T_1_ (Δ*E*_ST_). The emissive S_1_ state is then populated from the T_1_ state *via* reverse intersystem crossing, and delayed fluorescence is observed in addition to prompt fluorescence. As a result, both singlet and triplet excitons can be effectively harvested, enabling a theoretical internal quantum efficiency (IQE) of 100%.

Additionally, parallel to Ir(iii) complexes, TADF emitters offer a versatile scaffold for tailoring optoelectronic properties by judiciously altering the extent of electron-donating and/or electron-accepting effect of the peripheral groups.

In the present work, we propose a comprehensive investigation on two series of cyanoarene derivatives. The electronic properties of the synthesized luminophores were finely tuned through (i) halogenation of D diphenylamine (DPA) moieties, or (ii) functionalization of the A cyanoarene core with either fluorine or imidazole. Following a comparative approach, we correlate the photophysical, electrochemical, ECL, and DFT data of the whole set of emitters to elucidate the impact of molecular design on their ECL behavior in aprotic conditions.

Eventually, we demonstrated the versatility of the imidazole heterocycle as a conjugation platform for water-soluble triethylene glycol (TEG) chains by reporting the coreactant ECL in an aqueous solution in the case of TEG-derivatized organic dyes, without requiring any encapsulation in a water-soluble matrix.^21,22^

## Results and discussion

**Fig. 1:**
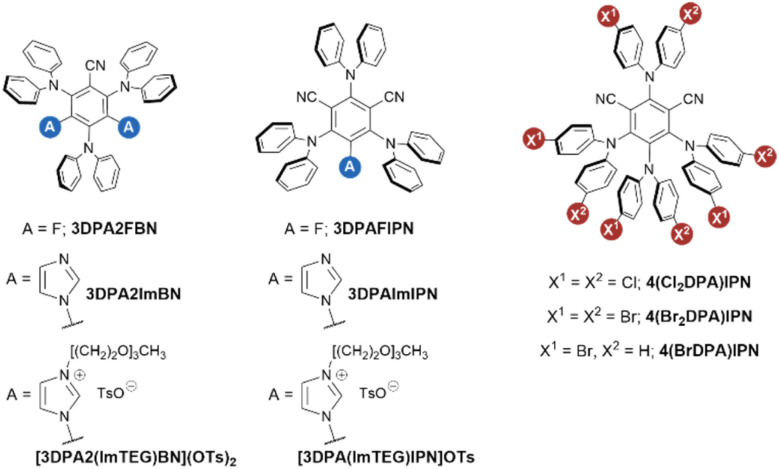
Molecular structures of three different scaffolds, namely 3DPABN, 3DPAIPN, and 4DPAIPN. The first two molecular platforms underwent further modification by derivatizing the cyanoarene accepting core with fluorine atoms (*i.e.*, 3DPA2FBN and 3DPAFIPN), imidazole heterocycles (*i.e.*, 3DPA2ImBN and 3DPAImIPN), or imidazole units bearing a triethylene glycol chain to provide water solubility (*i.e.*, [3DPA2(ImTEG)BN](OTs)_2_ and [3DPA(ImTEG)IPN]OTs). On the other hand, the functionalization of 4DPAIPN focused on derivatizing the electron-donating DPA groups, which were subjected to mono- or bi-insertion of bromine or chlorine atoms (*i.e.*, 4(BrDPA)IPN, 4(Br_2_DPA)IPN and 4(Cl_2_DPA)IPN).

To exhaustively analyze the influence of molecular architecture on the ECL emission of TADF dyes, we designed a set of molecules to exhibit progressively increasing A strength and gradually decreasing D strength. The impact of these two effects is separately addressed by synthesizing two series of organic emitters by independently manipulating the donating properties of the electron-rich periphery and the accepting properties of the electron-poor cyanoarene-based core (Fig. 1).

In particular, we chose DPA as electron-rich substituent, as the ECL performance of DPA-based TADF luminophores has been scarcely investigated in the literature, especially in comparison to their carbazole-based counterparts.^19,23^ The electron-donating strength of DPA units was progressively weakened through the insertion of one or two halogen atoms such as Br and Cl in 4 and 4′ position: 4-bromo-*N*-phenylaniline (4BrDPA), bis(4-chlorophenyl)amine (4Cl_2_DPA), and bis(4-bromophenyl)amine (4Br_2_DPA). On the other hand, we functionalized electron-poor benzonitrile and isophtalonitrile cores with either imidazole or fluorine, resulting in a set of four different compounds with gradually increasing electron-withdrawing strength: 3,5-difluoro benzonitrile (2FBN), 3,5-diimidazol-1-yl benzonitrile (2ImBN), 5-fluoro isophthalonitrile (FIPN), and 5-imidazole-1-yl isophthalonitrile (ImIPN). Finally, we evaluate the ECL behavior of TEG-derivatives of 3DPAImIPN and 3DPA2ImBN, namely [3DPA(ImTEG)IPN]OTs and [3DPA2(ImTEG)BN](OTs)_2_, under chemical conditions mimicking those employed in commercial ECL-based assays.

### Tuning electron-accepting strength of the cyanoarene ring

The summarized photophysical, electrochemical, and ECL data for acceptor-modified molecules are reported in Table 1.

**Table 1:** Photophysical, electrochemical and ECL data for acceptor-modified molecules in acetonitrile solution

	3DPAFIPN	3DPA2FBN	3DPAImIPN	3DPA2ImBN
*λ*_abs_ a (nm)	—	—	361	341
*λ*_em_ b (nm)	525 (ref. 24)	491 (ref. 24)	545	541
*τ*_prompt_ c (ns)	4.2 (ref. 24)	4.2 (ref. 24)	5.18	7.01
*τ*_prompt_ d (ns)	—	—	5.65	8.25
*τ*_delayed_ c (μs)	—	—	0.83	0.247
*τ*_delayed_ d (μs)	—	—	49.6	26.4
*Φ*_PL_ e (%)	—	—	11.3	6.6
*Φ*_PL_ f (%)	—	—	32.5	35.7
g *E*_peak_^ox^ (V)	1.24	1.25	1.35	1.35
g *E*_1/2_^red^ (V)	−1.59	−1.85	−1.49	−1.68
*Φ*_ECL_ h	0.0043	0.0016	0.0082	0.01
*λ*_ECL_ i (nm)	526	482	534	514

a Air-equilibrated acetonitrile solutions (5 × 10^−6^ M).

b Ar-purged acetonitrile solutions (5 × 10^−6^ M), *λ*_exc_ = 340–361 nm.

c Lifetimes were evaluated on air-equilibrated acetonitrile solutions, *λ*_exc_ = 402.3 nm.

d Lifetimes were evaluated on Ar-purged acetonitrile solutions, *λ*_exc_ = 402.3 nm.

e Air-equilibrated solution.

f Ar-purged solution.

g 1 × 10^−3^ M solution in degassed AcN/TBAPF_6_ 0.1 M. Working glassy carbon electrode (1 mm); quasi-reference Ag wire electrode; counter Pt wire. Scan rate: 100 mV s^−1^.

h 0.5 × 10^−3^ M solution in degassed AcN/TBAPF_6_ 0.1 M. *Φ*_ECL_ determined under annihilation conditions according to eqn (S1); [Ru(bpy)_3_](PF_6_)_2_ as standard (*Φ*_ECL_ = 0.05). The ECL signal is normalized over the quantum efficiency of the spectral response of the PMT at the wavelength of maximum ECL emission.

i 0.5 × 10^−3^ M solution in degassed AcN/TBAPF_6_ 0.1 M/BPO 10 mM.

Absorption, emission, and excitation spectra are presented in Fig. S1–S4.† The results of DFT calculations are reported in Table S1 and Fig. S5–S8.† The XRD structure of 3DPA2ImBN is shown in Fig. S9.†

**Fig. 2:**
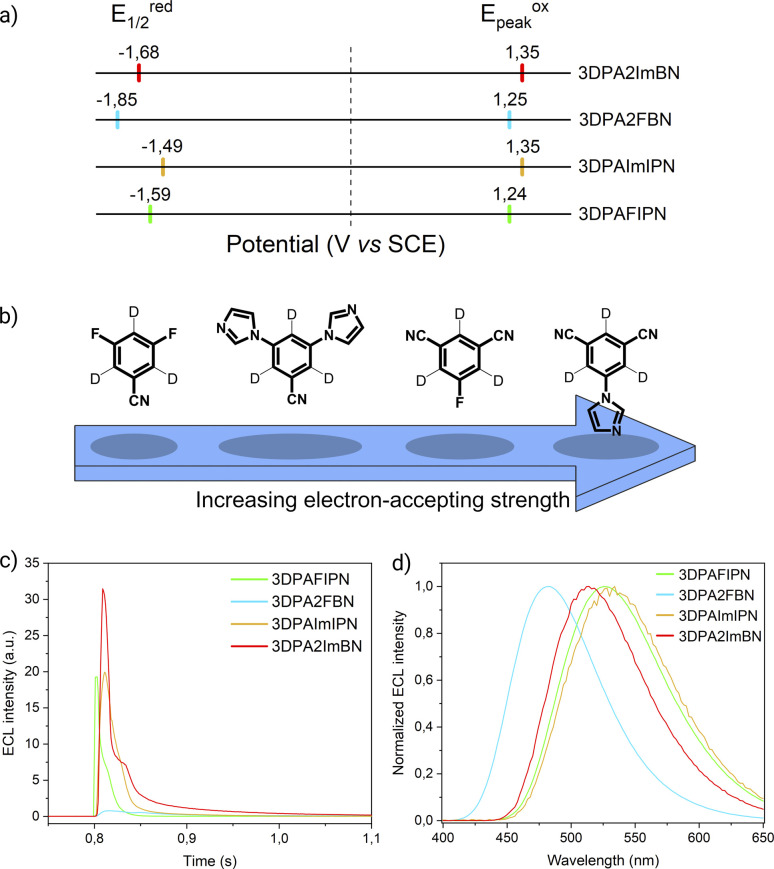
(a) Scheme showing the half-wave reduction potentials (*E*_1/2_^red^, left) and oxidation peak potentials (*E*_peak_^ox^, right) of the acceptor-modified TADF luminophores. The dotted line marks the potential of 0 V. (b) Scheme representing the electron-accepting strength of fluorine- and imidazole-modified cyanoarene cores as determined by their reduction potentials. (c) First peak ECL intensity of 3DPAFIPN (green line), 3DPA2FBN (blue line), 3DPAImIPN (yellow line), and 3DPA2ImBN (red line) (0.5 mM) in degassed AcN/TBAPF_6_ 0.1 M collected *vs.* time. PMT bias of 750 V; 000.0 μA amplification. (d) ECL spectra of 3DPAFIPN, 3DPA2FBN, 3DPAImIPN, and 3DPA2ImBN (same color code as in Fig. 2c) (0.5 mM) in degassed AcN/TBAPF_6_ 0.1 M/BPO 10 mM. PMT bias of 700 V; integration time 100 ms; step 2 nm.

Cyclic voltammograms and redox potentials of 3DPAFIPN, 3DPAImIPN, 3DPA2FBN, and 3DPA2ImBN are presented in Fig. S10† and 2a, respectively. For each molecule, both oxidation peaks are associated with the chemically irreversible one-electron oxidation of a DPA unit to its N-centered radical cation. The high spin density at the *para* position destabilizes the DPA radical cation, which undergoes a chemical dimerization to a bridging *N*,*N*′-biphenylbenzidine (BPB) moiety *via* irreversible oxidative coupling between two radical cations.^25^ On the other end of the potential scale, the reversible one-electron reduction is attributed to the formation of a radical anion delocalized all over the cyanoarene-accepting core as supported by the shape of the computed LUMOs (Fig. S5–S8†). By modifying the cyanoarene core, we successfully manipulated the reduction potential, resulting in isophtalonitrile-based molecules being less reducing (*E*_1/2_^red^(3DPAFIPN) = −1.59 V, *E*_1/2_^red^(3DPAImIPN) = −1.49 V) than their benzonitrile-based counterparts (*E*_1/2_^red^(3DPA2FBN) = −1.85 V, *E*_1/2_^red^(3DPA2ImBN) = −1.68 V). This variation is dictated by the electron-withdrawing strength displayed by each core (Fig. 2b). Benzonitrile derivative emerges as a generally weaker electron-acceptor compared to isophtalonitrile counterpart. As suggested by the Hammett constants, this is due to the replacement of a strongly accepting cyano group (*σ*_m_ = +0.678) with a fluorine atom (*σ*_m_ = +0.337) or an imidazole ring,^26^ both of which manifest comparatively weaker accepting properties, resulting in an overall weaker accepting core.

In particular, the fluorine-containing molecules consistently expresses a greater reduction potential due to its dual character of σ-acceptor and π-donor: although being an overall electron-accepting substituent, the strong inductive effect of fluorine is partially counterbalanced by its positive mesomeric effect, which pushes electron density towards the core.^24^ Despite providing a weaker inductive effect than fluorine, the imidazole ring emerges as an overall stronger electron-withdrawing substituent. This is attributed to the limited π-donor character due to the internal conjugation of the ring that restricts the delocalization of the lone pair on the pyrrole-like nitrogen. These trends are fully supported by computed frontier orbital energies (Table S1†).

By virtue of their TADF feature, all the investigated molecules result in ECL emission upon cycling the potential between the first reduction and the first oxidation within diffusion-controlled potential regions (Fig. 2c and S11–S14†). This approach induces the so-called “annihilation ECL”, governed by the mechanism displayed in eqn (1)–(4):

1 TADF + e^−^ → TADF˙^−^

2 TADF − e^−^ → TADF˙^+^

3 TADF˙^+^ + TADF˙^−^ → TADF* + TADF

4 TADF* → *hν* + TADF

Leveraging the reversibility of the reduction process centered on the cyanoarene core, we initially applied a cathodic step followed by the anodic one. Annihilation served as a convenient method for determining *Φ*_ECL_. Although the scientific community is transitioning its effort from determining a relative ECL quantum yield to an absolute one, our goal is limited to investigate how variations in the donor–acceptor (D–A) architecture impact the ECL emission of TADF emitters.^27^ Fluorine-substituted emitters display a notably low *Φ*_ECL_, with a decrease from 3DPAFIPN to 3DPA2FBN. This behavior is potentially linked to the replacement of a cyano group with a fluorine atom. Dicyanobenzene derivatives are acknowledged for enhancing the emission of TADF compounds by mitigating both non-radiative decay and geometric alterations between the ground and the excited state molecule.^18,28^ Consequently, it seems plausible that fluorine may not provide the same positive effect, as indicated by the drop in ECL emission quantum efficiency upon substituting the second cyano moiety. On the other hand, the imidazole-substituted dyes exhibit a generally higher *Φ*_ECL_, reflecting a ∼0.03 eV decrease in Δ*E*_ST_ relatively to fluorine-substituted counterparts (Table S1†). Interestingly, the *Φ*_ECL_ of 3DPA2ImBN surpasses that of 3DPAImIPN, defying the previously observed trend with fluorine-based emitters. This reversal suggests the imidazole ring apparently plays an active role in improving the TADF behavior of this class of luminophores. Cyclic voltammograms don't manifest the reduction of the heterocycle in the investigated potential region as previously observed by Pavan *et al.*^29^ Furthermore, the XRD structure of 3DPA2ImBN (Fig. S9†) reveals an orthogonal orientation between the heterocycles and the cyanoarene core, implying poor orbital overlap. On the basis of the experimental observations, the imidazole ring seems an ancillary substituent, likely not contributing significantly to the LUMO. This hypothesis aligns with the lack of electron density on imidazole moieties in computed orbitals (Fig. S5–S6†). This phenomenon directs the electronic distribution of the LUMO onto the cyanoarene core, enhancing the charge transfer (CT) character of the excited state. As a result, the Δ*E*_ST_ between S_1_ and T_1_ states sharing the same orbital nature orbitals decreases from 3DPAImIPN to 3DPA2ImBN. The reduced delayed fluorescence lifetime (*τ*_delayed_) of 3DPA2ImBN compared to 3DPAImIPN further support the latter statement as delayed fluorescence inherently reflects the spin-flip rate from T_1_ to S_1_. Additionally, the ECL of the investigated luminophores was assessed with the aid of a coreactant, namely benzoyl peroxide (BPO). Coreactant ECL emission was recorded during a cathodic potential sweep (*i.e.*, CV-ECL), providing insights into ECL behavior under steady radical generation rather than alternating production. Consistently with many other coreactant ECL systems, the light emission is typically observed around the reduction potential of the most reducing species, namely the luminophore, while the reduction of BPO occurs at lower cathodic potentials (Fig. S15–S18†). As displayed in eqn (5)–(8), upon applying a suitable cathodic potential, the radical anions of both species are simultaneously generated. The reduced coreactant rapidly decomposes into a benzoate anion (PhCO_2_^−^) and a strongly oxidizing benzoate radical 
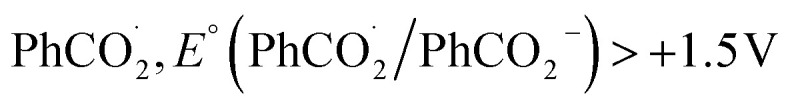
*vs.* SCE)^30^ that oxidizes the reduced luminophore, yielding its emitting excited state.

5 TADF + e^−^ → TADF˙^−^

6
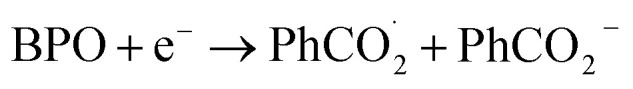
7
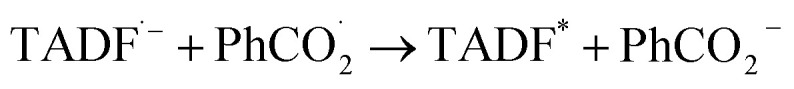

8 TADF* → *hν* + TADF

Coreactant ECL offers a more stable and enduring emission that is crucial for recording well-defined ECL spectra obtained by applying a suitable voltage in the diffusion-limited region. Although the ECL peak wavelength of some compounds appears blue-shifted compared to PL, our group has consistently observed this phenomenon in prior studies.^31–33^ We attribute this discrepancy to experimental factors, such as variations in slit width, different instrumentation, and sample concentration between PL and ECL measurements.

### Tuning electron-donating strength of DPA substituents

The summarized photophysical, electrochemical, and ECL data for donor-modified molecules are reported in Table 2.

**Table 2:** Photophysical, electrochemical and ECL data for donor-modified molecules in acetonitrile solution

	4(BrDPA)IPN	4(Cl_2_DPA)IPN	4(Br_2_DPA)IPN
*λ*_abs_ a (nm)	471	470	471
*λ*_em_ b (nm)	532	531	533
*τ*_prompt_ c (ns)	1.4	2.3	1.3
*τ*_delayed_ c (μs)	0.96	1.5	1.8
*τ*_delayed_ d (μs)	20	47	12
*Φ*_PL_ e (%)	3.6	5.0	2.6
*Φ*_PL_ f (%)	15	37	21
g *E*_1/2_^ox^ (V)	1.14	1.21	1.24
g *E*_1/2_^red^ (V)	−1.52	−1.45	−1.41
*Φ*_ECL_ h	0.0011	0.0098	0.0127
*λ*_ECL_ i (nm)	532	530	528

a Air-equilibrated solution in acetonitrile (10^−5^ M).

b Ar-purged solution in acetonitrile (10^−5^ M), *λ*_exc_ = 405 nm.

c Lifetimes were evaluated on air-equilibrated solutions, *λ*_exc_ = 405 nm.

d Lifetimes were evaluated on Ar-purged solutions, *λ*_exc_ = 405 nm.

e Air-equilibrated solution.

f Ar-purged solution.

g 1 × 10^−3^ M Ar-purged solution in AcN/TBAPF_6_ 0.1 M. Working glassy carbon electrode (1 mm); quasi-reference Ag wire electrode; counter Pt wire. Scan rate: 100 mV s^−1^.

h 0.5 × 10^−3^ M Ar-purged solution in AcN/TBAPF_6_ 0.1 M. *Φ*_ECL_ determined under annihilation conditions according to eqn (1); [Ru(bpy)_3_](PF_6_)_2_ as standard (*Φ*_ECL_ = 0.05). The ECL signal is normalized over the quantum efficiency of spectral response of the PMT at the wavelength of maximum ECL emission.

i 0.5 × 10^−3^ M Ar-purged solution in AcN/TBAPF_6_ 0.1 M/BPO 10 mM.

Absorption, emission spectra, and luminescence decay curves are presented in Fig. S19–S27.† The results of DFT calculations are reported in Table S3 and Fig. S28–S31.†

Cyclic voltammograms and redox potentials of 4(BrDPA)IPN, 4(Br_2_DPA)IPN, and 4(Cl2DPA)IPN are presented in Fig. S32† and 3a, respectively.

**Fig. 3:**
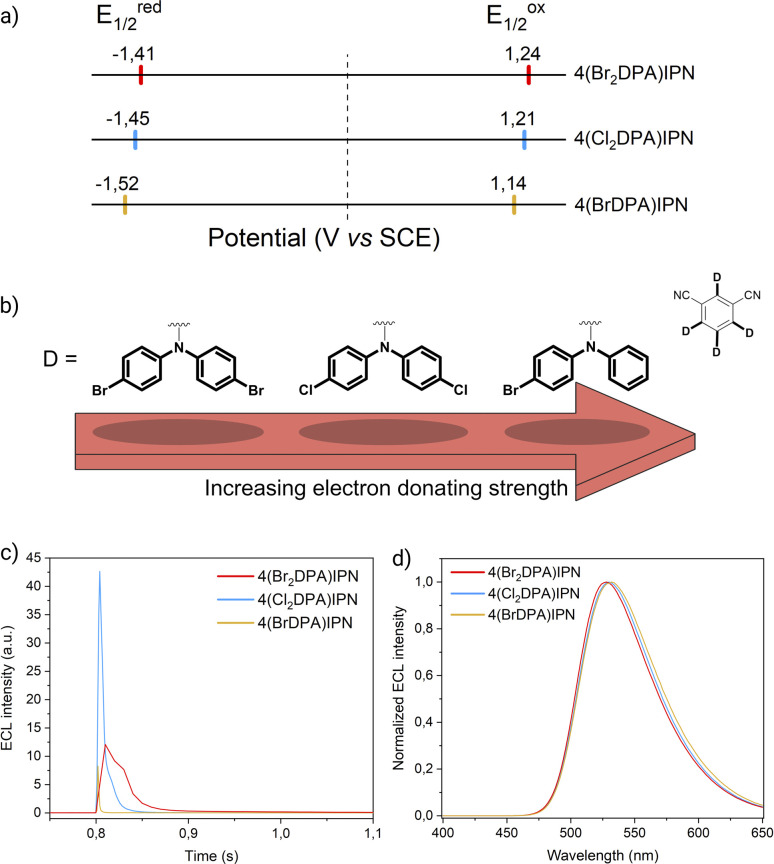
(a) Scheme showing the half-wave reduction potentials (*E*_1/2_^red^, left) and half-wave oxidation potentials (*E*_1/2_^ox^, right) of the donor-modified TADF luminophores. The dotted line marks the potential of 0 V. (b) Scheme representing the electron-donating strength of functionalized DPA units as determined by their oxidation potentials. (c) First peak ECL intensity of 4(BrDPA)IPN (yellow line), 4(Cl_2_DPA)IPN (blue line), and 4(Br_2_DPA)IPN (red line) (0.5 mM) in AcN/TBAPF_6_ 0.1 M collected *vs.* time. PMT bias of 750 V; 000.0 μA amplification. (d) ECL spectra of 4(BrDPA)IPN, 4(Cl_2_DPA)IPN, and 4(Br_2_DPA)IPN (same color code as in Fig. 3c) (0.5 mM) in AcN/TBAPF_6_ 0.1 M/BPO 10 mM. PMT bias of 700 V; integration time 100 ms; step 2 nm.

In contrast to the molecularly engineered cyanoarene derivatives studied in the previous paragraph, the aforementioned compounds exhibit reversible peaks throughout the anodic region. The end-capping of DPA *para* positions enhances the radical cation stability, effectively blocking the most reactive site and preventing the coupling process.^34,35^

The *E*_1/2_^ox^ of these molecules increases with both the number and the electron-withdrawing strength of the halogen substituents (*E*_1/2_^ox^(4(Br_2_DPA)IPN) = 1.24 V > *E*_1/2_^ox^(4(Cl_2_DPA)IPN) = 1.21 V > *E*_1/2_^ox^(4(BrDPA)IPN) = 1.14 V). As the environment around the DPA units becomes progressively more electron-attracting, the electron density in the donating moiety is retained to a greater extent, thereby stabilizing the HOMO. Accordingly, because of the stronger electron-withdrawing ability of the bromine atom (*σ*_p_ = +0.232) compared to the chlorine one (*σ*_p_ = +0.227),^26^ 4,4′Br_2_-DPA emerges as the weakest electron donor in this series, whereas 4Br-DPA stands out as the strongest one because of the limited capability of a single Br atom to pull electron density back (Fig. 3b). Notably, the separation between the anodic peaks is greater in molecules including halogen-substituted DPA units (*i.e.*, 4(Cl_2_DPA)IPN, 4(Br_2_DPA)IPN; Δ*E* ≈ 0.36 V) compared to the unsubstituted DPA counterparts (*i.e.*, 3DPAFIPN, 3DPA2FBN, 3DPAImIPN, 3DPA2ImBN; Δ*E* ≈ 0.15 V). This overpotential is likely due to the electron-withdrawing effect of the halogen atoms on the donors, that results in a weaker delocalization of the hole and, thus, a higher coupling energy for the second oxidation.

Mirroring the methodology applied to the formerly investigated set of molecules, the *Φ*_ECL_ was esteemed in annihilation (Fig. 3c and S33–S34†). For consistency with acceptor-modified luminophores, we exploited the same approach by pulsing a sequence of first reducing and then oxidizing potentials. Among all the donor-modified dyes, 4(BrDPA)IPN exhibits the lowest *Φ*_ECL_ by a significant margin. Conversely, 4(Cl_2_DPA)IPN and 4(Br_2_DPA)IPN display a gradual increase in *Φ*_ECL_, with the latter emerging as the overall most efficient ECL luminophore. This trend is reflected in the computed Δ*E*_ST_ (Table S3†). The inferior *Φ*_ECL_ of 4(BrDPA)IPN suggests, as evidenced by its small PLQY, the presence of additional non-radiative excited state deactivation pathways, possibly introduced by monohalogenation. However, the instability of the ECL signal across multiple annihilation cycles suggests that other factors beyond excited state deactivation may hinder the overall effectiveness of the ECL process. In fact, while monobromination appears to ensure reversibility in cyclic voltammetry at 100 mV s^−1^, the 4(BrDPA)IPN radical cation might not possess sufficient stability in annihilation conditions. The remarkable *Φ*_ECL_ of 4(Cl_2_DPA)IPN and 4(Br_2_DPA)IPN arises from a synergistic interplay of photophysical and electrochemical properties. In fact, the sustained duration of the ECL signal within individual peaks, coupled with its remarkable stability over numerous annihilation cycles (*e.g.*, 4(Cl_2_DPA)IPN retains 70% of its peak intensity after 21 annihilation cycles), underscores the favorable electrochemical properties conferred by di-substitution at both DPA para positions, promoting the generation of relatively long-lasting radical ions crucial for efficient ECL emission. At the same time, halogenation appears to affect the photophysics of this class of molecules. The observed *Φ*_ECL_ improvement is consistent with the increasing molecular weight of the substituting halogen, hinting at the involvement of the internal heavy atom (IHA) effect that improves spin–orbit coupling between T_1_ and S_1_ states.^36^ Further supporting this, the shortening of the *τ*_delayed_ from 4(Cl_2_DPA)IPN to 4(Br_2_DPA)IPN directly demonstrates the IHA impact on the investigated donor-modified molecules.

Replicating the approach previously proposed, the ECL signal generated using BPO as the coreactant was recorded in CV-ECL (Fig. S35–S37†) and eventually exploited to acquire the ECL spectra reported in Fig. 3d. Importantly, the ECL spectra of the investigated molecules aligned with PL spectra, excluding the possibility of emission from a potential excimer or exciplex.

### Water-soluble TADF emitters

The cornerstone application of ECL is bioanalytical chemistry, where an aqueous environment is necessary for mimicking physiological conditions and for preserving bioanalyte stability. However, finding water-soluble luminophores with favourable redox properties for ECL remains a challenge. While imidazole groups have been demonstrated to positively affect the ECL emission of TADF compounds, they also offer a valuable scaffold for further conjugation. To address the lack of water-soluble emitters, we exploited the imidazoles of 3DPA2ImBN and 3DPAImIPN as platforms for derivatization with TEG chains. Absorption and emission spectra are presented in Fig. S38 and S39.† To assess the effectiveness of [3DPA2(ImTEG)BN](OTs)_2_ and [3DPAImTEGIPN]OTs as luminophores in bioanalytical conditions (*i.e.*, non-degassed aqueous environment), we performed CV-ECL measurements using tri-*n*-propylamine (TPrA) as coreactant. TPrA stands out as the most viable choice for ECL among tertiary amines due to several key advantages. First, TPrA can be effectively solubilized in water by meticulously adjusting the pH of the buffer solution. Second, its oxidation potential remains relatively low (*E*° = 0.78 V *vs.* SCE), preventing conflict with the oxygen evolution reaction. Most importantly, TPrA demonstrates outstanding ECL efficiency when paired with ECL-active luminophores, thanks to its optimal balance between coreactant reactivity and radicals stability.^37^ Due to TPrA sensitivity to oxidation, CV-ECL measurements were carried out by scanning the electrode potential within the anodic region.

**Fig. 4:**
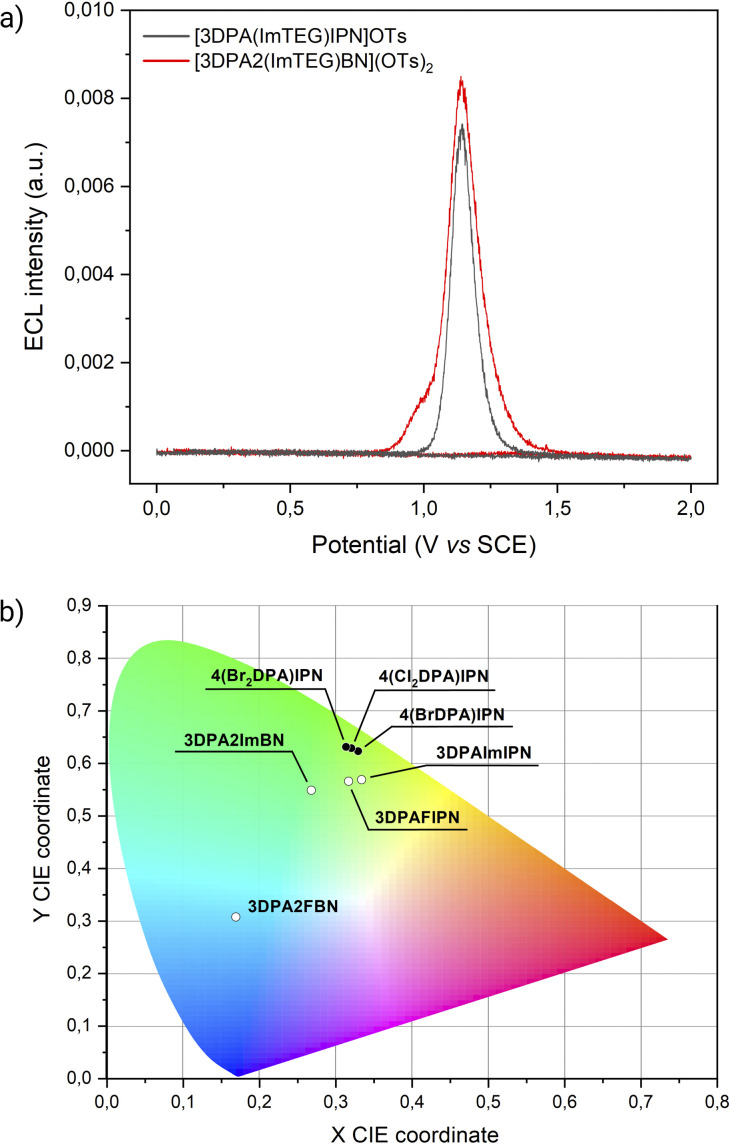
(a) ECL intensity of [3DPA(ImTEG)IPN]OTs (grey line), and [3DPA2(ImTEG)BN](OTs)_2_ (red line) (0.5 mM) in 0.3 M PB with 180 mM TPrA collected *vs.* applied potential. The CV-ECL measurement was performed at 100 mV s^−1^ by sweeping the electrode from the open circuit potential (OCP) to 2 V *vs.* SCE, then back to 0 V and finally completing the cycle by returning to OCP. PMT bias of 750 V; 000.0 μA amplification. (b) 1931 CIE chromaticity diagram showing coordinates from acceptor-modified dyes (white points) and donor-modified dyes (black points).

Both [3DPA2(ImTEG)BN](OTs)_2_ and [3DPA(ImTEG)IPN]OTs functioned as effective ECL luminophores under unusual working conditions for TADF compounds (Fig. 4a).

[3DPA2(ImTEG)BN](OTs)_2_ generated a stronger ECL signal compared to [3DPAImTEGIPN]OTs, aligning with the previously observed trend in ECL efficiency for their non-water-soluble counterparts. The most straightforward feature of both compounds is the intense ECL peak at a similar potential, around 1.2 V *vs.* SCE. Unfortunately, the limited electrochemical stability window of water prevented the determination of the oxidation potentials for [3DPA2(ImTEG)BN](OTs)_2_ and [3DPAImTEGIPN]OTs. Yet, assuming a minor and similar effect of PEGylation on both molecules, their oxidation potentials likely remain comparable, as occurs for 3DPA2ImBN and 3DPAImIPN. Therefore, the oxidation of the luminophore at the electrode surface appears to be the bottleneck for achieving strong ECL emission in both systems. This hypothesis is supported by the conventional behavior of luminophore/TPrA systems, where the peak emission in CV-ECL is achieved upon the annihilation reaction between the oxidized luminophore and the strongly reducing α-aminoalkyl radical TPrA˙, as displayed in eqn (9)–(13).

9 TPrA − e^−^ → TPrA˙^+^

10 TPrA˙^+^ − H^+^ → TPrA˙

11 TADF − e^−^ → TADF˙^+^

12 TADF˙^+^ + TPrA˙ → TADF* + Im^+^

13 TADF* → *hν* + TADF

TPrA˙ (*E*° = −1.75 V *vs.* SCE) is generated through deprotonation of the electrogenerated TPrA˙^+^. Ultimately, upon oxidation, TPrA˙ is converted into the TPrA iminium cation (referred to as Im^+^ in eqn (12)), thus ending the coreactant cycle. The aforementioned mechanism is known as “homogeneous ECL”.

While both samples display the homogeneous ECL peak, only the ECL curve of [3DPA2(ImTEG)BN](OTs)_2_ exhibits a shoulder at lower overpotential. This different curve shape suggests an alternative luminophore/coreactant interaction mechanism, described in literature as “remote ECL”, which relies solely on TPrA oxidation.^38^ The first step in this mechanism involves the homogeneous reduction of the luminophore by TPrA˙ (formed by subsequent oxidation and deprotonation of TPrA, eqn (9) and (10)), followed by homogeneous oxidation by means of TPrA˙^+^. This sequence, as depicted in eqn (14)–(16), could ultimately lead to dye excitation and radiative relaxation.

14 TADF + TPrA˙ → TADF˙^−^ + Im^+^

15 TADF˙^−^ + TPrA˙^+^ → TADF* + TPrA

16 TADF* → *hν* + TADF

The ECL signal shoulder detected for [3DPA2(ImTEG)Bn](OTs)_2_ lies in a potential region compatible with TPrA oxidation, suggesting it arises from remote ECL. However, it is well-known that the feasibility of luminophore excitation is limited to a few cases; thus, it seems reasonable to assume that the remote pathway is active for [3DPA2(ImTEG)BN](OTs)_2_ but somehow forbidden for [3DPA(ImTEG)IPN]OTs. To elucidate the origins of this behavior, the thermodynamics of the luminophore/TPrA tandem system outline a series of conditions required for excitation, as described by eqn (17) where it is assumed to deal with reducible luminophores by TPrA˙.^39^ The resulting change in free energy governs the feasibility of the remote mechanism.17
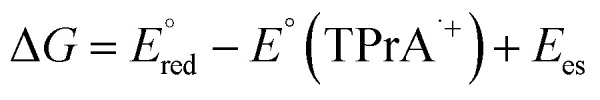



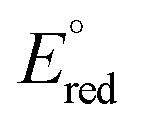
 is considered as the half-wave reduction potential in acetonitrile for the parent molecules 3DPA2ImBN and 3DPAImIPN, assuming a minor influence from the PEGylation. *E*°(TPrA˙^+^) is set at 0.78 V *vs.* SCE, and the energy gap for [3DPA2(ImTEG)BN](OTs)_2_ and [3DPA(ImTEG)IPN]OTs was estimated from the corresponding emission spectra in acetonitrile. The energy gap values were determined to be 2.3 eV for the diimidazole derivative and 2.23 eV for the monoimidazole derivative. Plugging in the aforementioned values in eqn (17) yields Δ*G* values of −0.16 eV and −0.04 eV, respectively. While negative Δ*G* values typically indicate thermodynamically feasible processes, remote ECL is nevertheless observed only for [3DPA2(ImTEG)BN](OTs)_2_. This discrepancy likely arises from the approximation associated with using the reduction potentials of the parent molecules and from the difference in polarity between the environment used for energy gap estimation and for the actual ECL measurement. Notably, the Δ*G* value for [3DPA2(ImTEG)BN](OTs)_2_ is 120 meV more negative compared to that of [3DPA(ImTEG)IPN]OTs, explaining why remote ECL is observed only for the diimidazole derivative.

Notably, the stability of the ECL signal improves from [3DPA(ImTEG)IPN]OTs to [3DPA2(ImTEG)BN](OTs)_2_ (Fig. S40–S41†), highlighting a stabilizing effect of the imidazole as the same trend is observed in their parent molecules (Fig. S20 and S21†). However, both luminophores show poor overall stability, possibly due to electrode passivation during DPA oxidation.^25^

Clinical ECL assays mostly exploit magnetic beads as platforms for attaching ECL labels, hindering their heterogeneous oxidation and thus limiting the ECL process to rely solely on the remote mechanism. We demonstrated that [3DPA2(ImTEG)BN](OTs)_2_ and potentially a broader range of TADF dyes, if strategically engineered with conjugation functionalities, hold promise as cost-effective emitters to replace traditional [Ru(bpy)_3_]^2+^ labels.

## Conclusions

In this work, we conducted a systematic investigation to elucidate how substituents engineering impacts the ECL efficiency of TADF dyes. In particular, we explored two classes of luminophores by separately tuning the electron accepting power of the cyanoarene rings and the electron donating strength of the DPA groups. Through a meticulous comparison of photophysical, redox, ECL, and DFT data, we deduced the impact of given substituents on the ECL behavior of the designed TADF molecules.

The electron-accepting series display significant differences in redox properties, in particular the reduction potentials, which is translated into a wide range of emission wavelengths (Fig. 4b). Their ECL efficiencies experience the same effect, yielding the highest value for 3DPA2ImBN thanks to a stronger CT character of the excited state.

In contrast, the halogenation of electron-donating DPA units affects oxidation and reduction potentials to similar extents, mostly retaining the same emission wavelength throughout the series. On the other hand, the ECL efficiency progressively improves by increasing the number and atomic weight of the incorporated halogens. This effect arises from preventing coupling reactions among DPA radicals and increasing the RISC rate.

While tuning electron-accepting strength seems to primarily affect molecular orbitals, tuning the electron-donating strength improves redox stability and coupling between the T_1_ and S_1_ excited states. These findings suggest that the proposed approaches represent parallel strategies, devoted to enhancing TADF ECL efficiency by affecting different aspects of the luminophore. This opens doors for designing TADF dyes with broad applicability in ECL devices.

Ultimately, we successfully synthesized water-soluble TADF dyes by attaching TEG chains to imidazole rings. Characterization under conventional conditions relevant to commercial ECL bioassays revealed that [3DPA2(ImTEG)BN](OTs)_2_ exhibited promising potential as a cheaper alternative to the established [Ru(bpy)_3_]^2+^ as ECL label in bead-based assays.

## Data availability

Experimental data about the electrochemistry and the ECL properties of the investigated luminophores are available at AMS Acta at https://amsacta.unibo.it/id/eprint/7801. CCDC 2359842 contains the supplementary crystallographic data for 3DPA2ImBN.

## Author contributions

F. C. performed the synthesis of donor-modified compounds. F. C. and A. F. performed the photophysical characterizations of the donor-modified luminophores and analyzed the data. G. P. and T. S. performed the synthesis, photophysical characterizations of imidazole-derivatized emitters and analyzed the data. A. F. and P. N. performed the ECL experiments. F. N. performed the DFT calculations. N. D. performed the XRD analysis. P. C., F. P., P. G. C., F. N., A. G., A. A. and G. V. directed the study and designed the research. All authors prepared the manuscript and approved the final version.

## Conflicts of interest

There are no conflicts to declare.

## Supplementary Material

SC-OLF-D4SC04986A-s001

SC-OLF-D4SC04986A-s002
