# Supplementary material for: Tunable electrochemiluminescence of TADF luminophores: manipulating efficiency and unveiling water-soluble emitters
Source: Chem Sci. 2024 Oct 3;15(43):17892–9. doi: 10.1039/d4sc04986a (PMC11462160; doi:10.1039/d4sc04986a)
Supplement: SC-015-D4SC04986A-s001 [file SC-015-D4SC04986A-s001.pdf]

Supplementary Information (SI) for Chemical Science.
This journal is © The Royal Society of Chemistry 2024

Tunable electrochemiluminescence of TADF luminophores: manipulating efficiency and unveiling water-soluble emitters

Alessandro Fracassa,[a] Francesco Calogero,[a,b] Giulio Pavan,[c] Pavlos Nikolaou,[a] Andrea Fermi,[a,b] Paola Ceroni,[a,b] Francesco Paolucci,[a,b] Pier Giorgio Cozzi,[a,b] Thomas Scattolin,[c] Nicola Demitri,[d] Fabrizia Negri,[a] Andrea Gualandi,*[a,b] Alessandro Aliprandi*[c] and Giovanni Valenti*[a,b]

a. Dipartimento di Chimica "Giacomo Ciamician", Alma Mater Studiorum – Università di Bologna, Via Gobetti 85, 40129 Bologna, Italy.

b. Center for Chemical Catalysis – C3, Alma Mater Studiorum – Università di Bologna, Via Gobetti 85, 40129 Bologna, Italy.

c. Dipartimento di Scienze Chimiche, Università degli Studi di Padova, Via Marzolo 1, 35122 Padova, Italy.

d. Elettra – Sincrotrone Trieste, S.S. 14 Km 163.5 in Area Science Park, 34149 Basovizza – Trieste, Italy.

Electronic Supporting Materials

Table of contents

Experimental Procedure 3
General Methods and Materials 3
Synthesis of Diphenylamines 4
Synthesis of TADF Dyes 5
Copies of NMR Spectra 11
Experimental information 21
Photophysical characterization of acceptor-modified compounds 21
Photophysical characterization of donor-modified compounds 21
Electrochemical characterization 22
Electrochemiluminescence characterization 22
Computational details 23
Photophysics of acceptor-modified compounds 24
Absorption spectra 24
Emission spectra 25
Excitation spectra 26
Computed DFT data for acceptor-modified molecules 28
XRD Analysis – Structural characterization of 3DPA2ImBN 33
Electrochemistry of acceptor-modified compounds 36
Electrochemiluminescence of acceptor-modified compounds 37
Annihilation ECL 37
Coreactant CV-ECL of acceptor-modified compounds 41
Photophysics of donor-modified compounds 45
Absorption, emission spectra and lifetimes 45
Computed DFT data for donor-modified molecules 54
Electrochemistry of donor-modified compounds 59
Electrochemiluminescence of donor-modified compounds 60
Annihilation ECL 60
Coreactant CV-ECL of donor-modified compounds 62
Photophysical characterization of water-soluble compounds 65
Absorption spectra 65
Emission spectra 66
ECL signal stability of water-soluble compounds 67
References 70

Experimental Procedure

General Methods and Materials

^{1}H NMR spectra were recorded on Varian Mercury 400, Bruker Ascend 600 or Bruker AVANCE III 300 (equipped with a BBO probe) spectrometers. Chemical shifts are reported in ppm from TMS with the solvent resonance as the internal standard ($CDCl_3$: δ = 7.24 ppm, CD_3CN: δ = 1.94 ppm, CD_2Cl_2: δ = 5.32 ppm). Data are reported as follows: chemical shift, multiplicity (s = singlet, d = doublet, t 0 triplet, q = quartet, m= multiplet), coupling constants (Hz), number of protons. {^{1}H}^{13}C NMR spectra were recorded on Varian Mercury 400, Bruker Ascend 600 or Bruker AVANCE III 300 (equipped with a BBO probe) spectrometers. spectrometer or Bruker Ascend 600 spectrometer. Chemical shifts are reported in ppm from TMS with the solvent as the internal standard ($CDCl_3$: δ = 77.0 ppm, DMSO-d_6: δ = 39.5 ppm, CD_3CN: δ = 1.32, CD_2Cl_2: δ = 53.84 ppm). ^{19}F NMR and {^{1}H}^{19}F NMR were recorded with a Bruker 200 MHz equipped with a QNP probe; ^{19}F chemical shifts were referenced using indirect referencing.[1]

All reactions were monitored by thin-layer chromatography using Merck silica gel plates 60 F254 (plastic sheets) and spots were visualized with UV light or with iodine/silica staining.

Chromatographic purifications were done with 240-400 mesh silica gel or were performed on a Combiflash Nextgen 300+ using Redisep Bronze column (4, 12, 24, 40 grams) filled with NP silica gel and using Redisep Rf cartridge (4 and 24 grams).

All reactions were set up under an argon atmosphere in oven-dried glassware using standard Schlenk techniques.

All the reagents were purchased from commercial sources (Sigma-Aldrich, Alfa Aesar, Fluorochem, Strem Chemicals, TCI) and used without further purification unless specified. All solvents, except those for photophysical measurements, were dried according to literature[2] using 3Å molecular sieves purchased from Thermoscientific.

3,6-di-*tert*-butyl-carbazole[3] and 2-(2-(2-methoxyethoxy)ethoxy)ethyl4-methylbenzenesulfonate[4] were synthesized according to literature procedure.

Synthesis of Diphenylamines

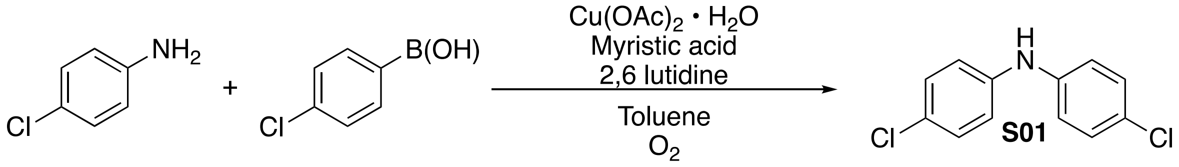

Diphenyl amine (**S01**) was prepared adapting the procedure reported in literature.[5]

To a solution of 4-chlorophenylboronic acid (1.28 mmol, 200 mg, 1.5 equiv.) in toluene (2 mL) under nitrogen atmosphere, copper diacetate hydrate (0.085 mmol, 17 mg, 0.1 equiv.), myristic acid (0.17 mmol, 39 mg, 0.2 equiv.), 2,6-lutidine (0.85 mmol, 91 μL, 1 equiv.) and 4-chloroaniline (0.85 mmol, 109 mg, 1 equiv.) were added. The flask was evacuated and filled with O_2 (balloon) and the reaction mixture was vigorously stirred overnight.

Water (10 mL) was added and the mixture was extracted with AcOEt (3 x 15 mL). The organic layers were dried over anhydrous Na_2SO_4 and the solvent evaporated under reduced pressure to give a red oil. Product **S01** was obtained as yellow oil (72%, 0.6 mmol, 146 mg) after chromatographic purification (SiO_2, cyclohexane:AcOEt, 15:1).

Spectroscopic data agree with those reported in literature.[6]

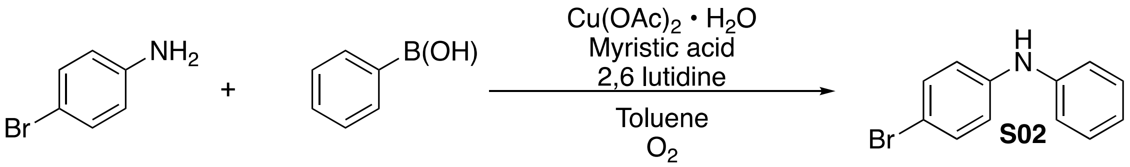

Diphenyl amine (**X**) was prepared adapting the procedure reported in literature.[5]

To a solution of phenylboronic acid (6 mmol, 731 mg, 1.5 equiv.) in toluene (5 mL) under nitrogen atmosphere, copper diacetate hydrate (0.4 mmol, 79 mg, 0.1 equiv.), myristic acid (0.8 mmol, 183 mg, 0.2 equiv.), 2,6-lutidine (4 mmol, 460 μL, 1 equiv.) and 4-bromooaniline (4 mmol, 688 mg, 1 equiv.) were added. The flask was evacuated and filled with O_2 (balloon) and the reaction mixture was vigorously stirred overnight.

Water (15 mL) was added and the mixture was extracted with AcOEt (3 x 15 mL). The organic layers were dried over anhydrous Na_2SO_4 and the solvent evaporated under reduced pressure to give a red oil. Product **S02** was obtained as yellow oil (79%, 3.2 mmol, 794 mg) after chromatographic purification (SiO_2, cyclohexane:AcOEt, 10:1).

Spectroscopic data agree with those reported in literature.[7]

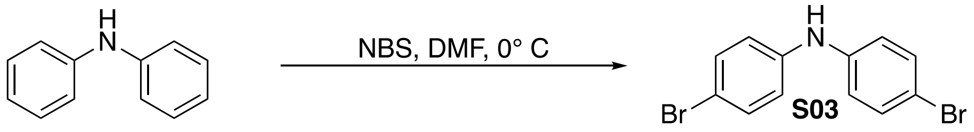

Diphenyl amine (**S03**) was prepared adapting the procedure reported in literature.[8]

To a solution of diphenylamine (3 mmol, 507 mg, 1 equiv.) in DMF (10 mL) at 0°C, a solution of NBS (6 mmol, 1.06 g, 1 equiv.) in DMF (3 mL) was added dropwise in 30 minutes.

The reaction mixture was stirred at 0°C for 7 hours and the solvent was evaporated under reduced pressure. Product **S03** was obtained as white solid (83%, 2.5 mmol, 814 mg) after chromatographic purification (SiO_2, cyclohexane:AcOEt, 20:1).

Spectroscopic data agree with those reported in literature.[5]

Synthesis of TADF Dyes

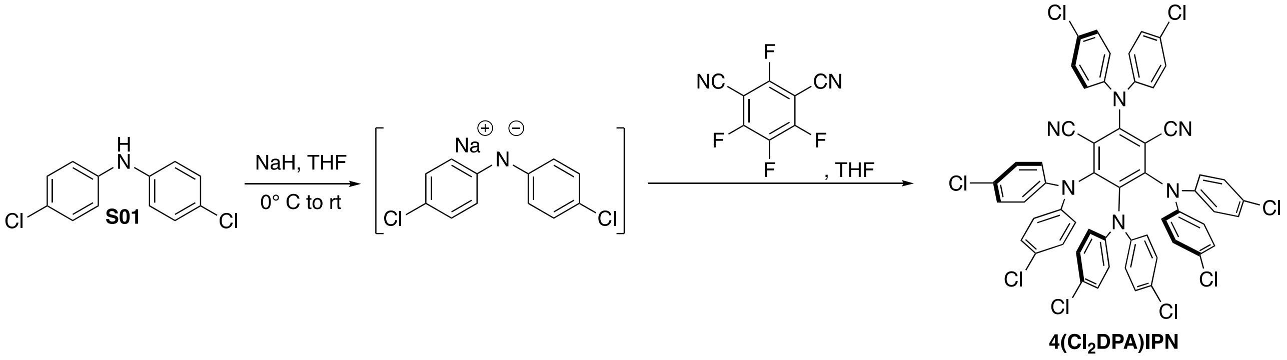

To a solution of diphenylamine **S01** (0.42 mmol, 100 mg, 5 equiv.) in dry THF (2 mL) at 0 °C, NaH (60% in mineral oil, 0.63 mmol, 25 mg, 7.5 equiv.) was slowly added under vigorous stirring. The temperature was slowly raised to 50°C and the mixture was stirred for 2 hours. Tetrafluoroisophtalonitrile (0.084 mmol, 17 mg, 1 equiv.) was added at room temperature and the mixture was stirred at the same temperature. The solution slowly turned from colorless to dark brown. When the TLC showed a complete consumption of the starting material (usually 2 days are needed), water (0.5 mL) was added to neutralize the excess of NaH and the mixture was evaporated to give a yellow solid. Product **4(Cl_2DPA)IPN** was obtained as yellow sticky solid (46%, 0.039 mmol, 42 mg) after chromatographic purification (SiO_2, cyclohexane:DCM, from 7:3 to 5:5).

Spectroscopic data agree with those reported in literature.[6]

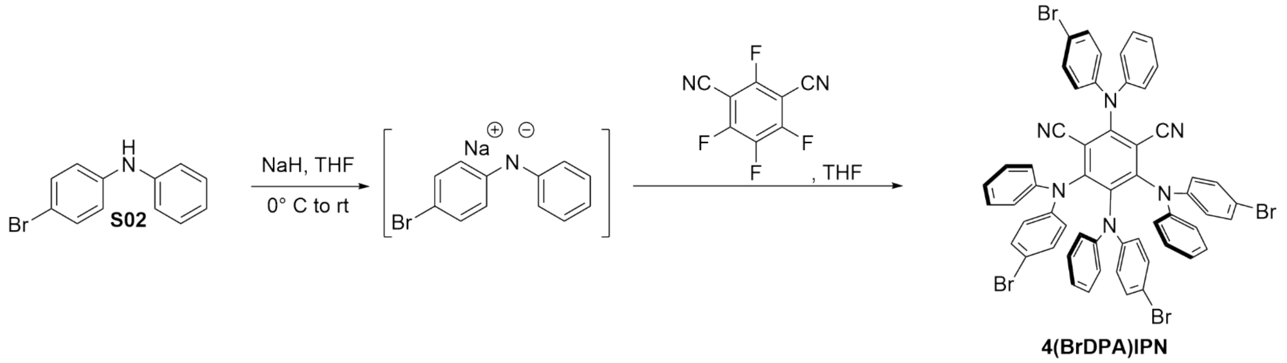

To a solution of diphenylamine **S02** (0.5 mmol, 124 mg, 5 equiv.) in dry THF (4 mL) at 0 °C, NaH (60% in mineral oil, 1.5 mmol, 30 mg, 7.5 equiv.) was slowly added under vigorous stirring. The temperature was slowly raised to 50°C and the mixture was stirred for 2 hours. Tetrafluoroisophtalonitrile (0.1 mmol, 20 mg, 1 equiv.) was added at room temperature and the mixture was stirred at the same temperature. The solution slowly turned from colorless to dark brown. When the TLC showed a complete consumption of the starting material (usually 2 days are needed), water (0.5 mL) was added to neutralize the excess of NaH and the mixture was evaporated to give a yellow solid. Product **4(BrDPA)IPN** was obtained as yellow sticky solid (38%, 0.038 mmol, 42 mg) after chromatographic purification (SiO_2, cyclohexane:DCM, from 7:3 to 6:4).

^{1}H NMR (600 MHz, $CDCl_3$) δ 7.38 – 7.33 (m, 2H), 7.31 – 7.25 (m, 2H), 7.17 – 7.10 (m, 8H), 7.08 (t, J = 7.4 Hz, 1H), 7.04 – 6.98 (m, 4H), 6.95 (q, J = 8.0 Hz, 4H), 6.92 – 6.87 (m, 2H), 6.78 – 6.74 (m, 1H), 6.68 (d, J = 7.9 Hz, 4H), 6.50 (d, J = 8.0 Hz, 2H), 6.47 (d, J = 8.7 Hz, 4H), 6.35 (d, J = 8.8 Hz, 2H).

^{13}C NMR (151 MHz, $CDCl_3$) δ: 153.6, 151.5, 145.0, 144.5, 143.00, 143.97, 143.8, 142.7, 142.2, 139.6, 132.6, 131.7, 130.7, 129.6, 129.0, 128.0, 125.1, 125.0, 124.3, 123.6, 123.25, 123.16, 122.3, 121.0, 117.4, 116.6, 115.0, 112.84, 112.79.

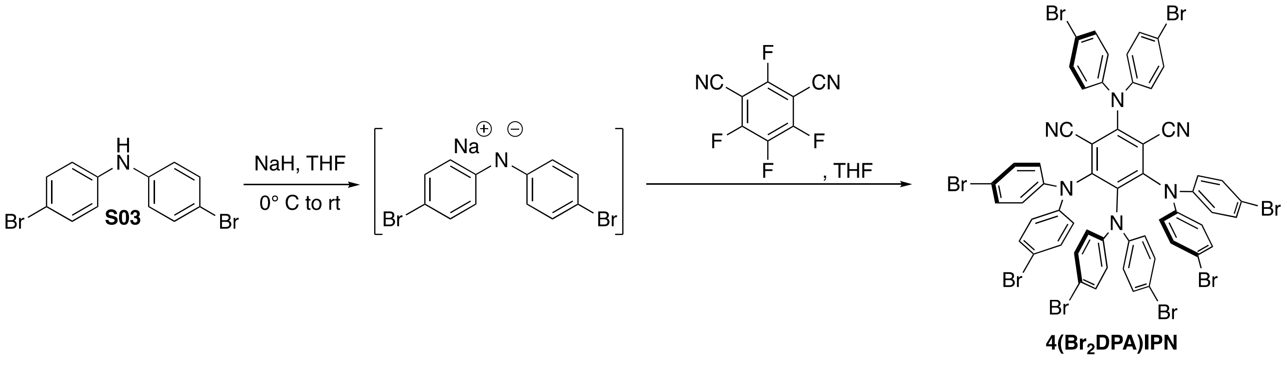

To a solution of diphenylamine **S03** (0.5 mmol, 163 mg, 5 equiv.) in dry THF (4 mL) at 0 °C, NaH (60% in mineral oil, 1.5 mmol, 30 mg, 7.5 equiv.) was slowly added under vigorous stirring. The temperature was slowly raised to 50°C and the mixture was stirred for 2 hours. Tetrafluoroisophtalonitrile (0.1 mmol, 20 mg, 1 equiv.) was added at room temperature and the mixture was stirred at the same temperature. The solution slowly turned from colorless to red. When the TLC showed a complete consumption of the starting material (usually 2 days are needed), water (0.5 mL) was added to neutralize the excess of NaH and the mixture was evaporated to give a yellow solid. Product **4(Br_2DPA)IPN** was obtained as yellow sticky solid (48%, 0.048 mmol, 69 mg) after chromatographic purification (SiO_2, cyclohexane:DCM, from 8:2 to 6:4).
Spectroscopic data agree with those reported in literature.[6]

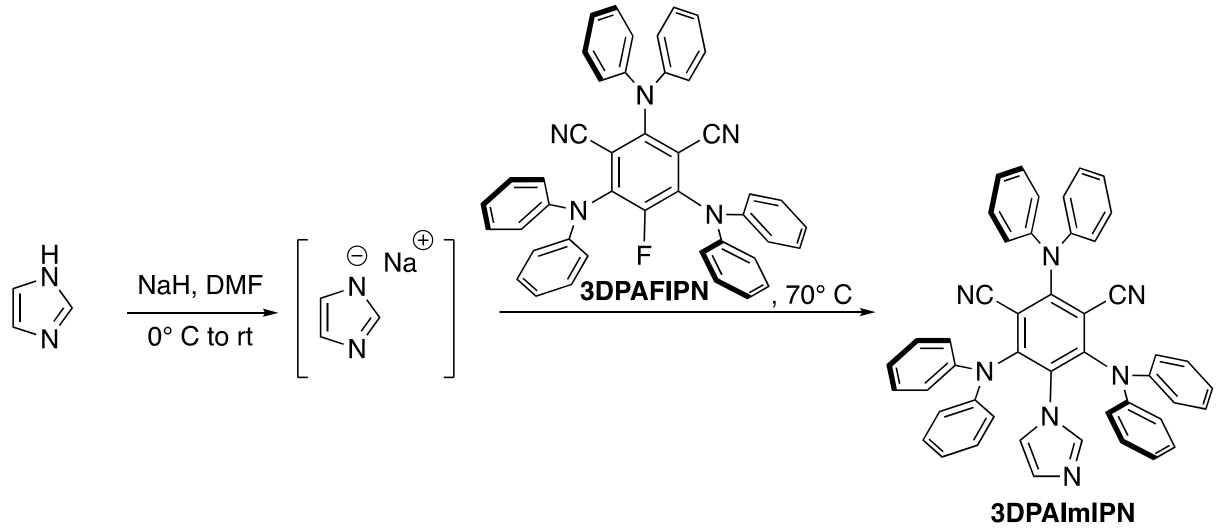

To a solution of imidazole (0.5 mmol, 31 mg, 2 equiv.) in dry *N*,*N*-dimethylformamide (5 mL) at room temperature, NaH (60% in mineral oil, 0.4 mmol, 16 mg, 1.6 equiv.) was slowly added under vigorous stirring.

After 25 minutes 2,4,6-tris(diphenylamino)-5-fluoroisophthalonitrile (**3DPAFIPN**, 0.25 mmol, 159 mg, 1 equiv.) is added and the temperature is raised to 70 °C. The solution turned dark green. When the TLC showed a complete consumption of the starting material (18 hours) water (1 mL) was added to quench the reaction. The solvent was then evaporated, the mixture extracted thrice with dichloromethane, the organic phase dried with sodium sulphate and the solvent was evaporated to give a dark green solid.

Product **3DPAImIPN** was obtained as a yellow solid (92%, 0.23 mmol, 162 mg) after chromatographic purification (SiO_2, dichloromethane:methanol, from 10:0 to 9:1).

^{1}H NMR (300 MHz, CD_3CN) δ 7.40 – 7.25 (m, 4H), 7.25 – 7.06 (m, 14H), 7.04 – 6.94 (m, 4H), 6.93 – 6.83 (m, 8H), 6.48 (s, 1H), 6.27 (s, 1H), 6.18 (s, 1H).

^{13}C NMR (75 MHz, CD_3CN) δ 156.41, 154.36, 146.35, 145.79, 137.38, 133.55, 130.42, 130.16, 129.29, 125.35, 124.83, 124.07, 123.05, 120.38, 118.26, 113.94, 112.22.

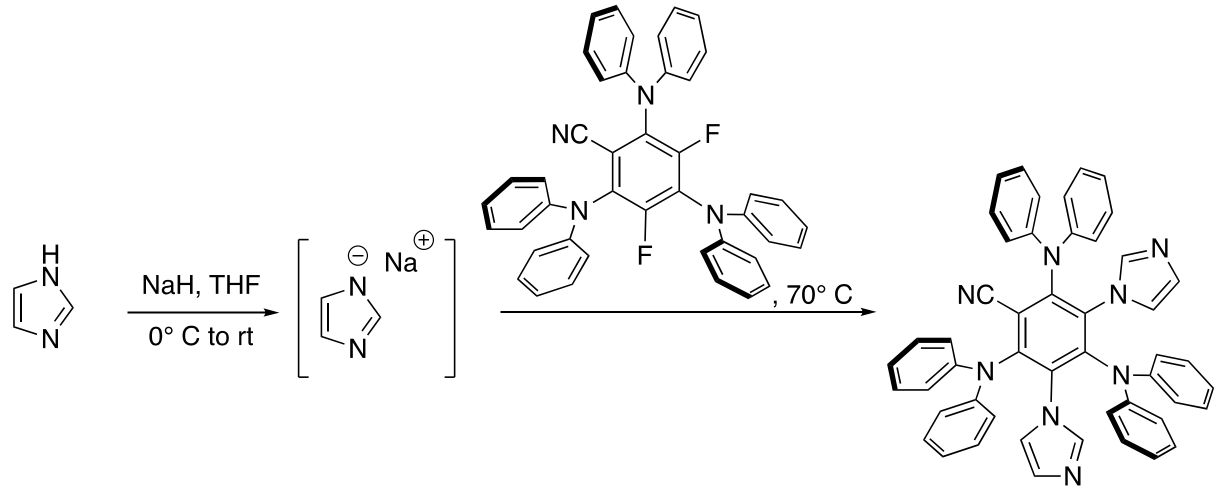

3DPA$_2$ImBN

To a solution of imidazole (1.16 mmol, 79 mg, 2.9 equiv.) in dry *N*,*N*-dimethylformamide (10 mL) at room temperature, NaH (60% in mineral oil, 1.3 mmol, 53 mg,3.3 equiv.) was slowly added under vigorous stirring.

After 25 minutes 2,4,6-tris(diphenylamino)-3,5-difluorobenzonitrile (0.4 mmol, 260 mg, 1 equiv.) is added and the temperature is raised to 70 °C. The solution turned dark green. When the TLC showed a complete consumption of the starting material and the presence of only one product (24 hours are needed at least) water (1 mL) was added to quench the reaction. The solvent was then evaporated, the mixture extracted thrice with dichloromethane, the organic phase dried with sodium sulphate and the solvent was evaporated to give a dark green solid.

Product **3DPA2ImBN** was obtained as a yellow solid (92%, 0.23 mmol, 162 mg) after chromatographic purification (SiO_2, dichloromethane:methanol, from 10:0 to 9:1).

^{1}H NMR (300 MHz, CD_3CN) δ 7.30 – 7.16 (m, 4H), 7.12 – 7.02 (m, 3H), 7.02 – 6.87 (m, 6H), 6.79 – 6.71 (m, 2H), 6.35 (dt, J = 5.4, 1.3 Hz, 2H), 6.31 (d, J = 1.1 Hz, 1H).

^{13}C NMR (75 MHz, CD_3CN) δ 151.22, 150.15, 146.27, 146.16, 137.43, 136.36, 130.31, 130.06, 128.97, 124.46, 124.32, 122.63, 122.33, 120.39, 114.89, 114.64.

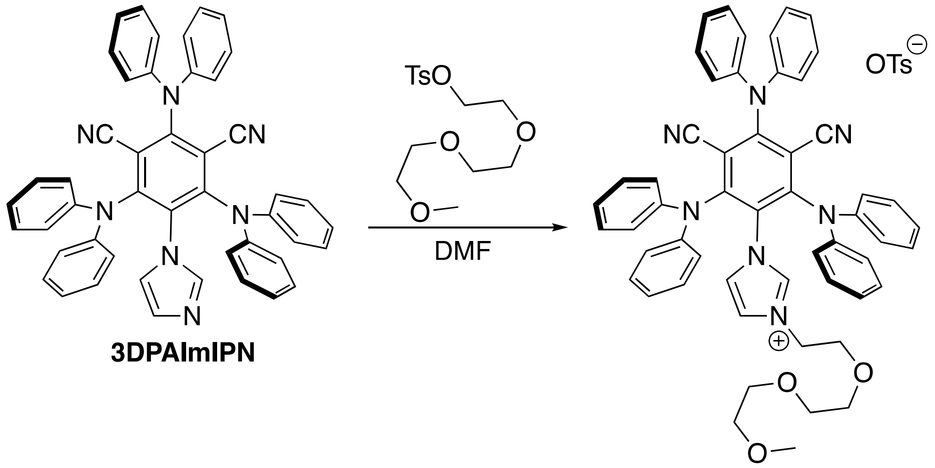

A solution of 2,4,6(diphenylamino)-5-(1*H*-imidazol-1-yl)isophthalonitrile (0.14 mmol, 100 mg, 1 equiv.) and 2-(2-(2-methoxyethoxy)ethoxy)ethyl 4-methylbenzenesulfonate (0.7 mmol, 228 mg, 5 equiv.) in dry *N*,*N*-dimethylformamide (10 mL) was stirred at 130 °C.

When the TLC showed a complete consumption of the starting material (usually 6 days are needed) solvent was evaporated and the product **[3DPA(ImTEG)IPN]OTs** was obtained as a yellow solid (43%, 0.06 mmol, 61 mg) after chromatographic purification (SiO_2, dichloromethane:methanol, from 10:0 to 9:1).

^{1}H NMR (300 MHz, CD_3CN) δ 7.91 (s, 1H), 7.62 (d, *J* = 8.1 Hz, 2H), 7.43 – 6.85 (m, 32H), 6.53 (t, *J* = 1.9 Hz, 1H), 3.85 – 3.66 (m, 2H), 3.66 – 3.03 (m, 12H).

^{13}C NMR (75 MHz, CD_3CN) δ 158.28, 154.16, 146.41, 145.67, 139.52, 138.66, 130.76, 130.59, 129.29, 127.08, 126.75, 125.96, 125.91, 125.06, 124.64, 124.29, 124.07, 118.32, 113.52, 110.13, 72.56, 70.98, 70.95, 70.65, 68.43, 58.92, 50.46, 21.28.

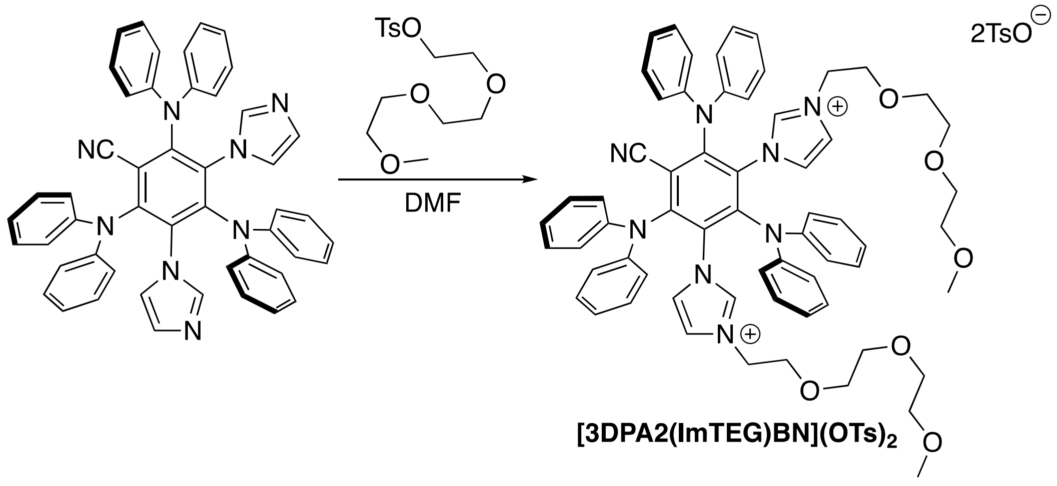

[3DPA2(ImTEG)BN](OTs)$_2$

A solution of 2,4,6(diphenylamino)-3,5-di(1*H*-imidazol-1-yl)benzonitrile (0.14 mmol, 103 mg, 1 equiv.) and 2-(2-(2-methoxyethoxy)ethoxy)ethyl 4-methylbenzenesulfonate (0.7 mmol, 228 mg, 5 equiv.) in dry *N*,*N*-dimethylformamide (10 mL) was stirred at 130 °C.

When the TLC showed a complete consumption of the starting material (usually 6 days are needed) solvent was evaporated and the product **[3DPA2(ImTEG)BN](OTs)$_2$** was obtained as a yellow solid (36%, 0.05 mmol, 69 mg) after chromatographic purification (SiO_2, dichloromethane:methanol, from 10:0 to 9:1).

^{1}H NMR (300 MHz, Acetone-d_6) δ 8.88 (s, 1H), 8.37 (s, 1H), 7.97 – 7.77 (m, 5H), 7.42 – 7.05 (m, 30H), 7.05 – 6.90 (m, 7H), 6.63 (s, 1H), 4.03 (d, *J* = 7.2 Hz, 4H), 3.66 (s, 4H), 3.52 (dd, *J* = 5.7, 3.6 Hz, 4H), 3.40 (dt, *J* = 10.4, 5.3 Hz, 14H), 3.22 (s, 6H), 2.37 (s, 6H).

^{13}C NMR (75 MHz, Acetone-d_6) δ 153.50, 150.05, 147.23, 146.79, 146.38, 145.95, 139.93, 139.62, 139.01, 130.97, 130.67, 130.39, 130.27, 129.89, 129.03, 128.81, 127.05, 125.71, 125.39, 125.22, 125.08, 124.98, 124.25, 124.20, 124.03, 123.59, 123.36, 123.24, 122.76, 122.01, 113.40, 72.56, 70.98, 70.90, 70.85, 70.81, 68.75, 58.84, 50.42, 21.29.

Copies of NMR Spectra

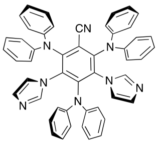

^{1}H NMR (300 MHz, CD_3CN)

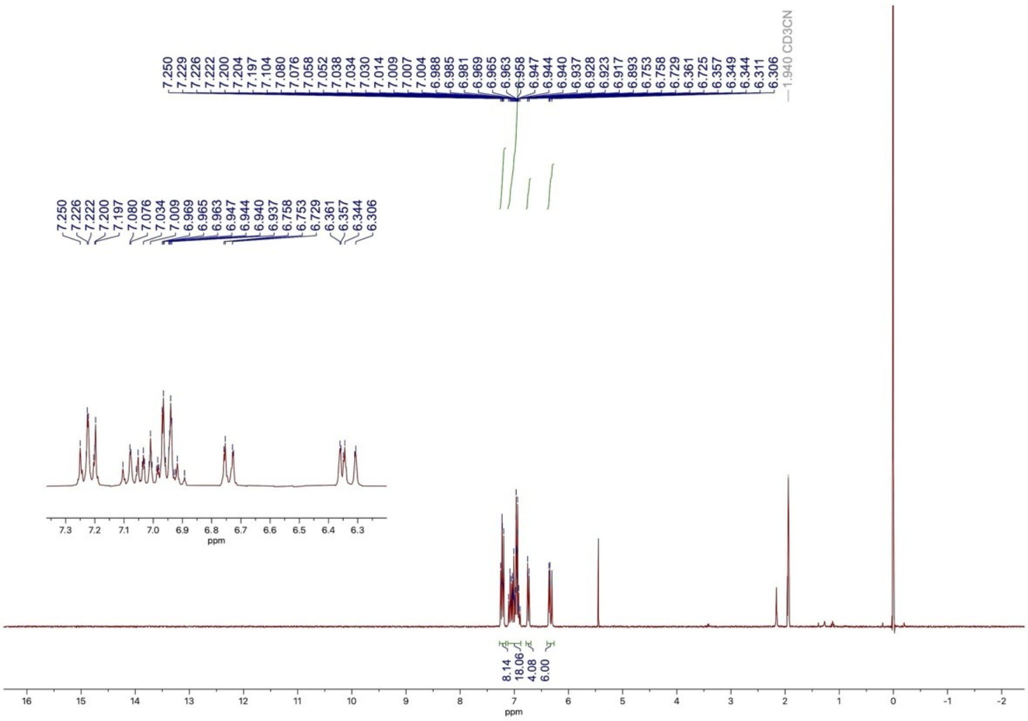

{^{1}H}^{13}C NMR (75 MHz, CD_3CN)

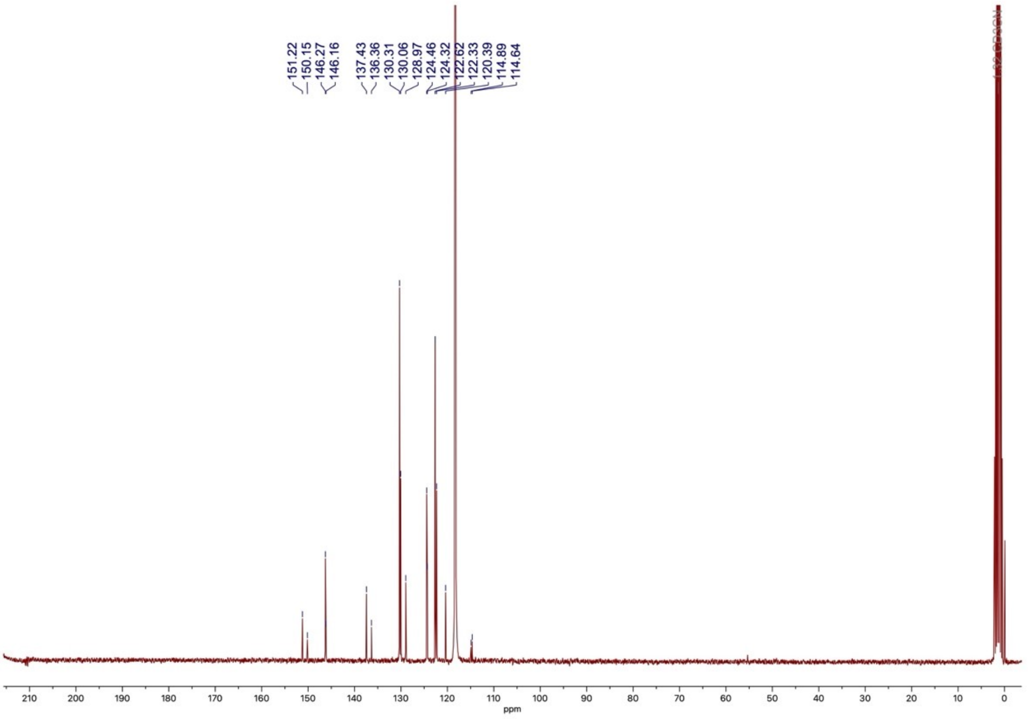

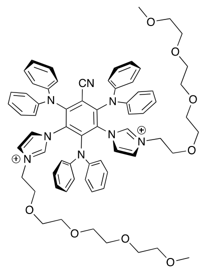

^{1}H NMR (300 MHz,d_6-acetone)

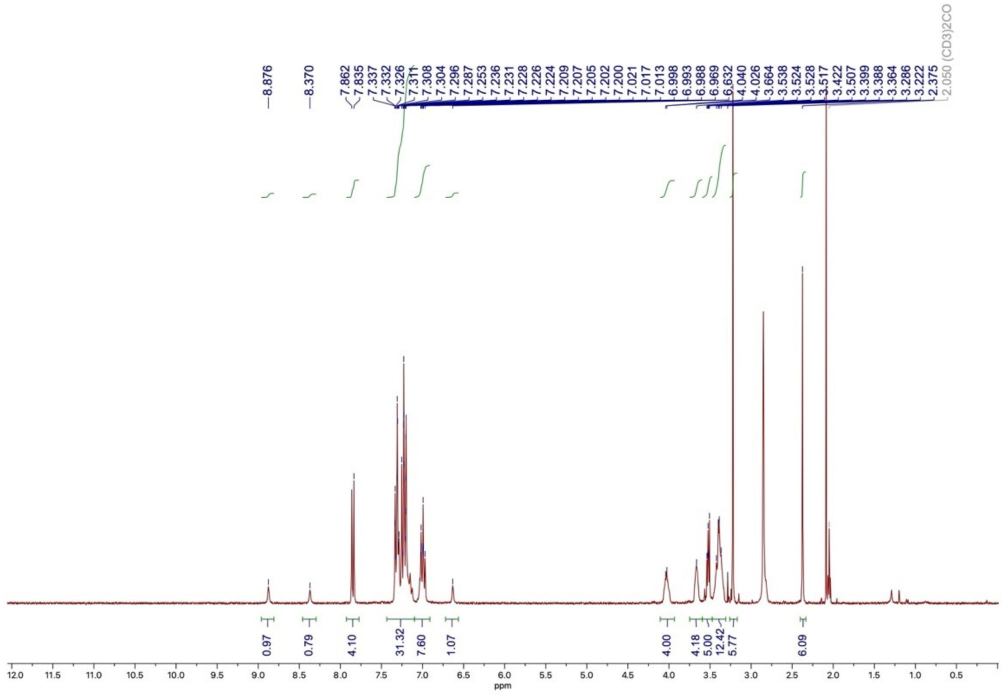

{^{1}H}^{13}C NMR (75 MHz, d_6-acetone)

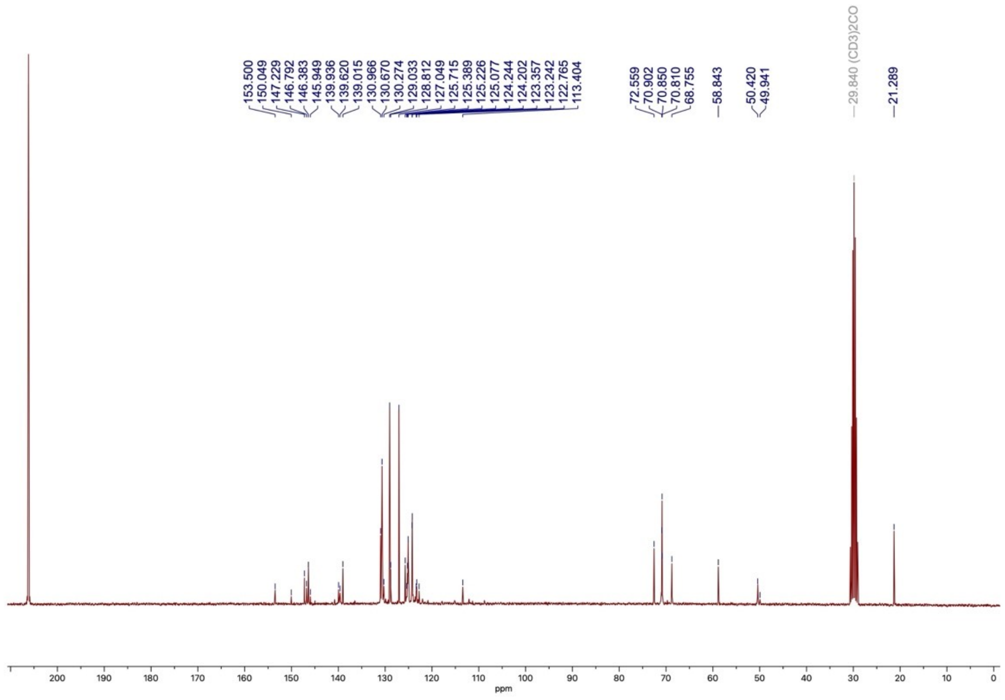

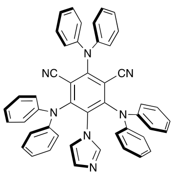

^{1}H NMR (300 MHz, CD_3CN)

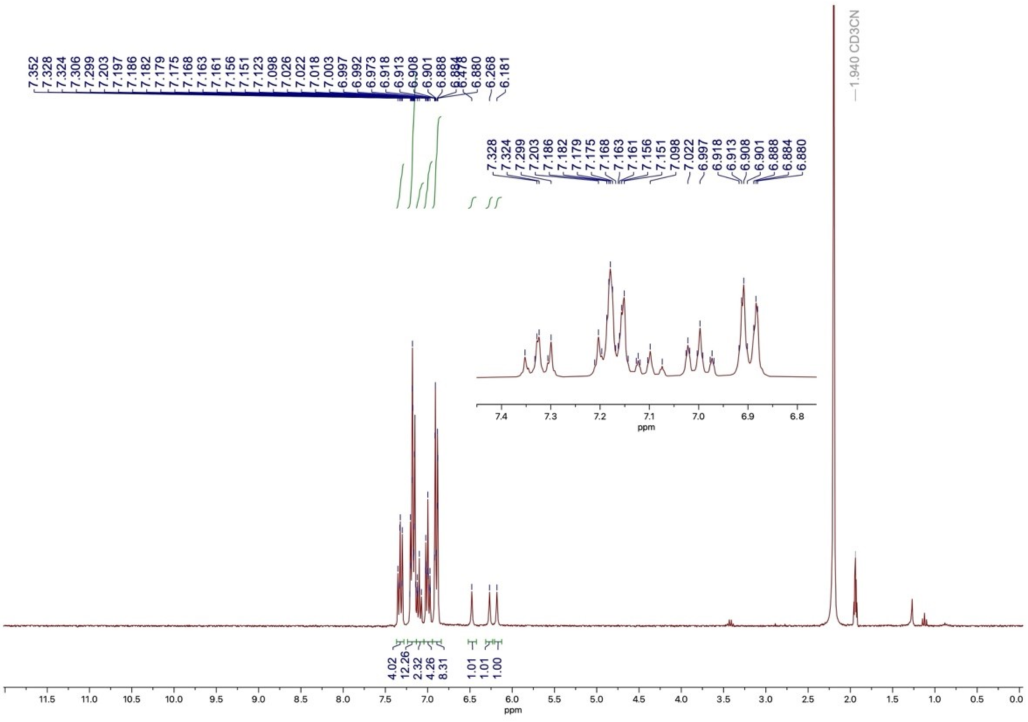

{^{1}H}^{13}C NMR (75 MHz, CD_3CN)

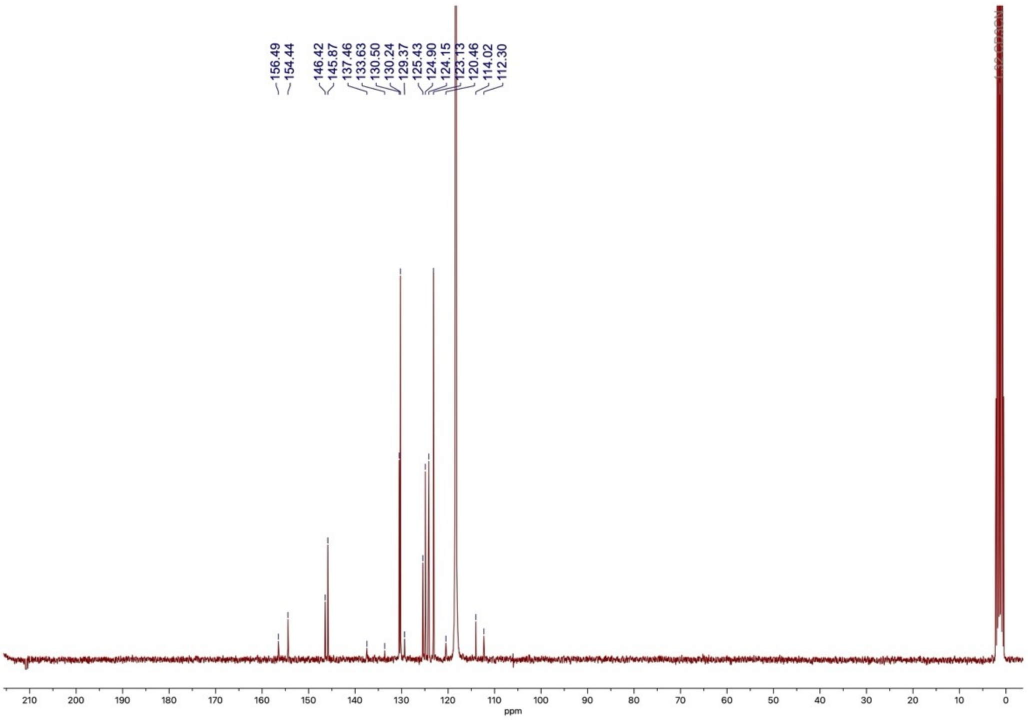

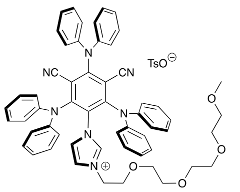

^{1}H NMR (300 MHz, CD_3CN)

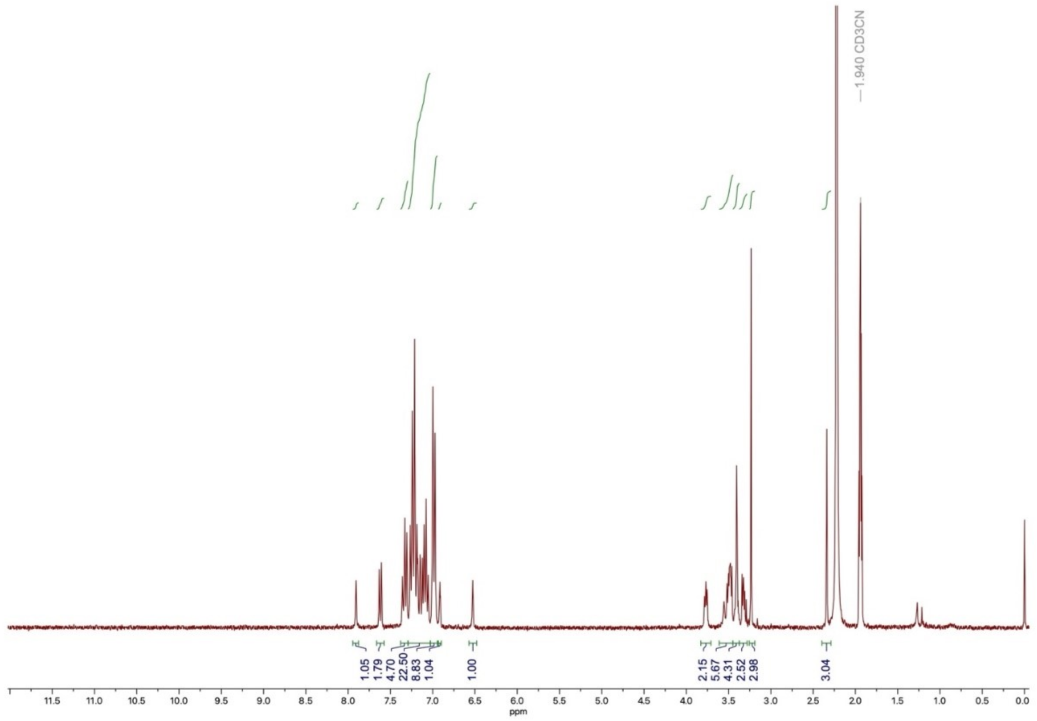

{^{1}H}^{13}C NMR (75 MHz, CD_3CN)

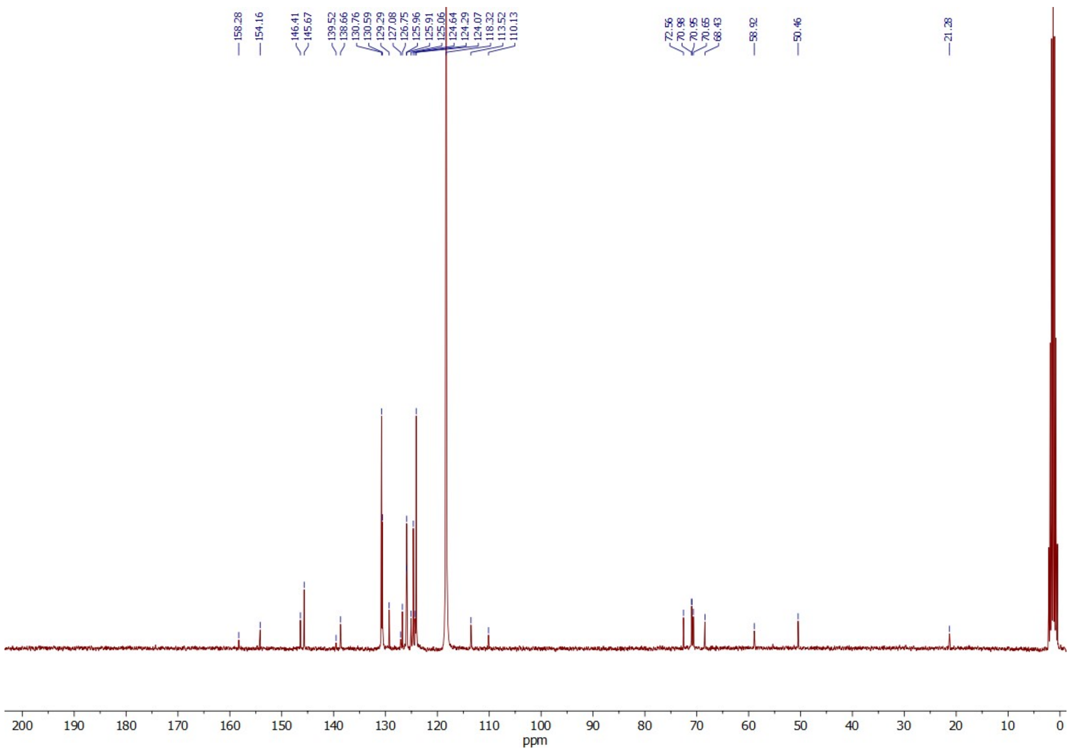

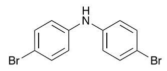

^{1}H NMR (600 MHz, $CDCl_3$)

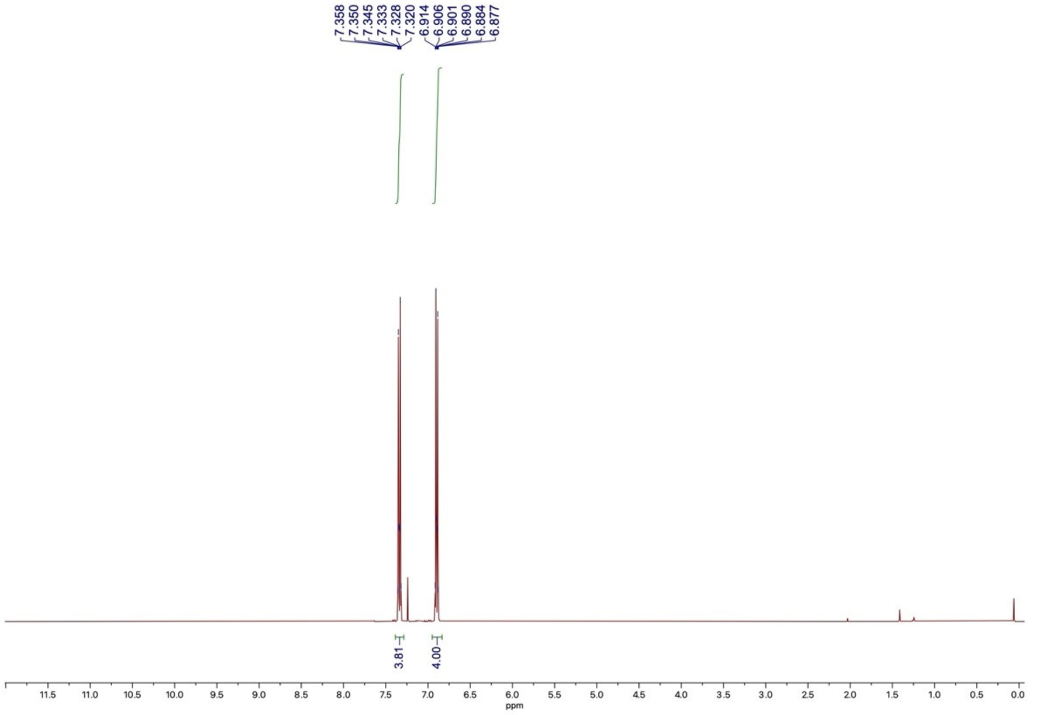

{^{1}H}^{13}C NMR (150 MHz, $CDCl_3$)

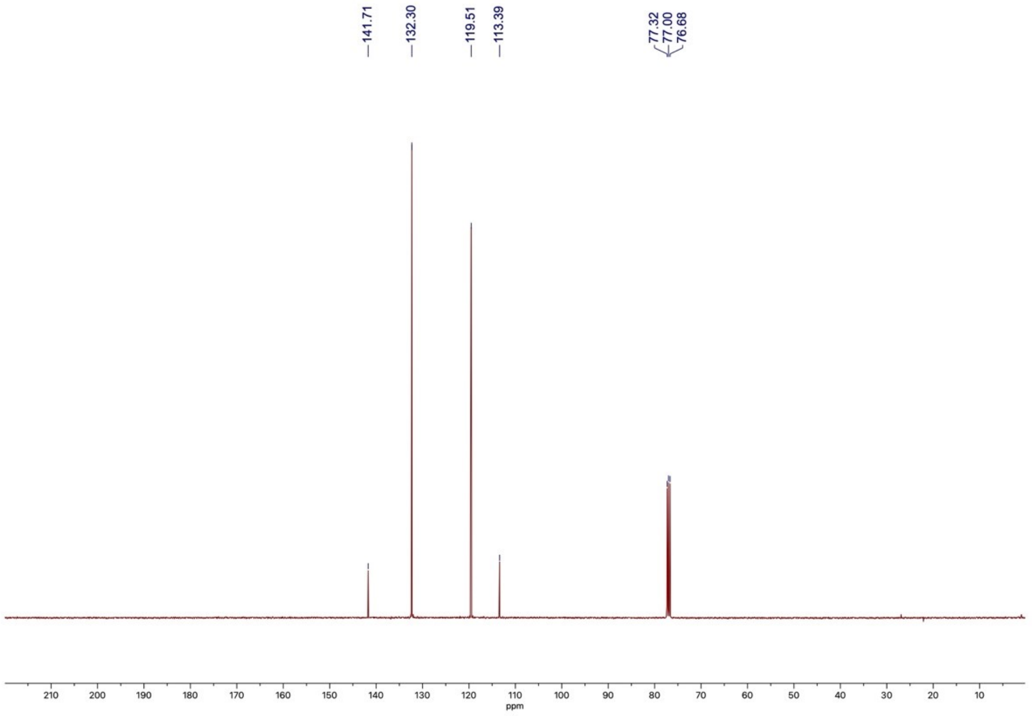

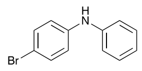

^{1}H NMR (600 MHz, $CDCl_3$)

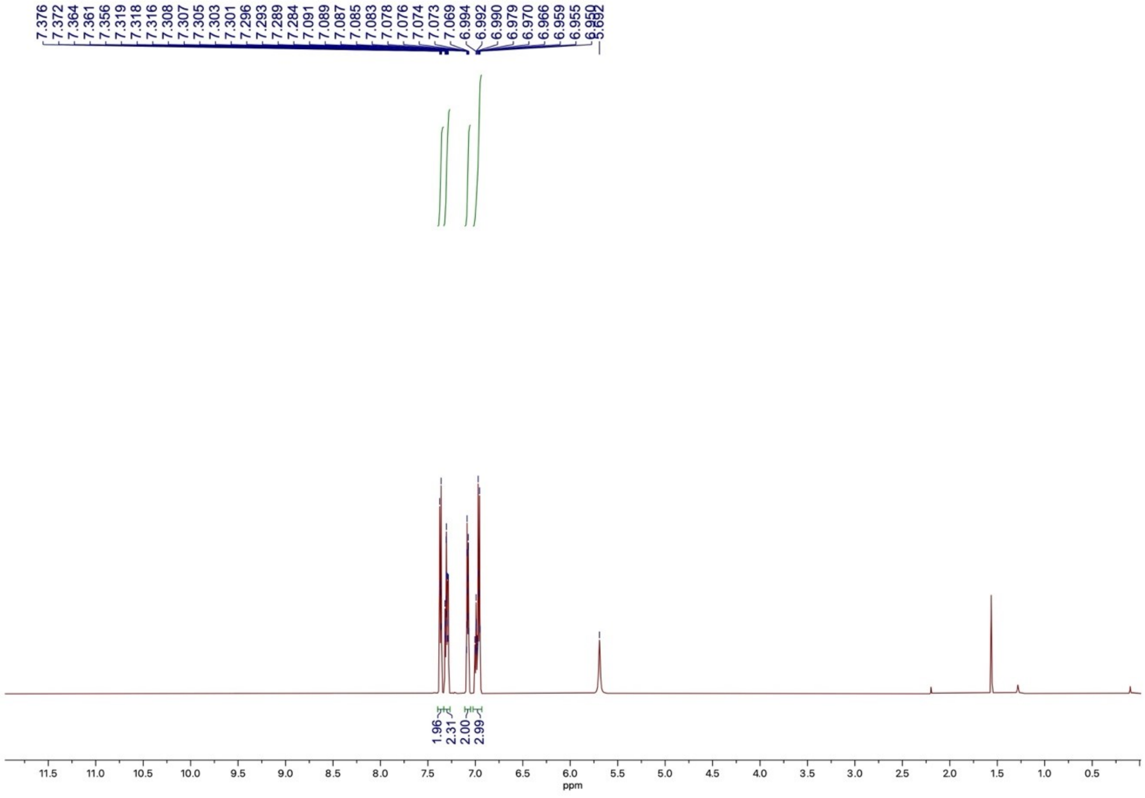

{^{1}H}^{13}C NMR (150 MHz, $CDCl_3$)

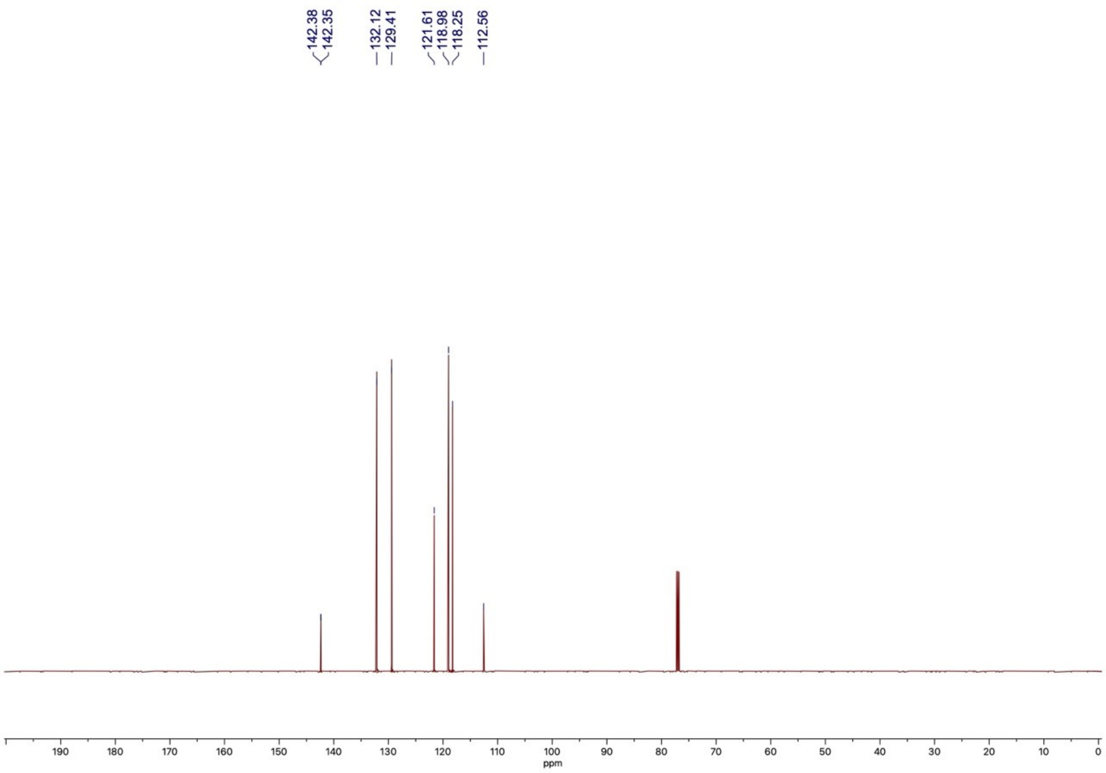

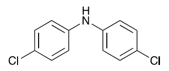

^{1}H NMR (600 MHz, $CDCl_3$)

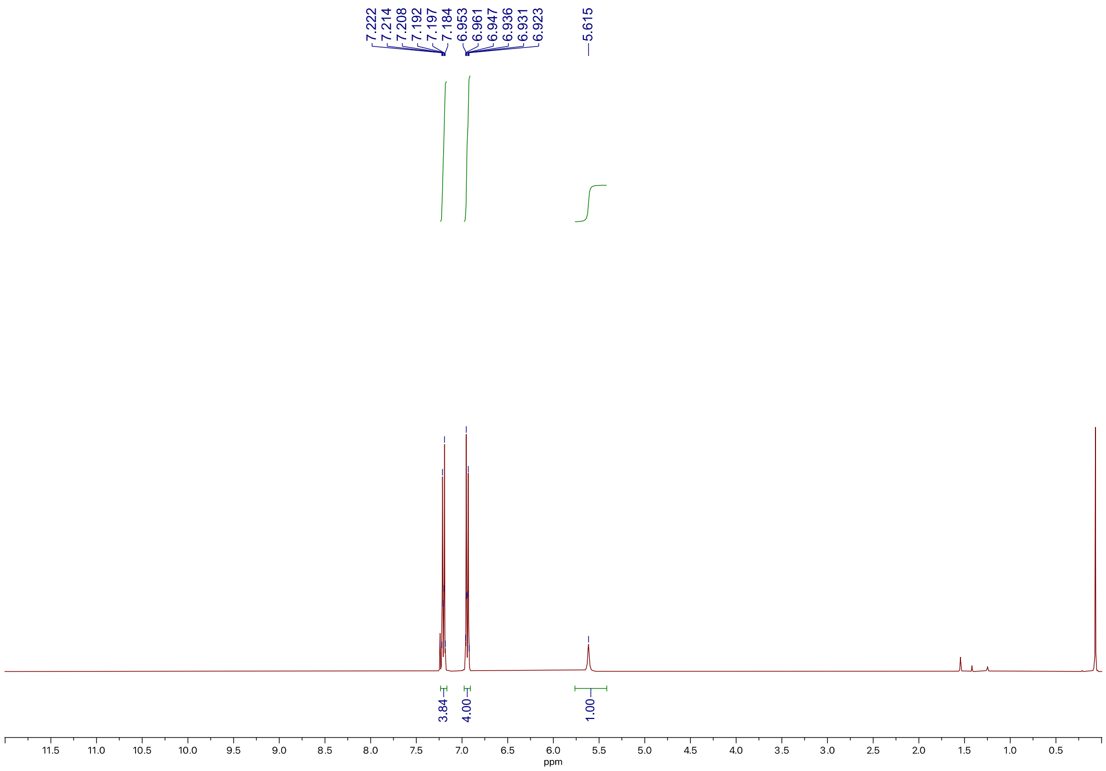

{^{1}H}^{13}C NMR (150 MHz, $CDCl_3$)

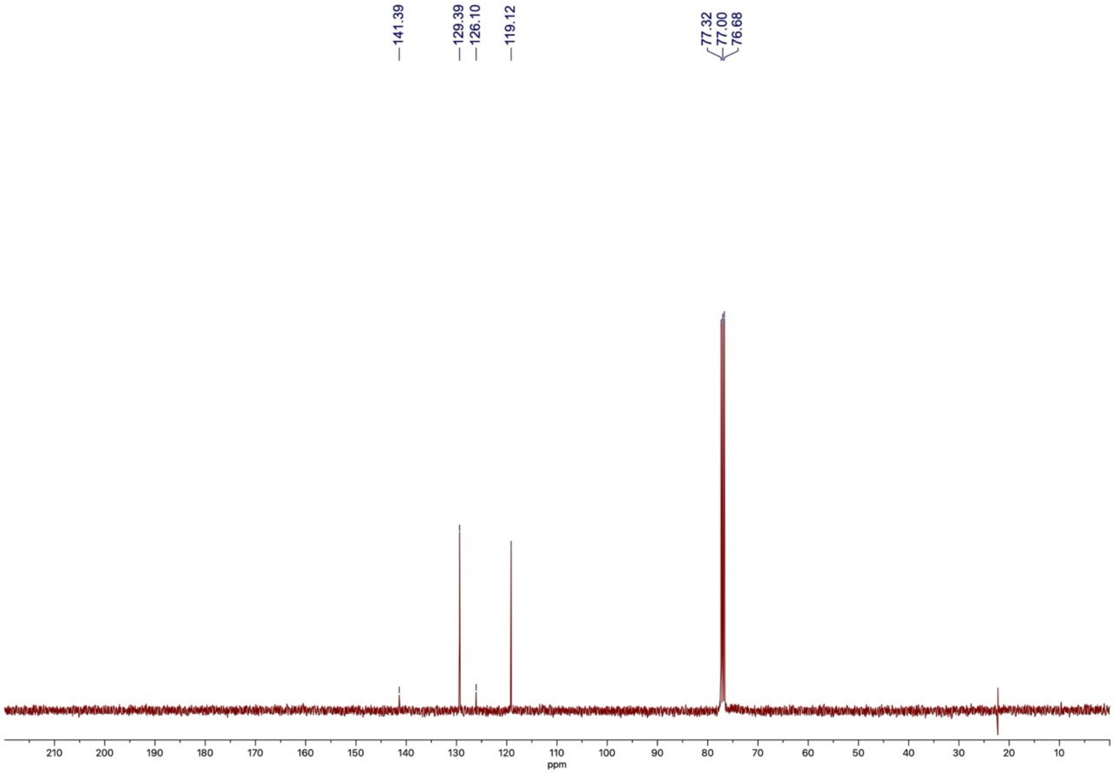

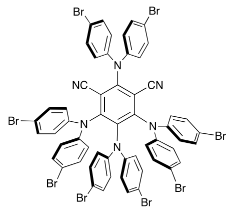

^{1}H NMR (600 MHz, CD_2Cl_2)

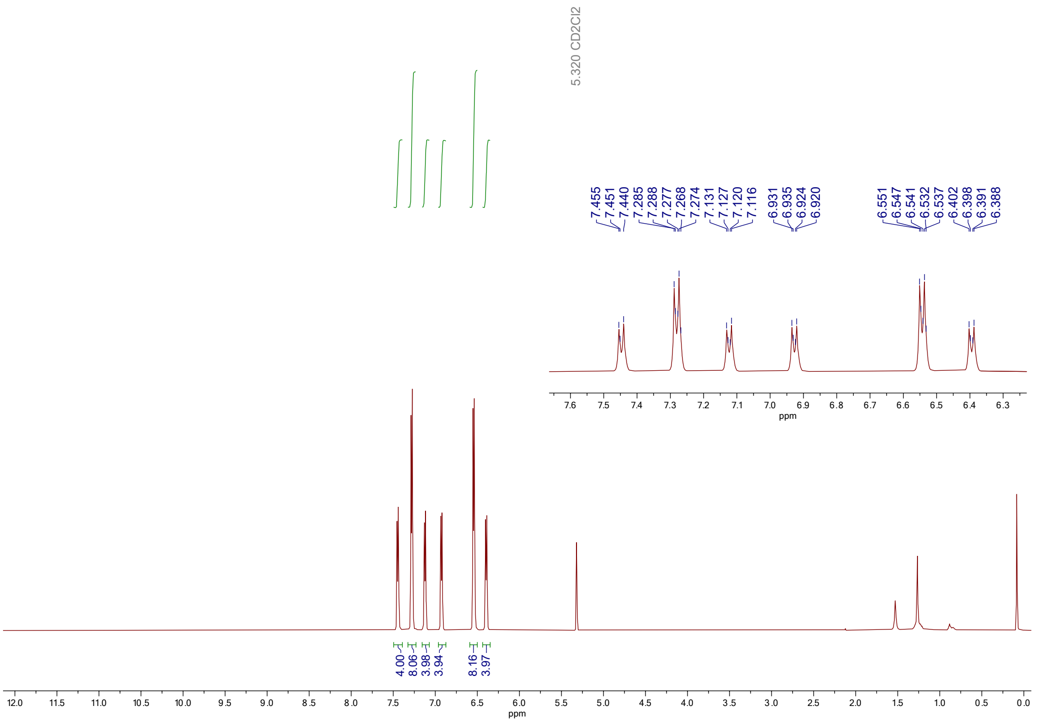

$\{^{1}H\}^{13}C$ NMR (150 MHz, CD_2Cl_2)

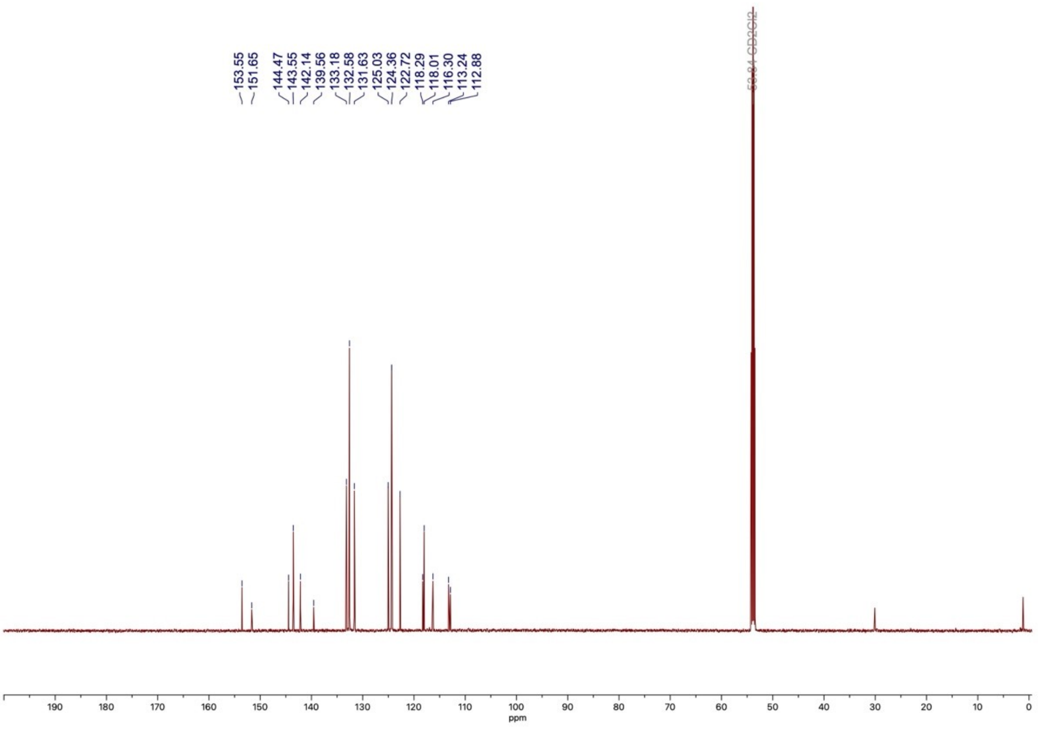

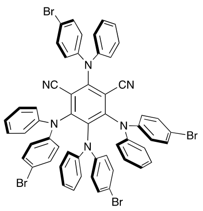

^{1}H NMR (600 MHz, $CDCl_3$)

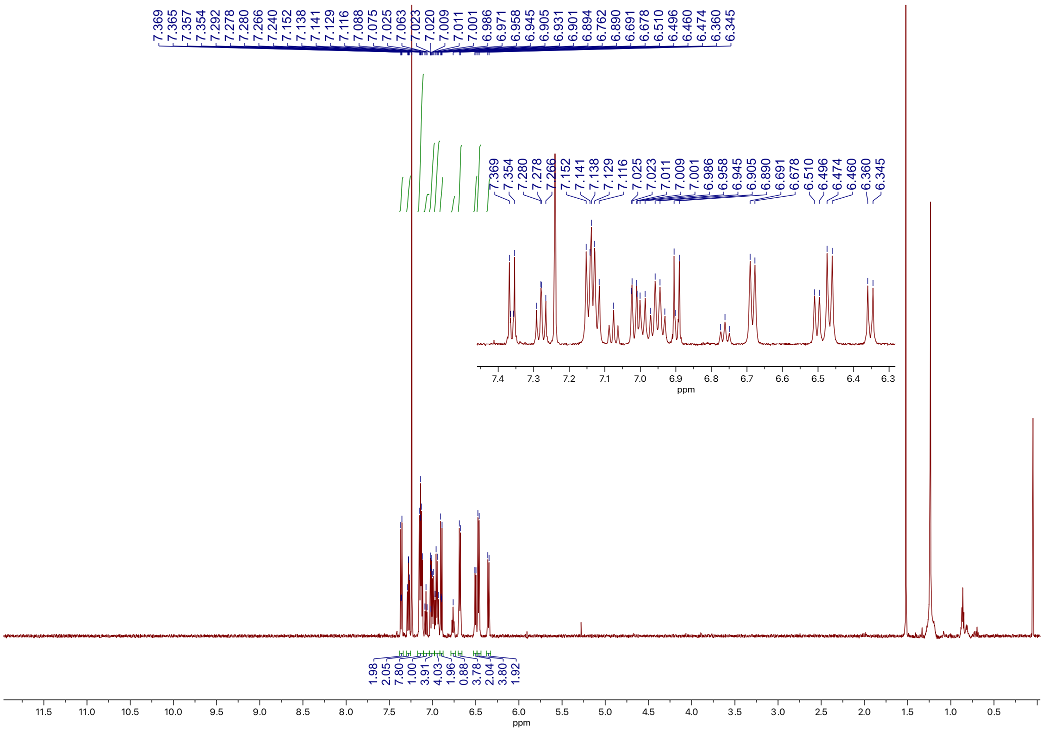

{^{1}H}^{13}C NMR (150 MHz, $CDCl_3$)

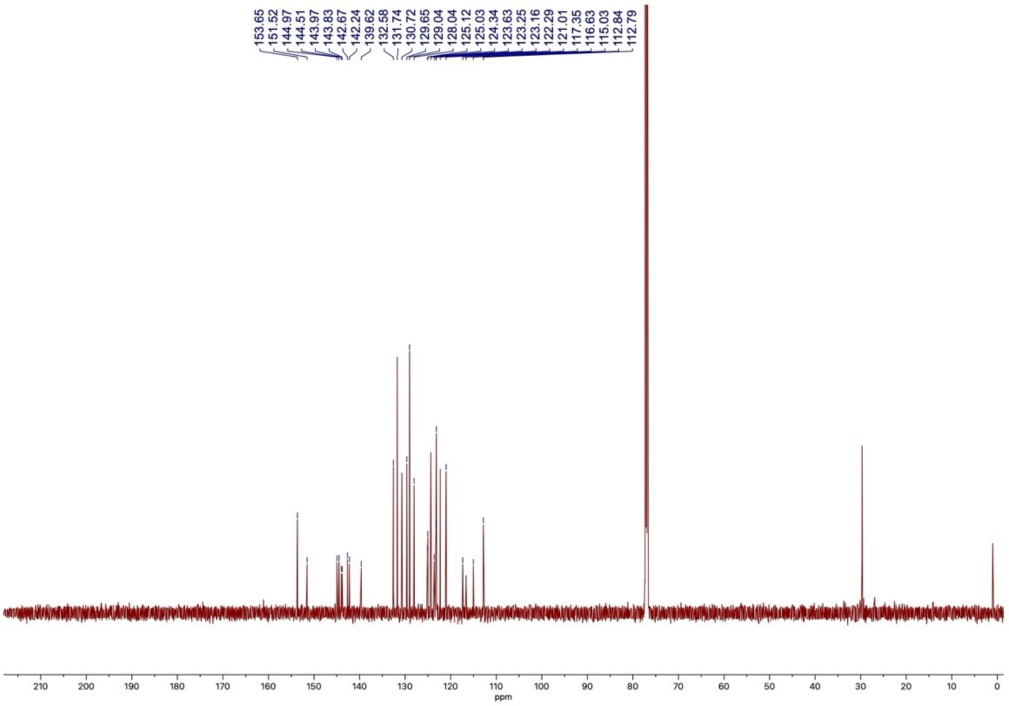

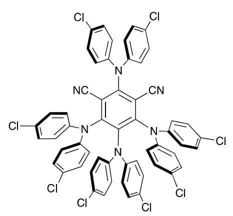

^{1}H NMR (600 MHz, $CDCl_3$)

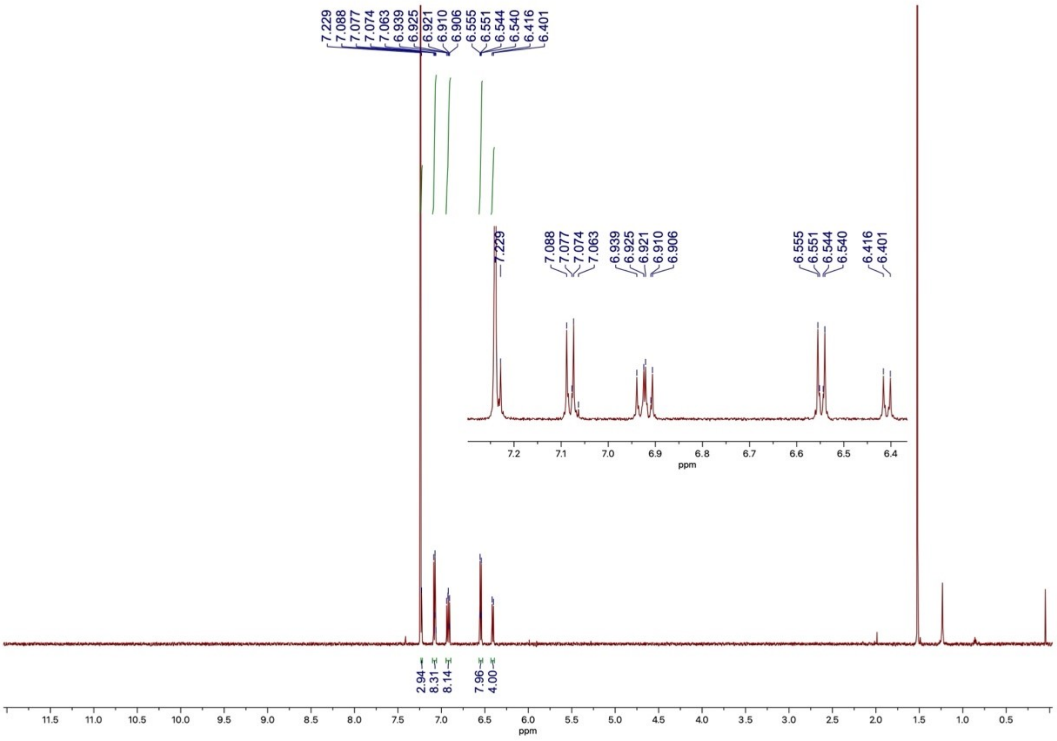

{^{1}H}^{13}C NMR (150 MHz, $CDCl_3$)

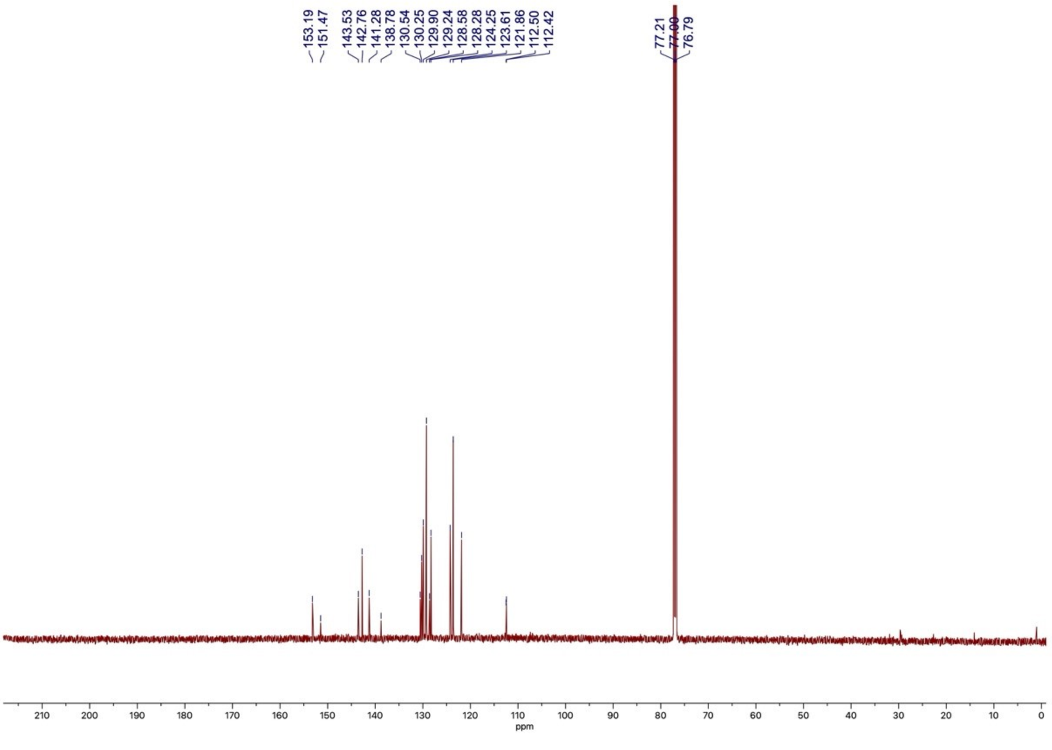

Experimental information

Photophysical characterization of acceptor-modified compounds

All samples were prepared in spectroscopic-grade Uvasol® acetonitrile and dichloromethane, that were used as received, with a $5 \cdot 10^{-6}$ M concentration.
Absorption spectra were recorded with a Varian Cary 100bio UV-VIS spectrophotometer in 10 mm quartz cuvettes filled with non-degassed solution of investigated compounds.
Emission and excitation spectra were recorded with a Photoluminescence Spectrometer (Edinburgh Instruments, FLS1000) equipped with a 450 W Xe lamp and an air-cooled single-photon counting photomultiplier (Hamamatsu R13456). The solutions of investigated compounds, contained in a 10 mm quartz cuvette, were bubbled with argon for 15 minutes prior measurements.
Absolute Photoluminescence Quantum Yields of solutions were obtained with a Hamamatsu Absolute PL quantum yield spectrometer C11347 Quantaurus QY using a quartz tube filled with non-degassed or Ar-purged solutions of investigated compounds.
Lifetimes were obtained on a FLS1000 in Ar-purged or non-degassed solutions.
Time-resolved lifetime measurements were acquired on the FLS1000 spectrometer equipped with a picosecond laser diode at 402.3 nm. Luminescence decays were recorded using the Time correlated single photon counting (TCSPC) for lifetime in the nanosecond range, or the Multi-channel scaling (MCS) technique for lifetime in the microsecond range. The frequency of the laser was changed according to the lifetime of each sample.
Decay curves were analyzed with the Fluoracle Software using the IRF convolution fitting (for lifetime in ns range) or the tail-fitting for longer lifetimes.

Photophysical characterization of donor-modified compounds

UV–vis absorption spectra were recorded with a PerkinElmer λ40 spectrophotometer using quartz cells with an optical path length of 10 mm. The emission spectra were recorded using an Edinburgh FLS920 spectrofluorometer equipped with a Hamamatsu R928 phototube or an Edinburgh FS5 spectrofluorometer. Lifetimes shorter than 20 µs were measured by the above-mentioned Edinburgh FLS920 spectrofluorometer equipped with a TCC900 card for data acquisition in time-correlated single-photon counting experiments (0.5 ns time resolution) with a 405 nm pulsed laser. Lifetimes longer than 20 µs were measured by the above-mentioned Edinburgh FS5 spectrofluorometer.
The emission quantum yield of the ligand was determined through the method of Demas and Crosby using quinine sulfate in 0.5 M H_2SO_4 (Φ_{em} = 0.55) as a standard.[8] Deaerated solutions were obtained by Ar bubbling (15 minutes) in sealed quartz cuvettes. The estimated experimental errors are 2 nm on the band maximum, 5% on the molar absorption coefficient and luminescence lifetime and 10% on the emission quantum yield in solution.

Electrochemical characterization

The electrochemical characterization was performed using a SP-300 potentiostat (BioLogic Science Instrument, France) equipped with a conventional three-electrode system housed in a glass cell. The three-electrode system contained a glassy carbon working electrode (1 mm diameter), platinum wire counter electrode, and a silver wire quasi-reference electrode. Cyclic voltammograms were performed at a scan rate of 100 mV/s in anhydrous acetonitrile solutions containing $1\cdot10^{-3}$ M luminophore and 0.1 M $TBAPF_6$ as the supporting electrolyte. Before use, the glassy carbon electrode was polished using 0.3 and 0.05 µm alumina powder, sonicated in water, then rinsed with acetone, and eventually dried under Argon flux before use. Prior to analysis, solutions were deoxygenated by purging Argon until the current attributed to the oxygen reduction was negligible. The potentials are referenced to the SCE reference electrode by applying a -0.096 V correction factor to the potentials referenced to the $Me_{10}Fc^+/Me_{10}Fc$ redox couple.[9] All electrochemical experiments were carried out at room temperature.

Electrochemiluminescence characterization

The ECL emission is triggered by chronoamperometric pulses/cyclic voltammetry controlled by a SP-300 potentiostat (BioLogic Science Instrument, France). ECL measurements on purely organic molecules were conducted using a three-electrode system in anhydrous acetonitrile solutions containing $0.5\cdot10^{-3}$ M luminophore, 0.1 M $TBAPF_6$ as the supporting electrolyte, and 10 mM BPO when needed. On the other hand, ECL measurements on water-soluble emitters were carried out using the same three-electrode system in aqueous solutions containing $0.5\cdot10^{-3}$ M luminophore, 0.3 M PB as the supporting electrolyte, and 180 mM TPrA. A platinum side-oriented disk (2 mm diameter) sealed in glass was employed as the working electrode. The counter electrode was a platinum spiral, while a quasi-reference silver wire was used as the reference electrode. The ECL measurements in aqueous environment were carried out using a Ag/AgCl reference electrode. This three-electrode system was placed in a one-compartment airtight cell, equipped with high-vacuum O-rings and glass stopcocks. The cell, housed in a dark box, was carefully placed as close as possible the Hamamatsu R928 photomultiplier tube (PMT; Hamamatsu Photonics K.K., Japan), which was positioned in front of the working electrode. The ECL signal was collected by the PMT and amplified with a Keithley Model 6485 Picoammeter (Keithley Instruments Inc., United States).
The ECL quantum efficiency (Φ_{ECL}) was determined in annihilation conditions. This approach involved applying a square-wave voltage (1.25 Hz) in the diffusion-limited region (i.e., 100 mV past peak current) in anhydrous acetonitrile containing 0.1 M $TBAPF_6$.

The Φ_{ECL} was then calculated relatively to the $[Ru(bpy)_3](PF_6)_2$ standard exploiting the following equation (Eq. S1):

$$\Phi_{ECL,x} = \Phi_{ECL,st} \cdot \left(\frac{ECL_x}{ECL_{st}}\right) \cdot \left(\frac{Q_{st}}{Q_x}\right) \qquad \text{(S1)}$$

Where $\Phi_{ECL,st}$ is the ECL efficiency of the standard under the same conditions ($[Ru(bpy)_3](PF_6)_2$ Φ_{ECL} = 0.05)[10], ECL_x and ECL_{st} are the integrated values of the first ECL emission peak of the given

compound and the standard dye, respectively. The integrated ECL value is normalized over the quantum efficiency of spectral response of the PMT at the wavelength of maximum ECL emission. Q_x and Q_{st} are the integrated values of the faradaic current involved in the generation of the first ECL peak for the studied molecule and the standard luminophore, respectively.
The annihilation ECL signal is normalized over the quantum efficiency of the spectral response of the PMT at the wavelength of maximum ECL emission.
To record ECL spectra, the ECL signal is generated by applying a cathodic potential in the diffusion-controlled region.

Computational details

Quantum-chemical calculations were carried out using Gaussian 16 (A.03) suite of programs.[11] Ground state geometries of the investigated TADF luminophores were determined with the B3LYP-D3/6-31G(d) level of theory. Excitation energies of the lowest singlet and triplet excited states were determined at time-dependent DFT (TD-DFT) level with the Tamm Dancoff approximation (TDA). Single point calculations at the ground state optimized geometries were therefore carried out at TDA-M062X/6-31+G(d) level of theory including solvent effects (toluene) using the polarizable continuum model (PCM).[12]
Frontier orbital energies (E_{HOMO} and E_{LUMO}), collected in Table S1 for acceptor-modified molecules and in Table S3 for donor-modified molecules, were obtained at the optimized ground state geometries with the B3LYP-D3/6-31G(d) level of theory. The remaining data collected in Table S1 and Table S3 (vertical excitation energies of the lowest singlet and triplet states, $E(S_1)$, $E(T_1)$, the oscillator strength of S_1, f, and the singlet-triplet energy difference $\Delta E_{ST}=(E(S_1)-E(T_1))$, were obtained at TDA-M06-2X/6-31+G(d) level including solvent effects with the PCM (toluene).
To assess the effect of the functional on the optimized geometry, **3DPA2FBN** ground state was determined also at M062X/6-31G(d) level. Very minor changes are determined for the ΔE_{ST}, changing from 0.39 eV to 0.38 eV. To assess the effect of conformation, two different conformers were considered for **3DPA2ImBN**, the second derived from the crystal structure. The optimized structure resulted less stable by 0.48 kcal/mol, with a ΔE_{ST} of 0.32 eV compared to 0.36 of the more stable conformer (Table S1).

Photophysics of acceptor-modified compounds

Absorption spectra

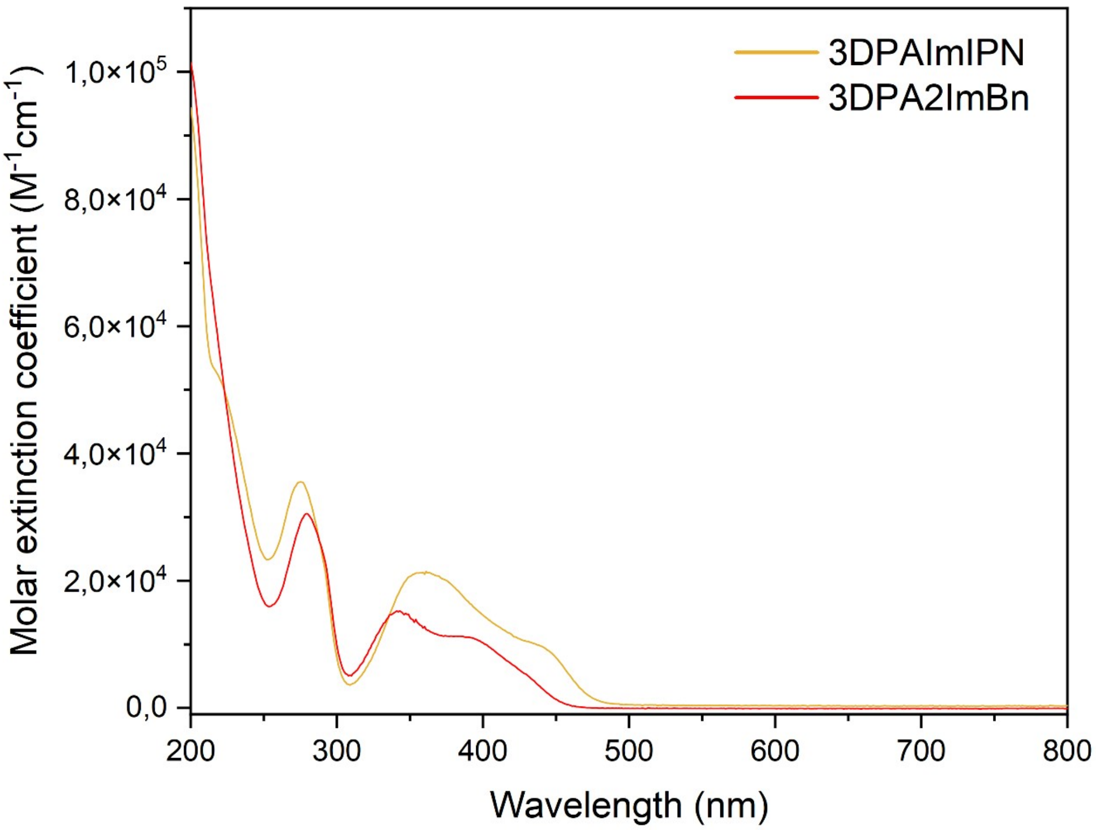

Figure S1. UV-vis spectra of $5 \cdot 10^{-6}$ M solutions of 3DPAImIPN (yellow line), and 3DPA2ImBn (red line) in non-degassed acetonitrile.

Emission spectra

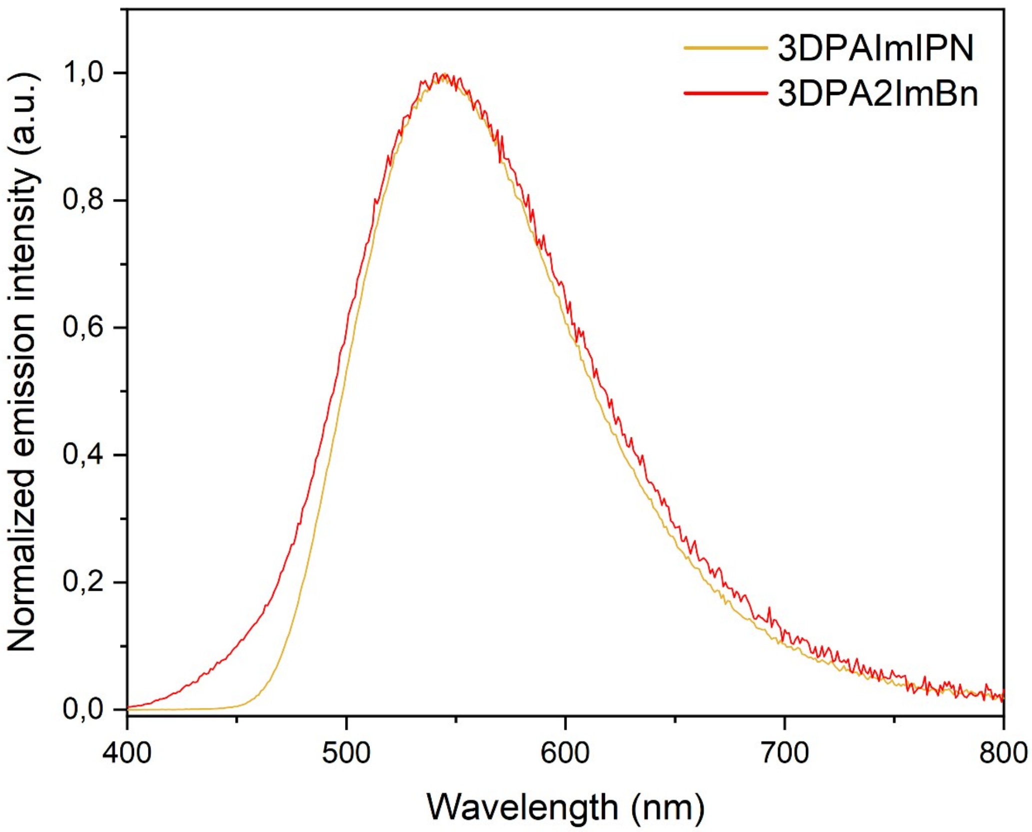

Figure S2. Normalized photoluminescence spectra of $5{\cdot}10^{-6}$ M solutions of 3DPAImIPN (yellow line), and 3DPA2ImBn (red line) in non-degassed acetonitrile.

Excitation spectra

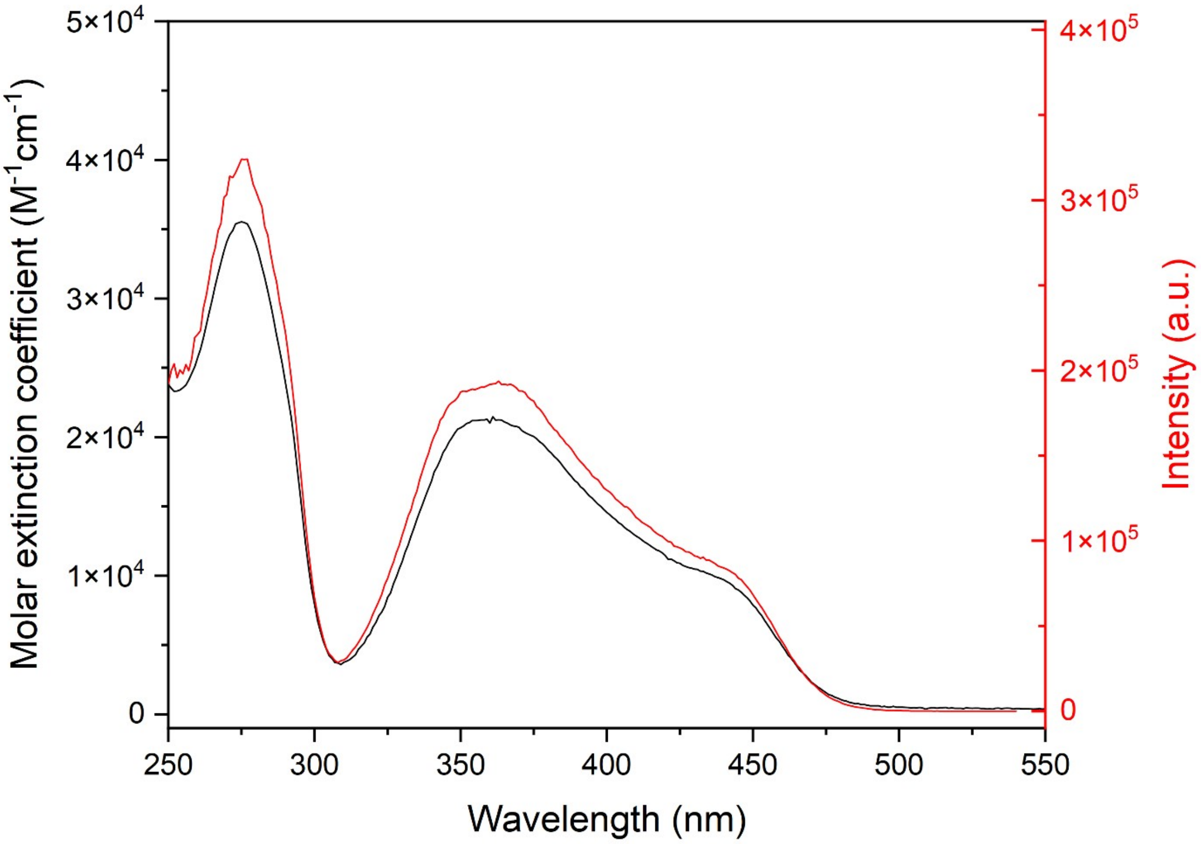

Figure S3. Comparison between the absorption spectrum (black line) in non-degassed acetonitrile and the excitation spectrum (red line) in degassed acetonitrile of a $5 \cdot 10^{-6}$ M solution of 3DPAImIPN. The λ_{em} was set at 550 nm.

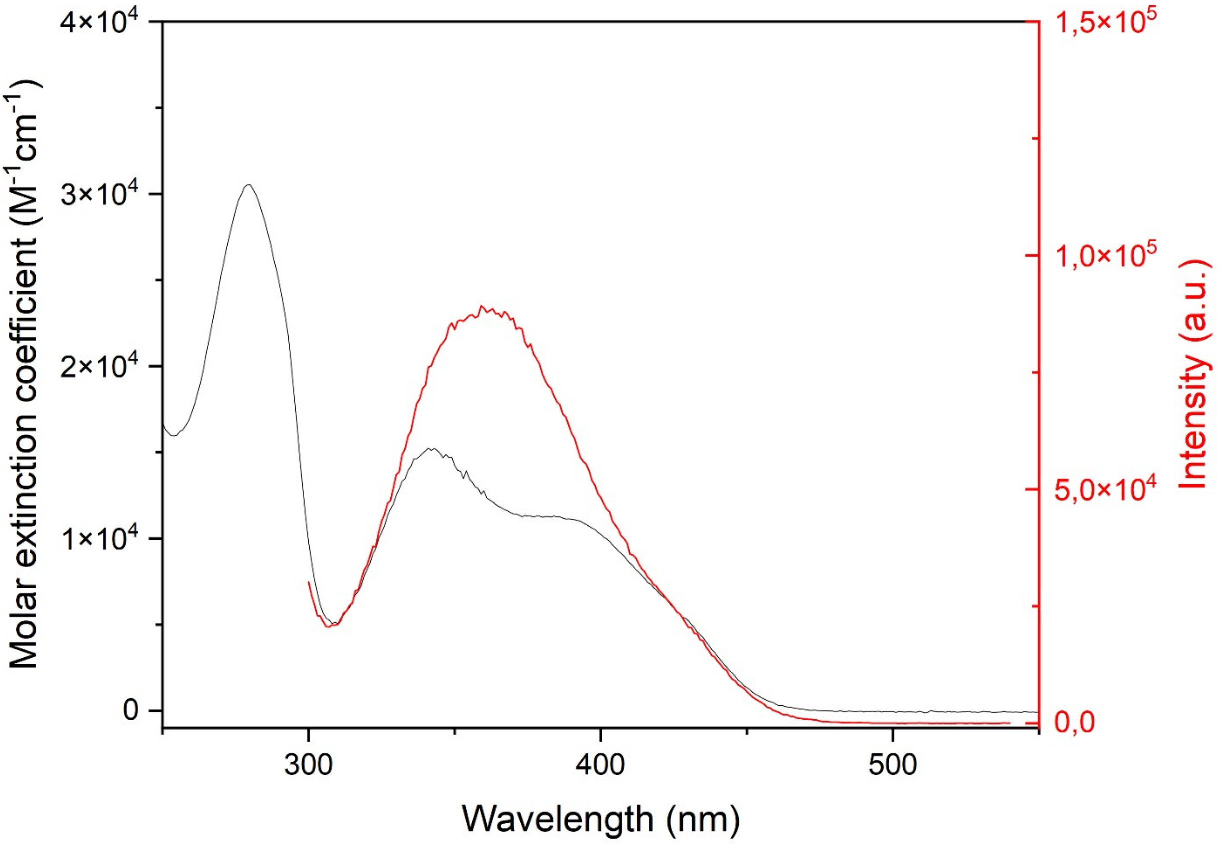

Figure S4. Comparison between the absorption spectrum (black line) in non-degassed acetonitrile and the excitation spectrum (red line) in degassed acetonitrile of a $5 \cdot 10^{-6}$ M solution of 3DPA2ImBn. The λ_{em} was set at 550 nm.

Computed DFT data for acceptor-modified molecules

Table S1. Frontier orbital energies (E_{HOMO} and E_{LUMO}) from B3LYP-D3/6-31G(d) calculations at the optimized groud state geometry; vertical excitation energies of the lowest singlet and triplet states, $E(S_1)$, $E(T_1)$, oscillator strength of S_1, f, and the singlet-triplet energy difference $\Delta E_{ST}=(E(S_1)- E(T_1))$, from TDA-M06-2X/6-31+G(d) in toluene (solvent described with PCM).

	E_{HOMO} /Hartree(eV)	E_{LUMO} /Hartree(eV)	$E(S_1)$/eV	$f(S_1)$	$E(T_1)$/eV	ΔE_{ST}/eV
**3DPA2FBN**	-0.193(-5.25)	-0.063(-1.71)	3.498	0.2284	3.108	0.390
**3DPA2ImBN**	-0.199(-5.42)	-0.072(-1.96)	3.396	0.1308	3.035	0.361
**3DPAFIPN**	-0.195(-5.31)	-0.076(-2.07)	3.249	0.3285	2.912	0.337
**3DPAImIPN**	-0.201(-5.47)	-0.080(-2.18)	3.189	0.2176	2.886	0.303

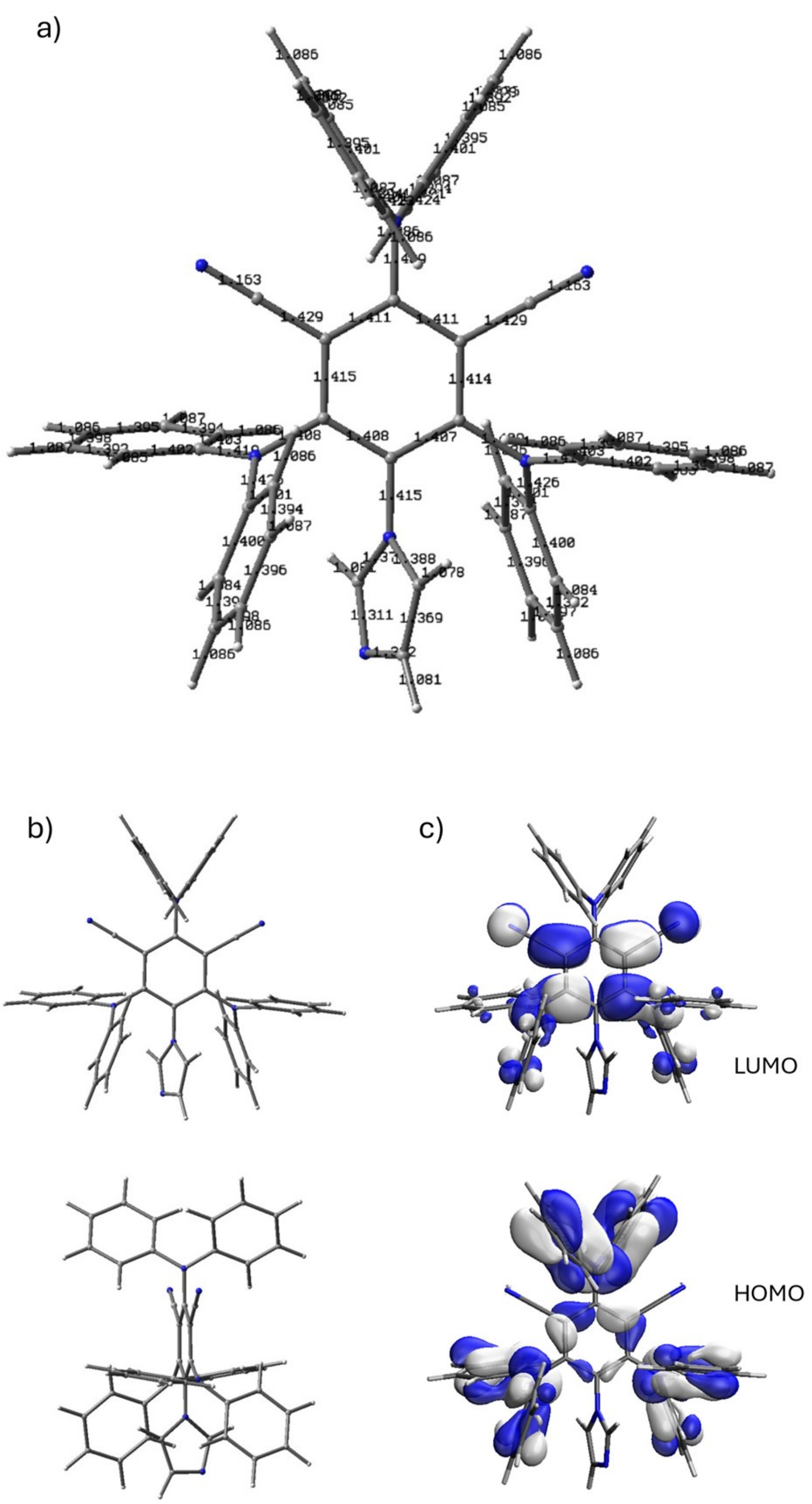

Figure S5. 3DPAImIPN: a) Ground state optimized bond lengths; b) front and side views of the ground state optimized geometry and c) frontier molecular orbitals. From B3LYP-D3/6-31G(d) calculations.

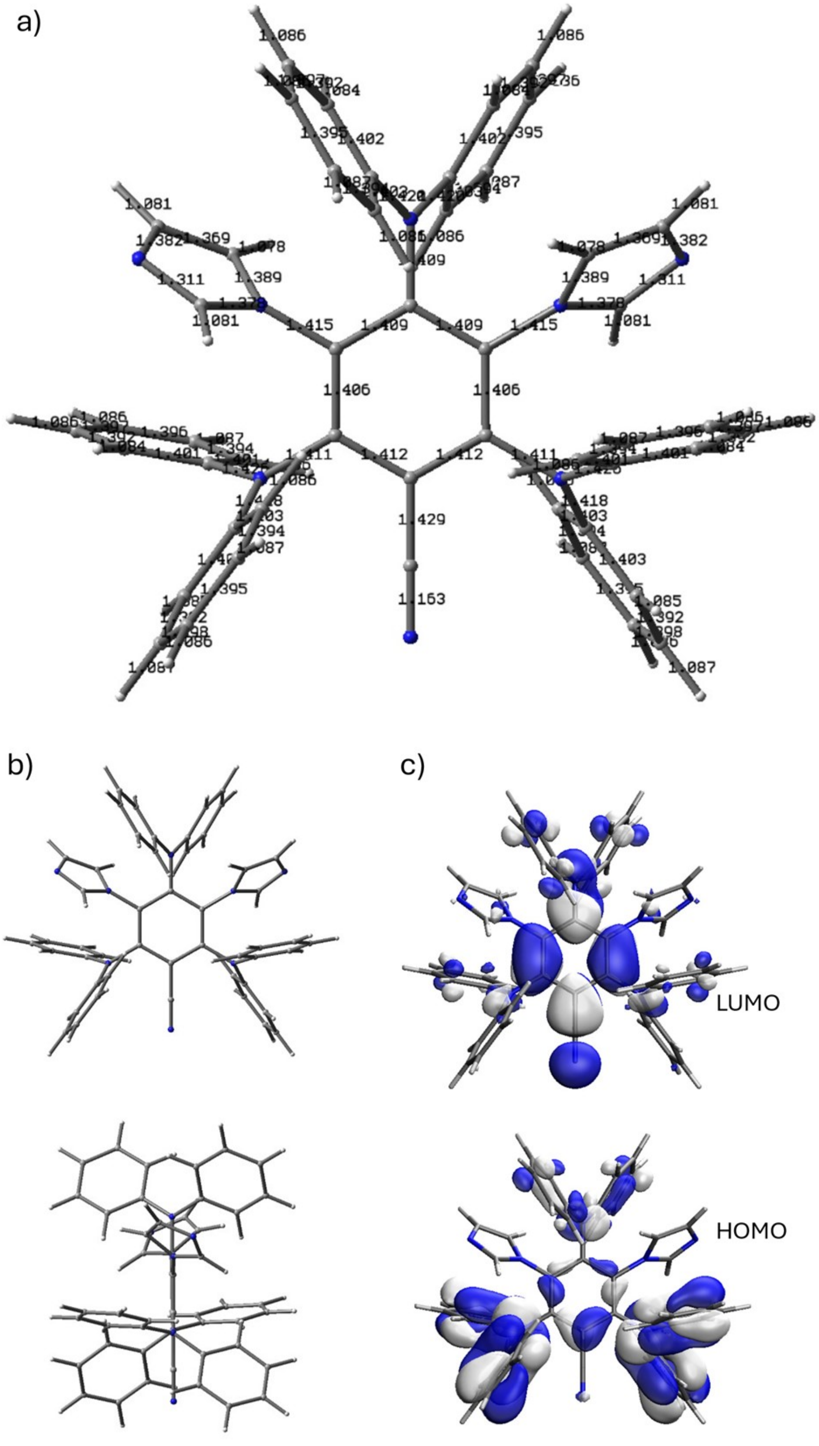

Figure S6. 3DPA2ImBN: a) Ground state optimized bond lengths; b) front and side views of the ground state optimized geometry and c) frontier molecular orbitals. From B3LYP-D3/6-31G(d) calculations.

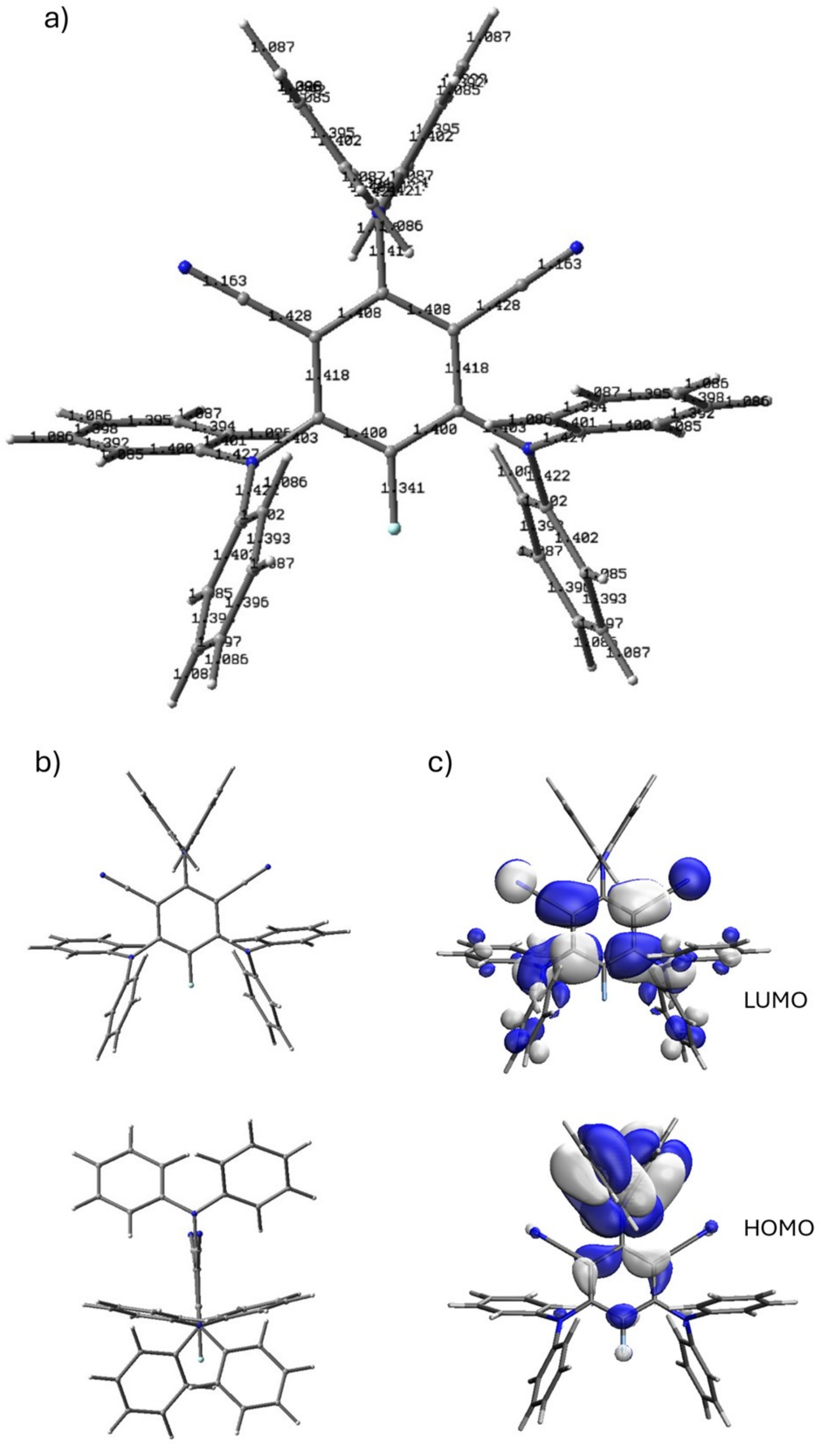

Figure S7. 3DPAFIPN: a) Ground state optimized bond lengths; b) front and side views of the ground state optimized geometry and c) frontier molecular orbitals. From B3LYP-D3/6-31G(d) calculations.

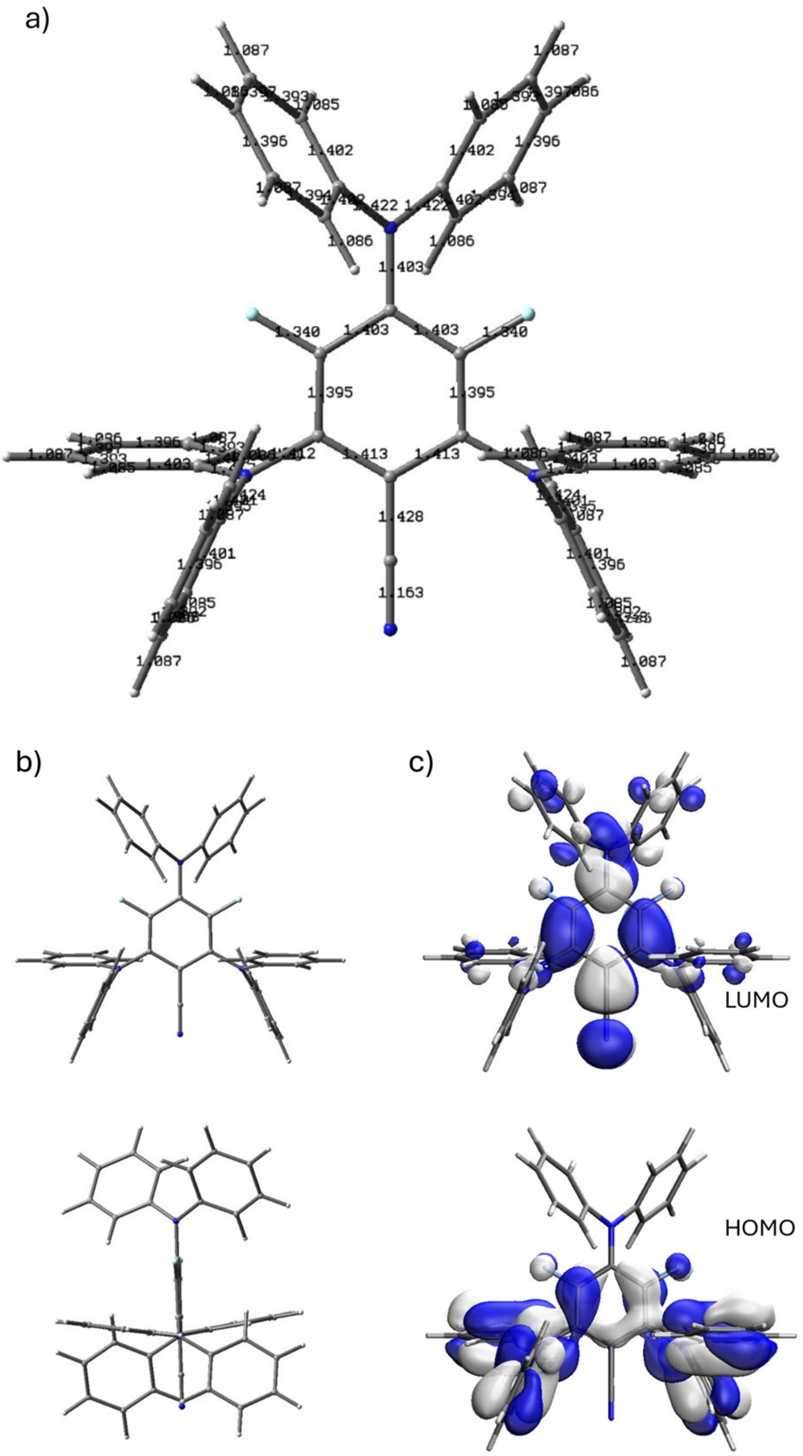

Figure S8. 3DPA2FBN: a) Ground state optimized bond lengths; b) front and side views of the ground state optimized geometry and c) frontier molecular orbitals. From B3LYP-D3/6-31G(d) calculations.

XRD Analysis – Structural characterization of 3DPA2ImBN

X-Ray diffraction data were collected at the XRD2 beamline of the Elettra Synchrotron, Trieste (Italy).[13] The crystals were dipped in NHV oil (Jena Bioscience, Jena, Germany) and mounted on the goniometer head with kapton loops (MiTeGen, Ithaca, USA). Complete datasets were collected by the rotation method at 100 K (nitrogen stream supplied through an Oxford Cryostream 700). Data were acquired using monochromatic wavelength of 0.620 Å on a Pilatus 6M hybrid-pixel area detector (DECTRIS Ltd., Baden-Daettwil, Switzerland). Diffraction data were indexed, integrated and scaled using XDS.[14] Structures were solved by the dual space algorithm implemented in the SHELXT program.[15] Fourier analysis and refinement were performed by the full-matrix least-squares methods based on F^2 implemented in SHELXL (Version 2019/3).[16] The Coot program was used for modeling.[17] Anisotropic thermal motion refinement was used for all atoms. Geometry parameters restrains (SAME and SADI) were applied to disordered fragments. Hydrogen atoms were included at calculated positions with isotropic $U_{factor} = 1.2 \bullet U_{eq}$ (U_{eq} being the equivalent isotropic thermal factor of the bonded non hydrogen atom). Crystals showed solvent molecules trapped into crystal voids. Electron density contribution that couldn't be modeled in **3DPA2ImBN** was removed with Platon SQUEEZE[18] routine. Estimated contributions of these regions (72 electrons in 204 Å^3) are included in the reported crystal properties (Table S2) and correspond to 2 additional disordered dichloromethane molecules per unit cell. Pictures were prepared using Ortep-3[19] software.

3DPA2ImBN crystallizes in a monoclinic centrosymmetric space group with one full molecule in the asymmetric unit (ASU – Figure S9). Intramolecular π•••π make the two imidazole rings in **3DPA2ImBN** not equivalent (i.e. only one is locked in a specific orientation) and force them to stack with adjacent phenyl rings (shortest $d_{\pi \bullet\bullet\bullet \pi}$ = 3.633(2) Å). Crystal packing show layers of molecules parallel to *ab* cell face where nitrile group of one molecule stays almost perpendicular to core phenyl ring of a neighbour molecule ($d_{CN \bullet\bullet\bullet \pi}$ = 4.663(1) Å and $\Lambda_{CN \bullet\bullet\bullet \pi}$ = 82.4(1)°). Layers are linked by intermolecular CH•••π contacts (shortest one shows $d_{CH \bullet\bullet\bullet \pi}$ = 3.584(1) Å and $\Lambda_{CH \bullet\bullet\bullet \pi}$ = 66.0(1)°).

Table S2. Crystallographic data and refinement details.

	**3DPA2ImBN**
CCDC Number	2359842
Chemical Formula	$C_{49}H_{36}N_8 \cdot {}^{1}/_{2}CH_2Cl_2$
Formula weight	779.32 g/mol
Temperature	100(2) K
Wavelength	0.620 Å
Crystal system	Monoclinic
Space Group	$P\ 2_1/c$
Unit cell dimensions	a = 13.982(3) Å
	b = 12.019(2) Å
	c = 23.870(5) Å
	α = 90°
	β = 97.56(3)°
	γ = 90°
Volume	3976.4(14) $Å^3$
Z	4
Density (calculated)	1.302 $g{\cdot}cm^{-3}$
Absorption coefficient	0.103 mm^{-1}
F(000)	1628
Theta range for data collection	1.3° to 31.1°
Index ranges	-23 ≤ h ≤ 23,
	-19 ≤ k ≤ 19,
	-37 ≤ l ≤ 37
Resolution	0.60 Å
Reflections collected	92549
Independent reflections	17263, 15309 data with I>2σ(I)
Data multiplicity (max resltn)	4.75 (2.79)
I/σ(I) (max resltn)	24.67 (11.31)
R_{merge} (max resltn)	0.0336 (0.0790)
Data completeness (max resltn)	89.9% (72.9%)
Refinement method	Full-matrix least-squares on F^2
Data / restraints / parameters	17263 / 21 / 560
Goodness-of-fit on F^2	1.018
Δ/σ_{max}	0.004
Final R indices [I>2[illegible](I)]	R_1 = 0.0497, wR_2 = 0.1385
R indices (all data)	R_1 = 0.0540, wR_2 = 0.1428
Largest diff. peak and hole	0.538 and -0.583 $eÅ^{-3}$
R.M.S. deviation from mean	0.063 $eÅ^{-3}$

$R_1 = \Sigma\,||Fo|-|Fc||\,/\,\Sigma\,|Fo|$, $wR_2 = \{\Sigma[w(Fo^2 - Fc^2)^2] / \Sigma[w(Fo^2)^2]\}^{½}$

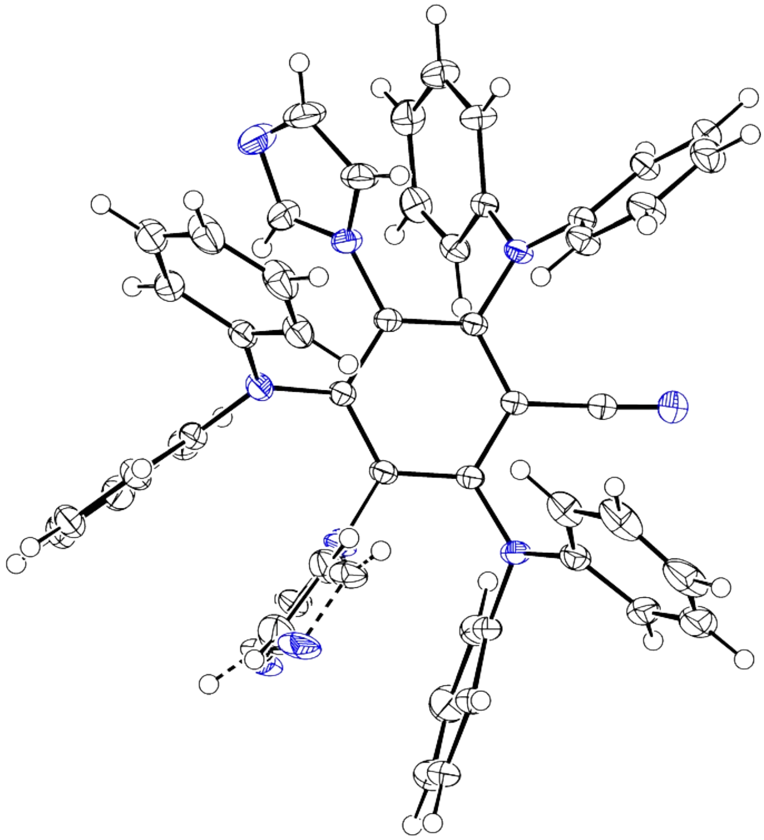

Figure S9. Ellipsoids representation of **3DPA2ImBN** asymmetric unit (A.S.U. - 50% probability).

Electrochemistry of acceptor-modified compounds

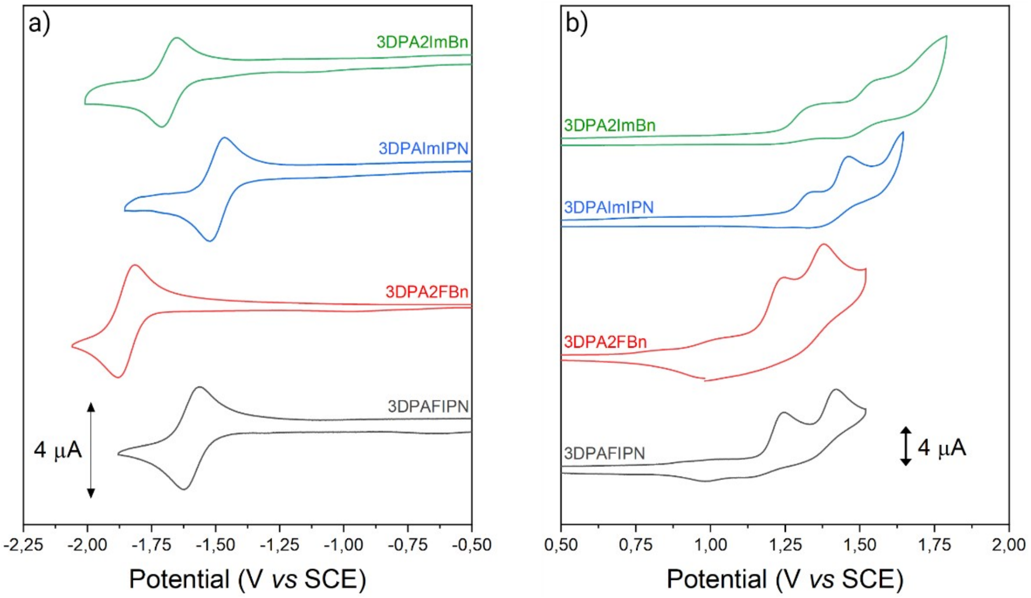

Figure S10. Cyclic voltammograms of the acceptor-modified molecules, highlighting the (a) cathodic and the (b) anodic regions. Measurements were performed in degassed MeCN solutions containing 1 mM luminophore and 0.1 M $TBAPF_6$ as the supporting electrolyte: 3DPAFIPN (grey line), 3DPA2FBn (red line), 3DPAImIPN (blue line), and 3DPA2ImBn (green line). Anodic and cathodic voltammograms of the same compound belong to the same scan. The $Me_{10}Fc^+/Me_{10}Fc$ redox couple was used as internal standard. All the cyclic voltammetries were performed at a scan rate of 0.1 V/s.

Electrochemiluminescence of acceptor-modified compounds

Annihilation ECL

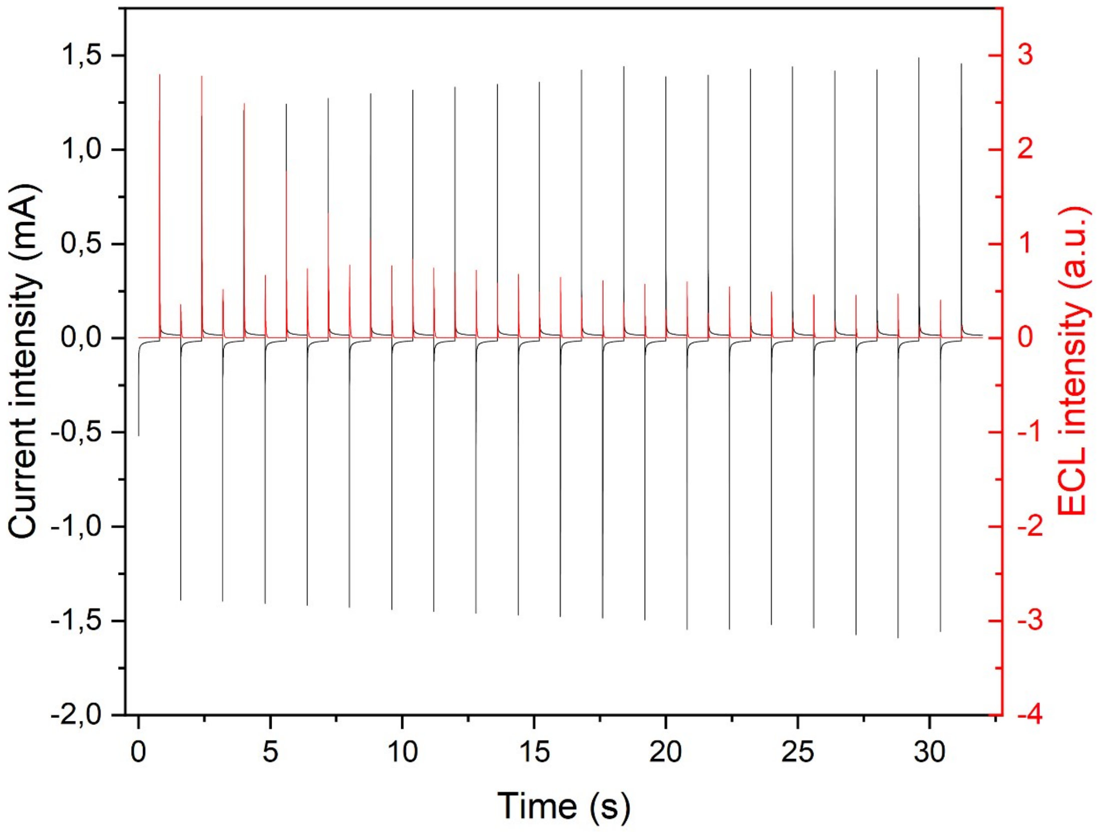

Figure S11. 20 annihilation cycles reporting current (black line) and ECL intensity (red line) of 3DPAFIPN (0.5mM) in AcN/TBAH 0.1 M collected versus time. The annihilation was performed by applying a cathodic potential of -1.72 V *vs* SCE for 0.8 s and then an anodic potential of 1.34 V *vs* SCE for 0.8 s. The measurement was carried out using a Pt disc working electrode (2 mm diameter), a Ag wire quasi-reference electrode, and a Pt wire counter electrode. PMT bias of 750 V; 000.0 µA amplification.

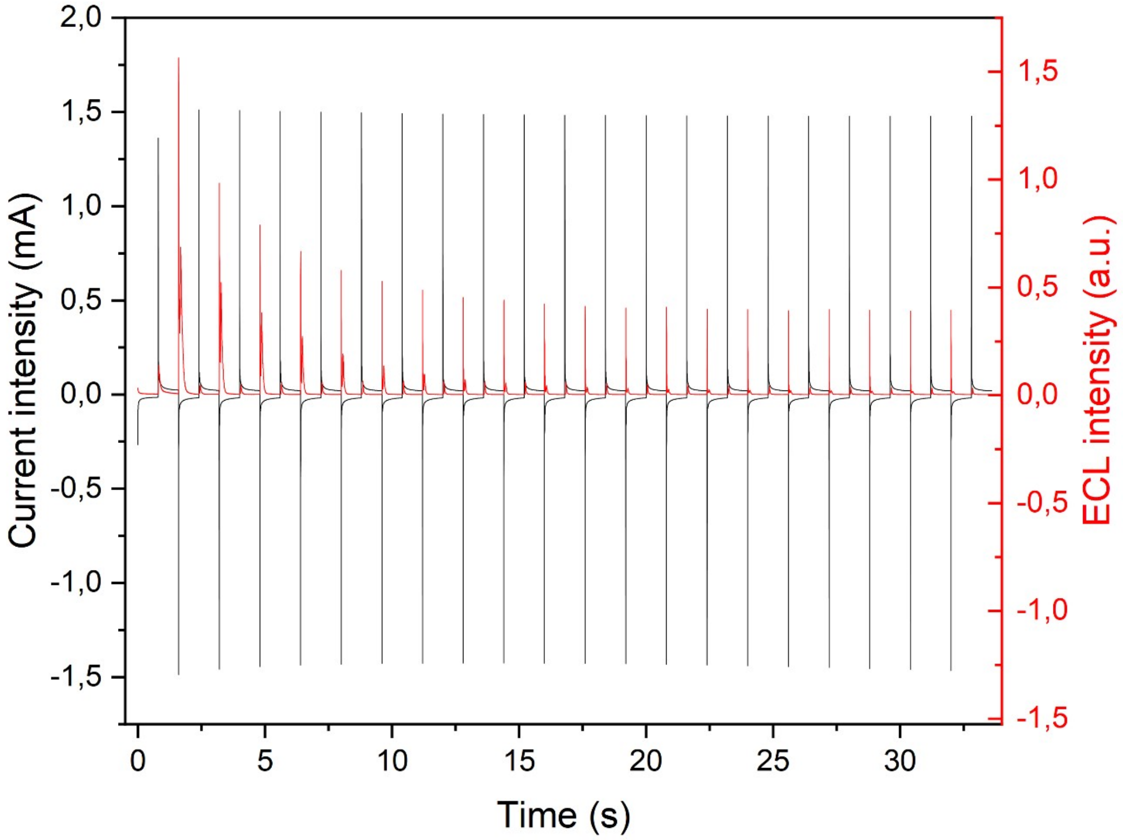

Figure S12. 21 annihilation cycles reporting current (black line) and ECL intensity (red line) of 3DPA2FBn (0.5mM) in AcN/TBAH 0.1 M collected versus time. The annihilation was performed by applying a cathodic potential of -1.99 V *vs* SCE for 0.8 s and then an anodic potential of 1.35 V *vs* SCE for 0.8 s. The measurement was carried out using a Pt disc working electrode (2 mm diameter), a Ag wire quasi-reference electrode, and a Pt wire counter electrode. PMT bias of 750 V; 000.0 µA amplification.

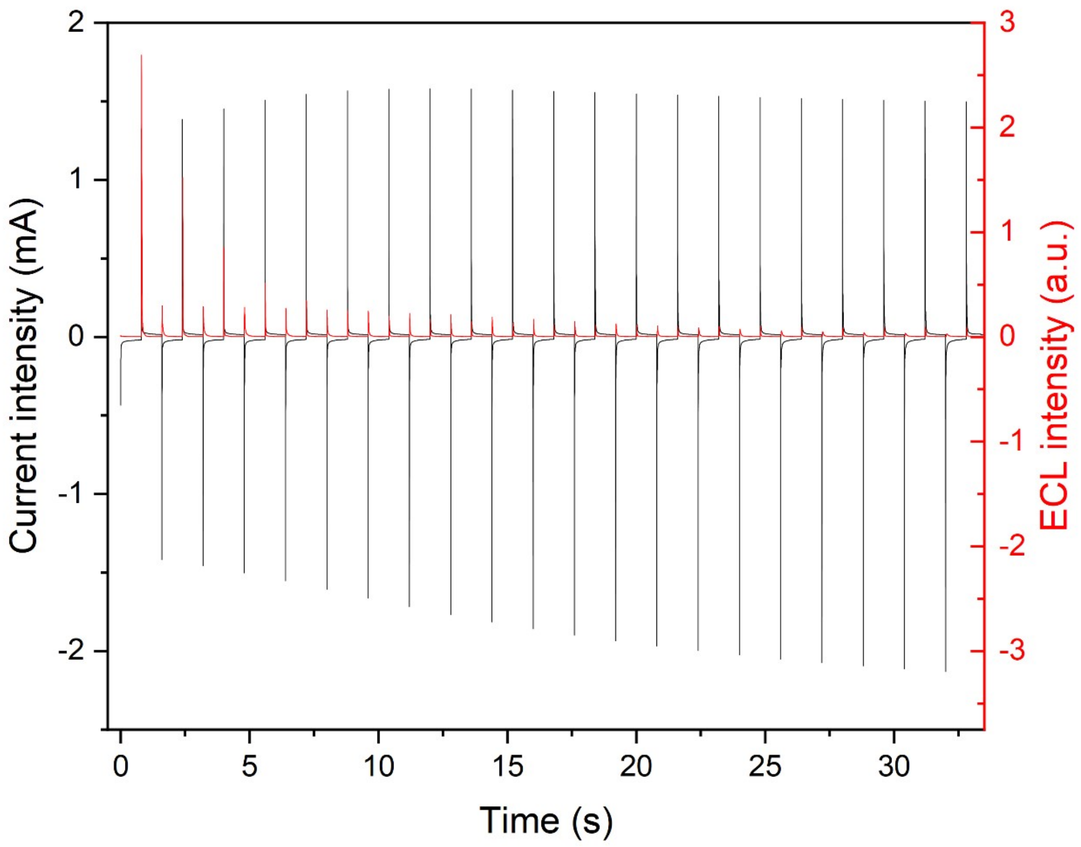

Figure S13. 21 annihilation cycles reporting current (black line) and ECL intensity (red line) of 3DPAImIPN (0.5mM) in AcN/TBAH 0.1 M collected versus time. The annihilation was performed by applying a cathodic potential of -1.62 V *vs* SCE for 0.8 s and then an anodic potential of 1.45 V *vs* SCE for 0.8 s. The measurement was carried out using a Pt disc working electrode (2 mm diameter), a Ag wire quasi-reference electrode, and a Pt wire counter electrode. PMT bias of 750 V; 000.0 µA amplification.

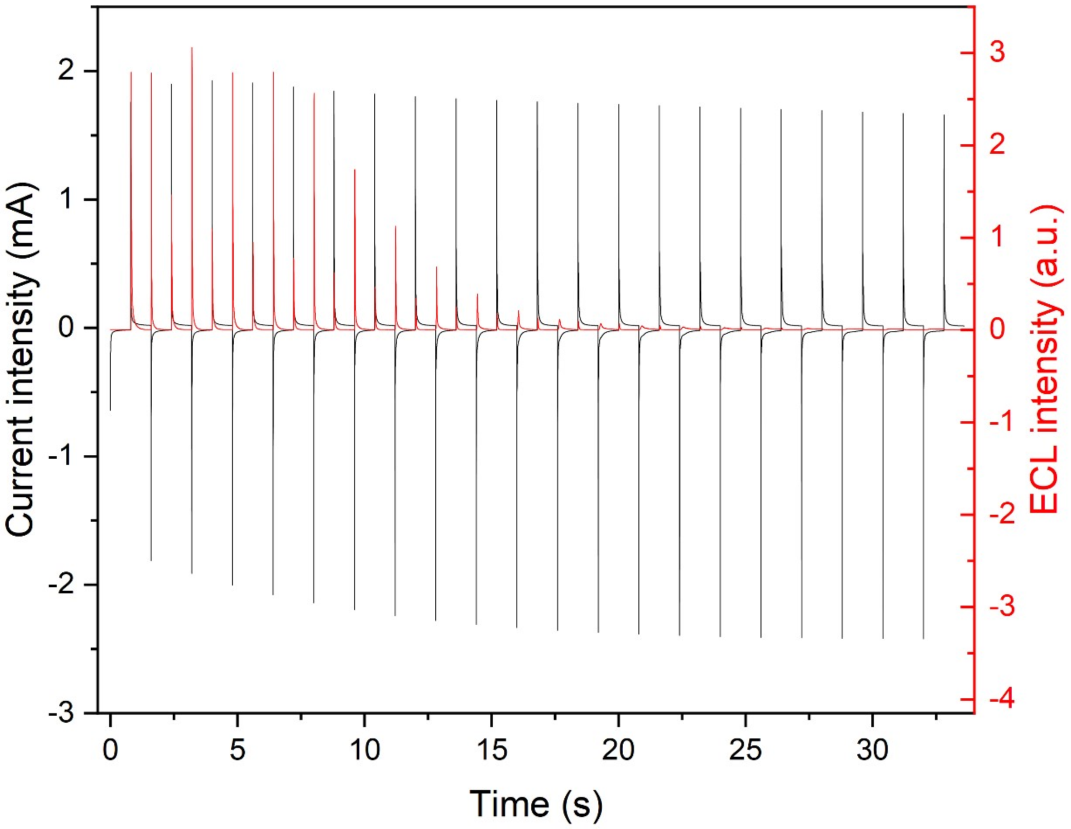

Figure S14. 21 annihilation cycles reporting current (black line) and ECL intensity (red line) of 3DPA2ImBn (0.5mM) in AcN/TBAH 0.1 M collected versus time. The annihilation was performed by applying a cathodic potential of -1.81 V *vs* SCE for 0.8 s and then an anodic potential of 1.45 V *vs* SCE for 0.8 s. The measurement was carried out using a Pt disc working electrode (2 mm diameter), a Ag wire quasi-reference electrode, and a Pt wire counter electrode. PMT bias of 750 V; 000.0 µA amplification.

Coreactant CV-ECL of acceptor-modified compounds

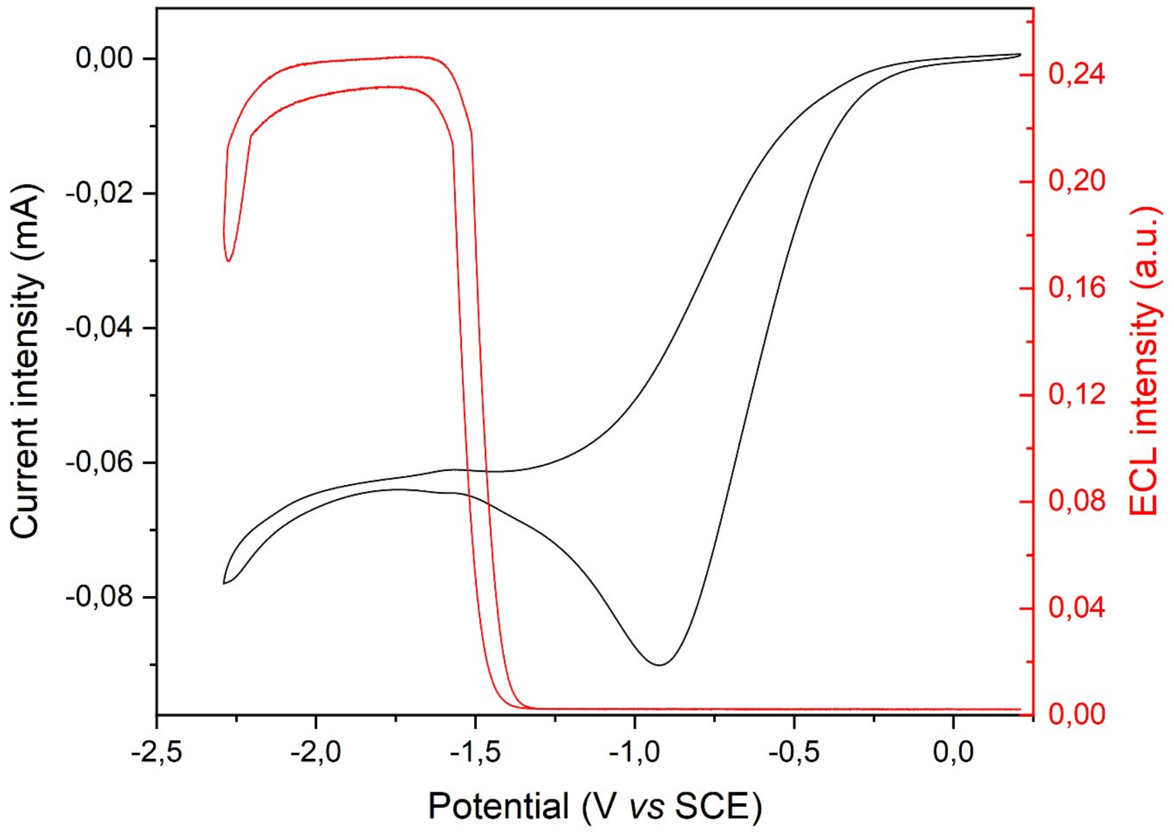

Figure S15. Current (black line) and ECL intensity (red line) of 3DPAFIPN (0.5 mM) in degassed AcN/$TBAPF_6$ 0.1 M/BPO 10 mM collected versus the applied potential. The measurement was performed with a Pt disc working electrode (2 mm diameter), a Ag wire quasi-reference electrode, and a Pt wire counter electrode. The scan rate was set to 100 mV/s. PMT bias of 750 V; 0.0 mA amplification.

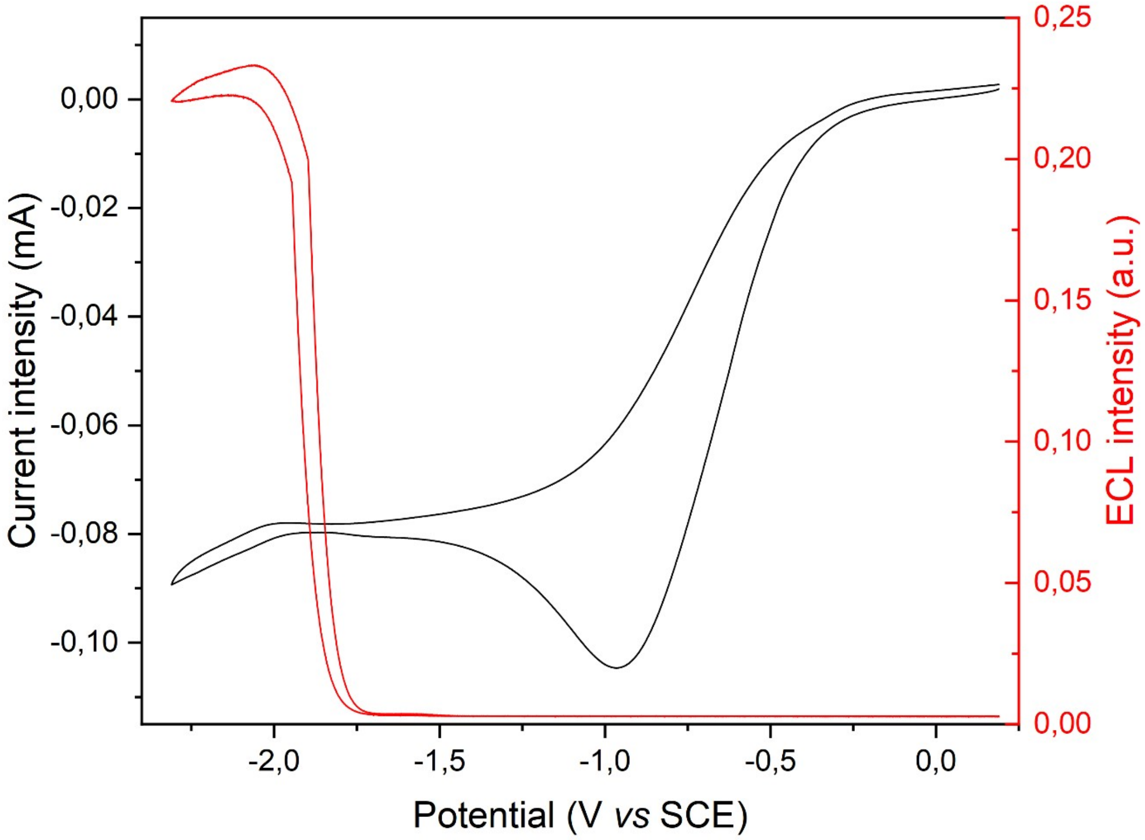

Figure S16. Current (black line) and ECL intensity (red line) of 3DPA2FBn (0.5 mM) in degassed AcN/$TBAPF_6$ 0.1 M/BPO 10 mM collected versus the applied potential. The measurement was performed with a Pt disc working electrode (2 mm diameter), a Ag wire quasi-reference electrode, and a Pt wire counter electrode. The scan rate was set to 100 mV/s. PMT bias of 750 V; 0.0 mA amplification.

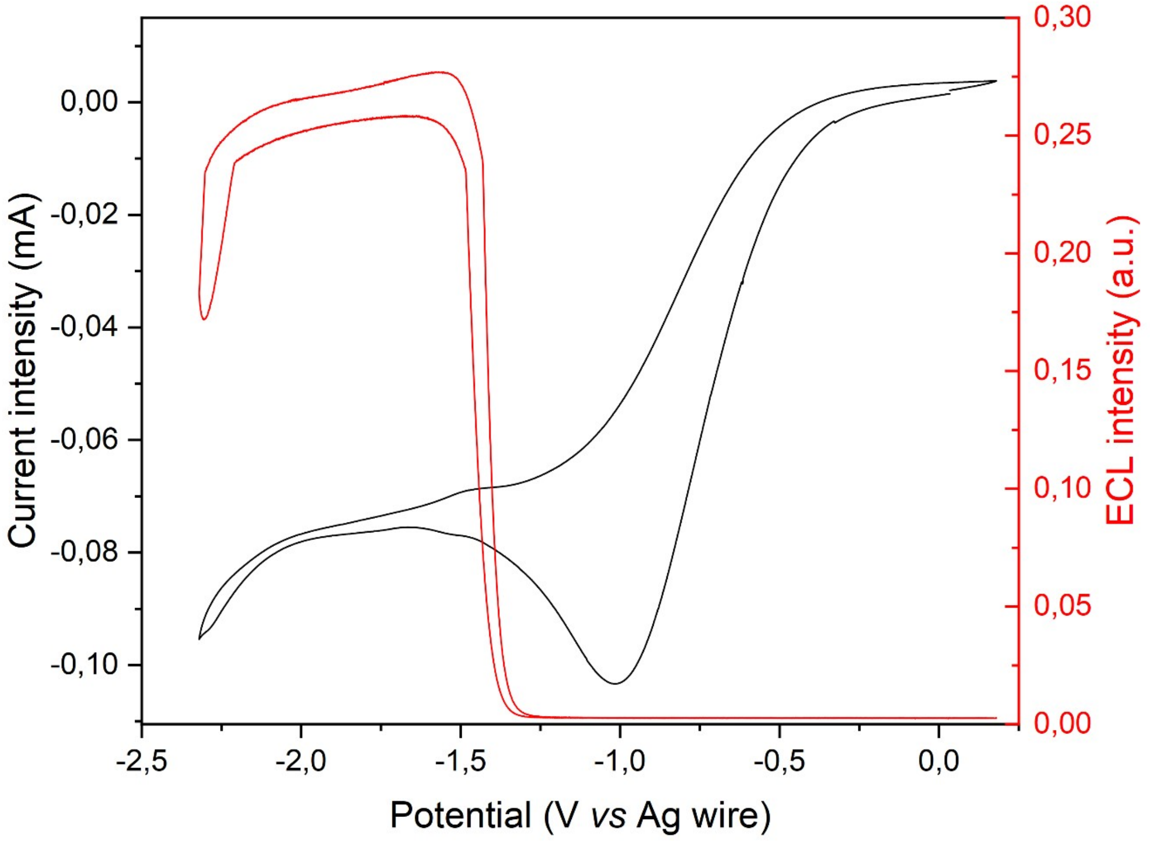

Figure S17. Current (black line) and ECL intensity (red line) of 3DPAImIPN (0.5 mM) in degassed AcN/$TBAPF_6$ 0.1 M/BPO 10 mM collected versus the applied potential. The measurement was performed with a Pt disc working electrode (2 mm diameter), a Ag wire quasi-reference electrode, and a Pt wire counter electrode. The scan rate was set to 100 mV/s. PMT bias of 750 V; 0.0 mA amplification.

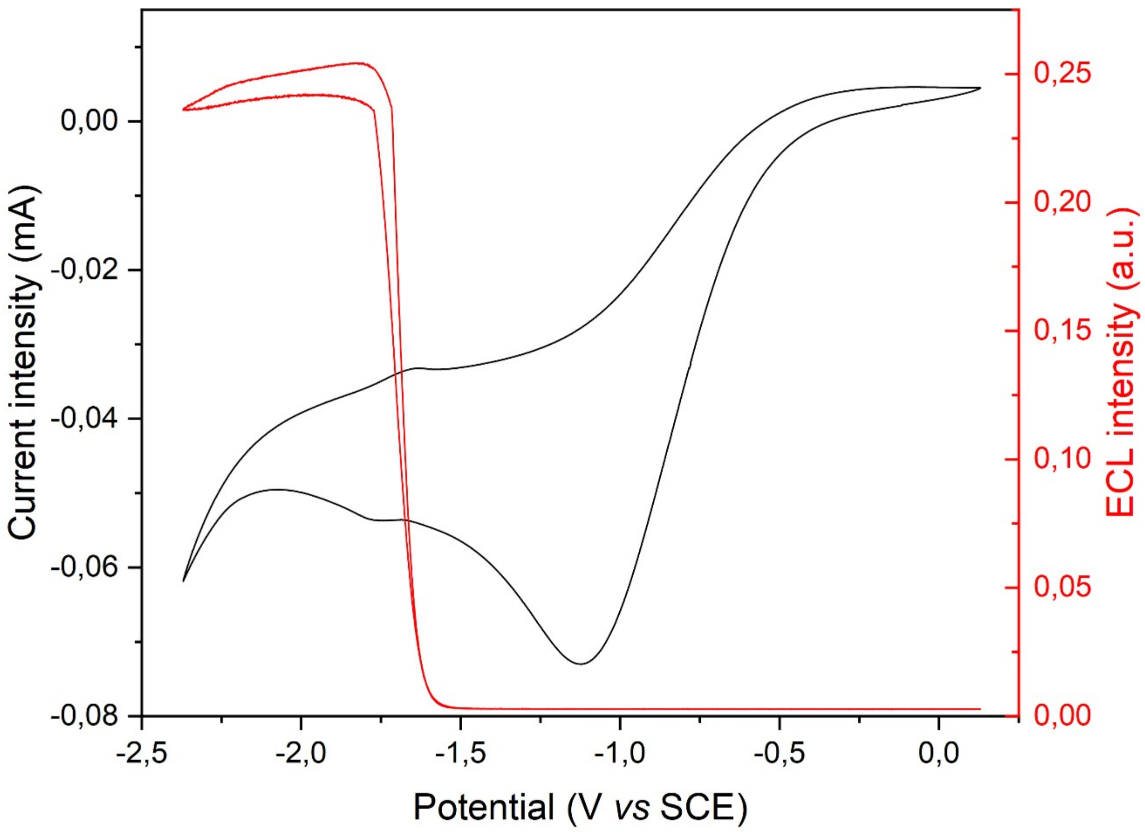

Figure S18. Current (black line) and ECL intensity (red line) of 3DPA2ImBn (0.5 mM) in degassed AcN/$TBAPF_6$ 0.1 M/BPO 10 mM collected versus the applied potential. The measurement was performed with a Pt disc working electrode (2 mm diameter), a Ag wire quasi-reference electrode, and a Pt wire counter electrode. The scan rate was set to 100 mV/s. PMT bias of 750 V; 0.0 mA amplification.

Photophysics of donor-modified compounds

Absorption, emission spectra and lifetimes

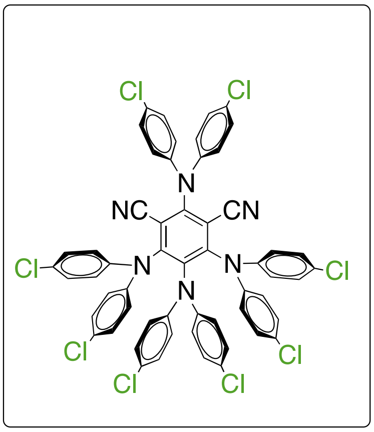

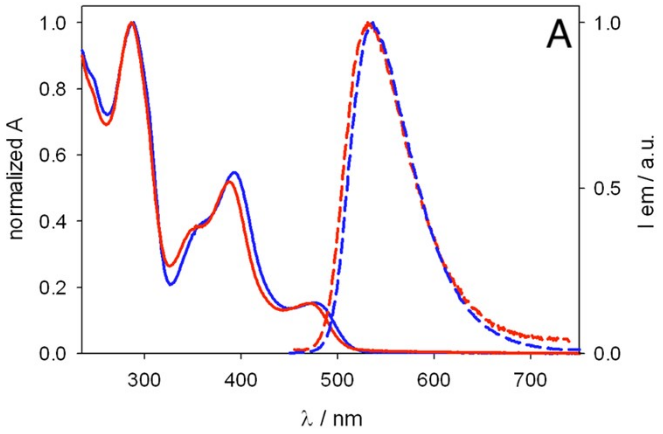

	Absorption	Emission						
	λ (nm) / ε ($M^{-1}cm^{-1}$)	λ_{max} (nm)	$\tau_{prompt, a.e.}$ (ns)	$\tau_{prompt, deg.}$ (ns)	$\tau_{del., a.e.}$ (μs)	$\tau_{del., deg.}$ (μs)	$\Phi_{em, a.e.}$	$\Phi_{em, deg.}$
**DCM**	478 / 7400	536	1.9	2.0	2.7	36	0.053	0.22
**MeCN**	470 / -	531	2.3	2.3	1.5	47	0.050	0.37

Figure S19. A: comparison between normalized absorption (solid lines) and emission spectra (dashed lines) obtained from solutions of **4(Cl_2DPA)IPN** in CH_2Cl_2 (blue) and CH_3CN (red). Key photophysical data are also shown.

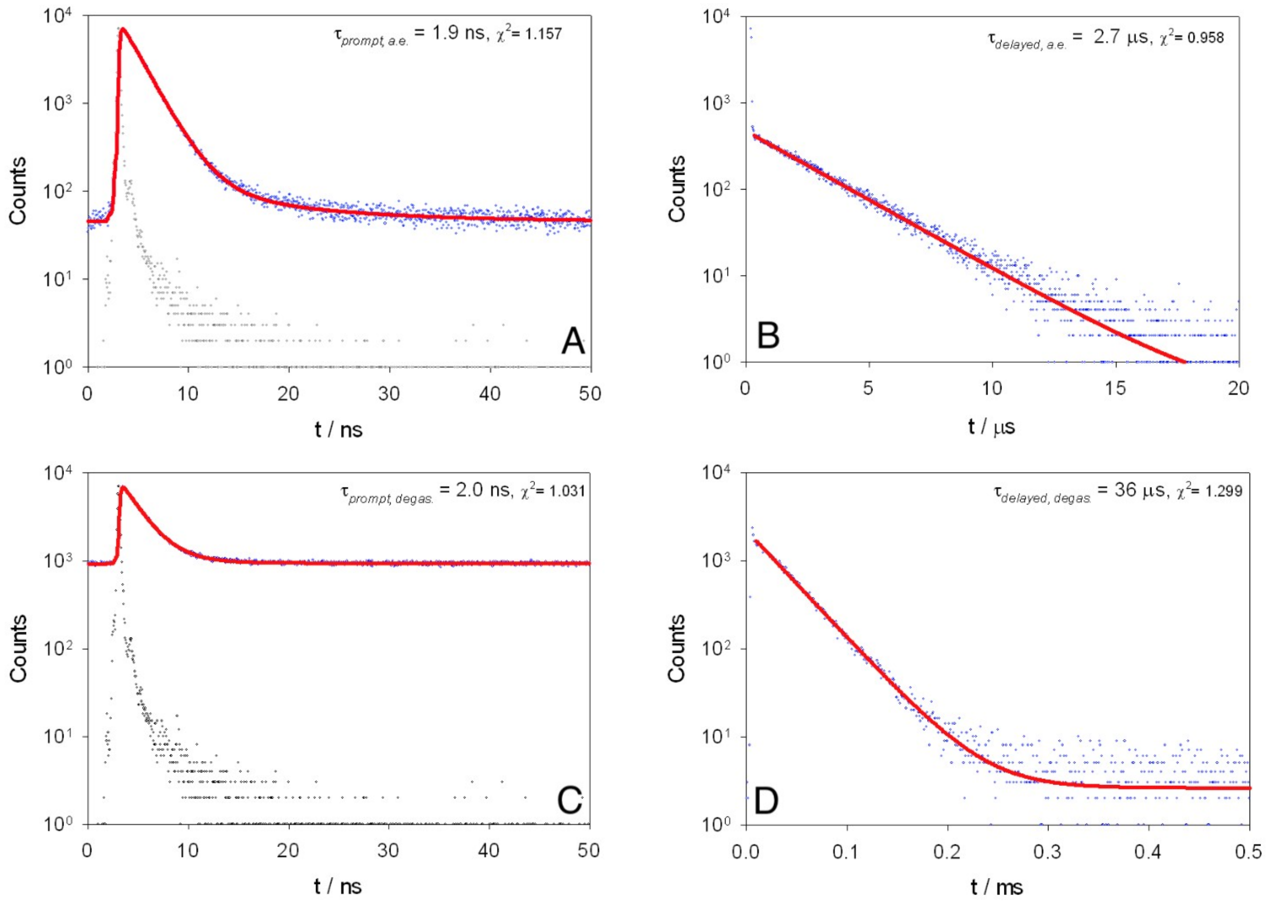

Figure S20. Emission decays for prompt and delayed fluorescence recorded for solutions of **4(Cl$_2$DPA)IPN** in air-equilibrated (A-B) and deaerated (C-D) CH_2Cl_2 at RT. The calculated monoexponentail fitting functions are shown as red lines. The instrumental response function (IRF) is also shown as grey dots.

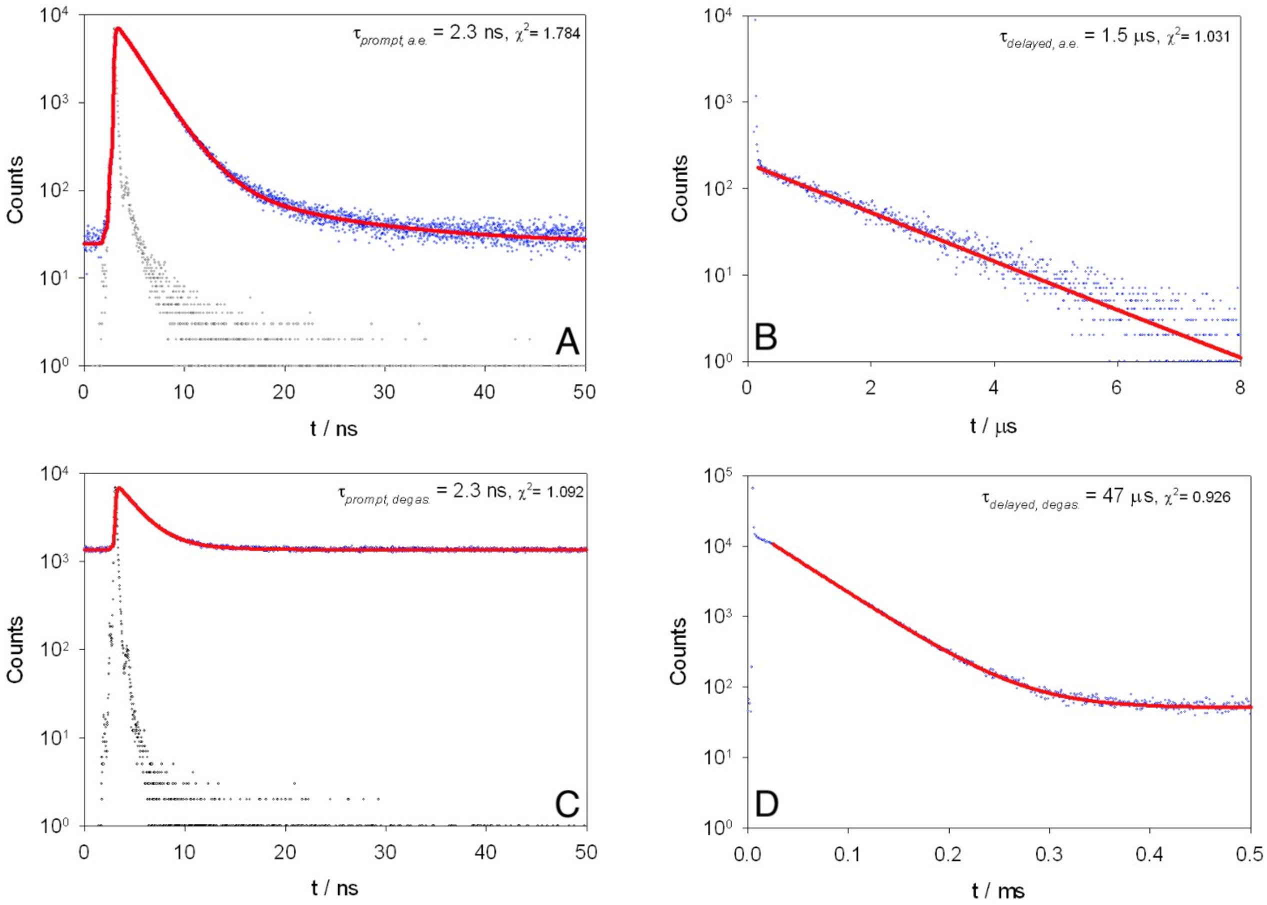

Figure S21. Emission decays for prompt and delayed fluorescence recorded for solutions of **4(Cl_2DPA)IPN** in air-equilibrated (A-B) and deaerated (C-D) CH_3CN at RT. The calculated monoexponentail fitting functions are shown as red lines. The instrumental response function (IRF) is also shown as grey dots.

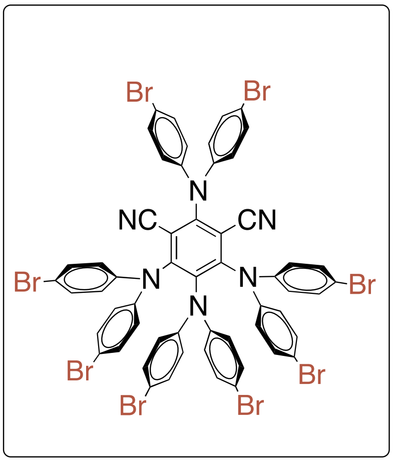

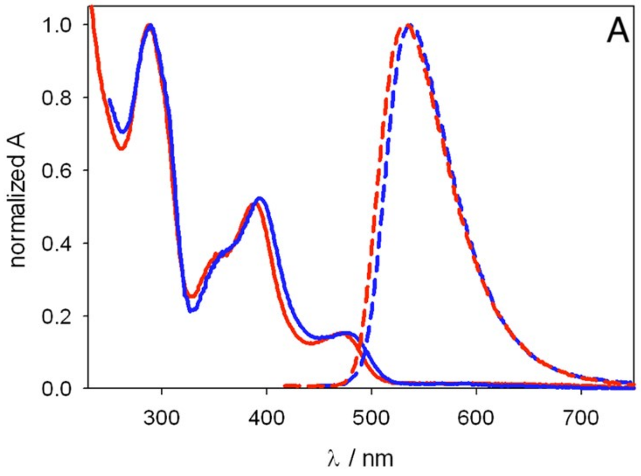

	Absorption	Emission						
	λ (nm) / ε ($M^{-1}cm^{-1}$)	λ_{max} (nm)	$\tau_{prompt, a.e.}$ (ns)	$\tau_{prompt, deg.}$ (ns)	$\tau_{del., a.e.}$ (µs)	$\tau_{del., deg.}$ (µs)	$\Phi_{em, a.e.}$	$\Phi_{em, deg.}$
**DCM**	476 / 8400	537	0.5	0.5	2.2	8.2	0.031	0.16
**MeCN**	471 / -	533	0.5	0.5	1.8	12	0.026	0.21

Figure S22. A: comparison between normalized absorption (solid lines) and emission spectra (dashed lines) obtained from solutions of **4(Br$_2$DPA)IPN** in CH_2Cl_2 (blue) and CH_3CN (red). Key photophysical data are also shown.

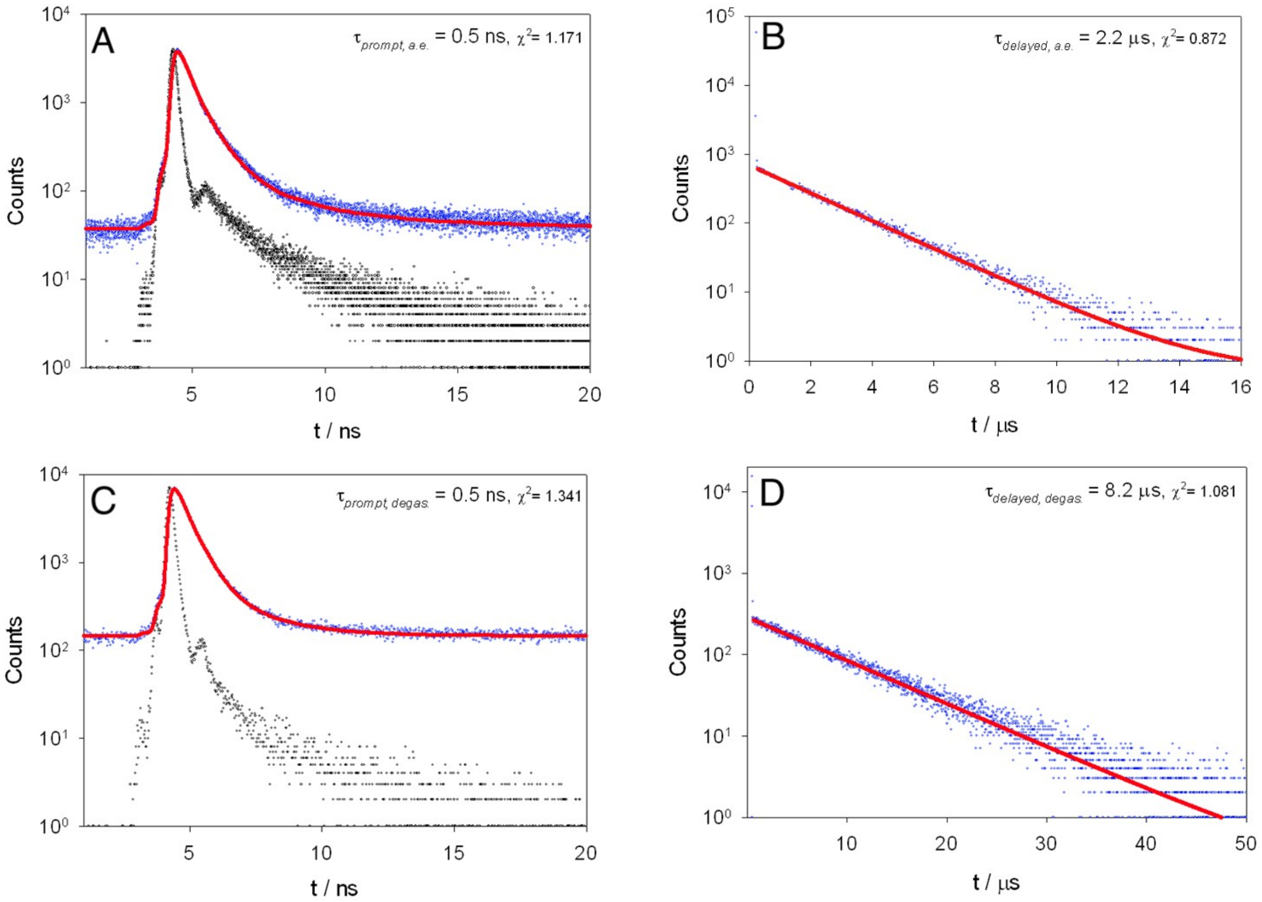

Figure S23. Emission decays for prompt and delayed fluorescence recorded for solutions of **4(Br$_2$DPA)IPN** in air-equilibrated (A-B) and deaerated (C-D) CH_2Cl_2 at RT. The calculated monoexponentail fitting functions are shown as red lines. The instrumental response function (IRF) is also shown as grey dots.

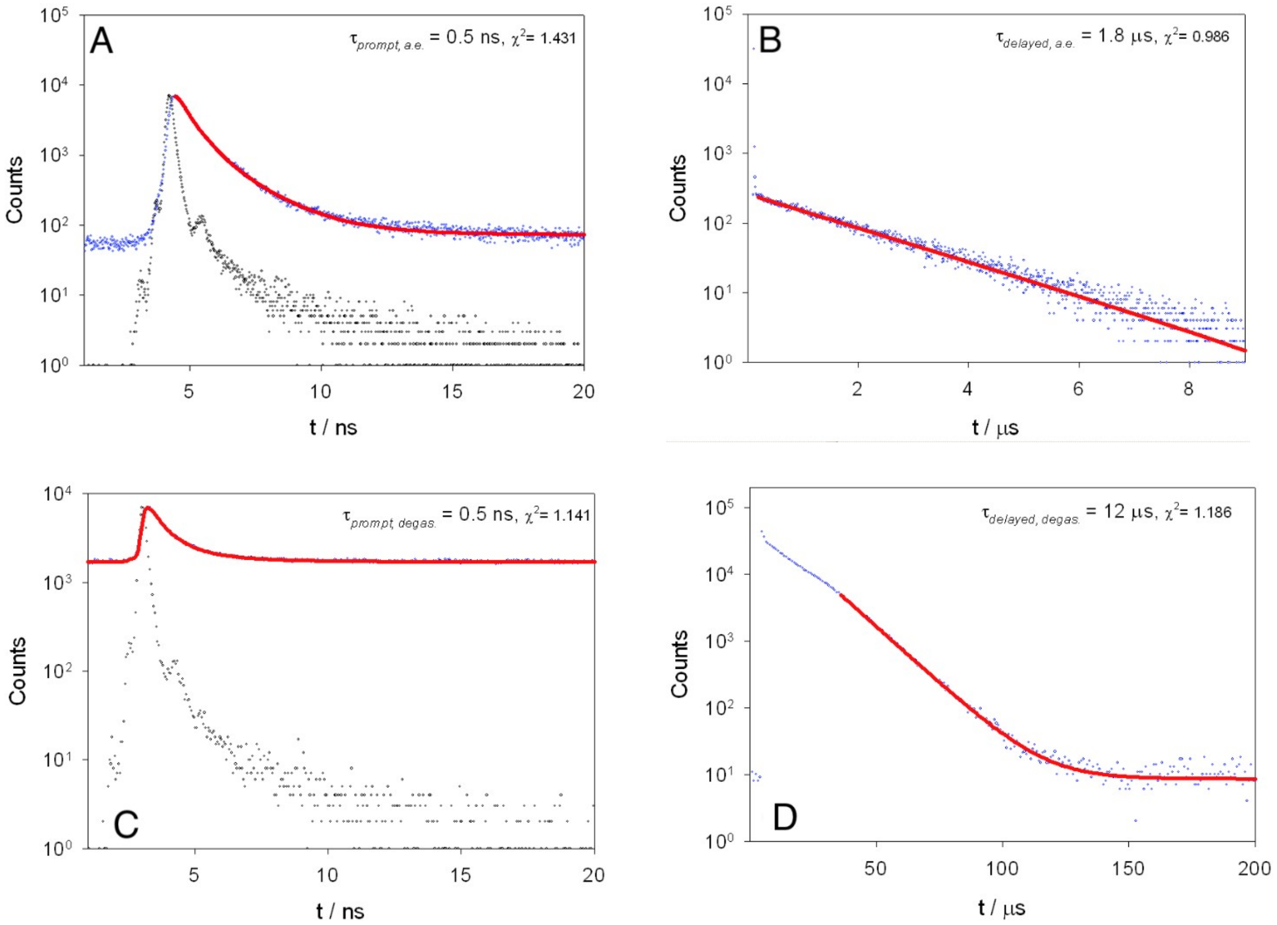

Figure S24. Emission decays for prompt and delayed fluorescence recorded for solutions of **4(Br_2DPA)IPN** in air-equilibrated (A-B) and deaerated (C-D) CH_3CN at RT. The calculated monoexponentail fitting functions are shown as red lines. The instrumental response function (IRF) is also shown as grey dots.

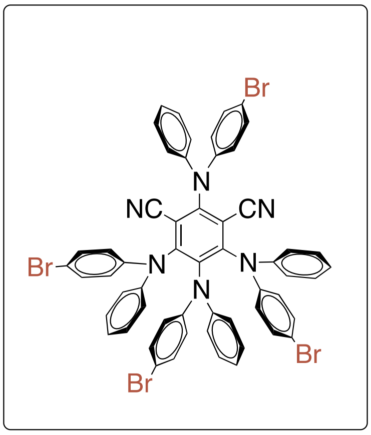

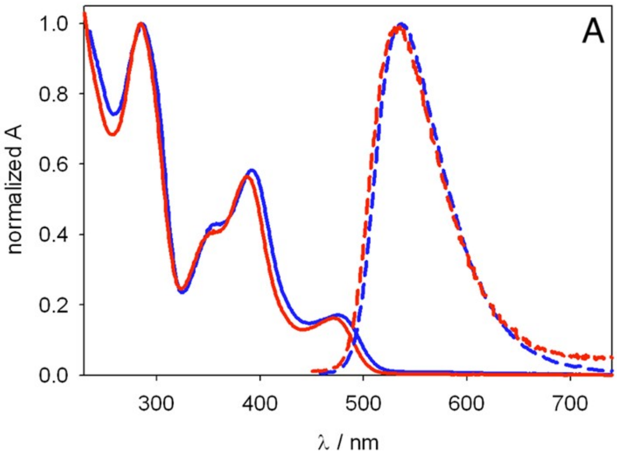

	Absorption	Emission						
	λ (nm) / ε ($M^{-1}cm^{-1}$)	λ_{max} (nm)	$\tau_{prompt, a.e.}$ (ns)	$\tau_{prompt, deg.}$ (ns)	$\tau_{del., a.e.}$ (μs)	$\tau_{del., deg.}$ (μs)	$\Phi_{em, a.e.}$	$\Phi_{em, deg.}$
**DCM**	475 / 8000	536	1.5 [a]	1.6 [a]	1.4	46	0.028	0.27
**MeCN**	471 / -	532	1.4	1.5 [a]	0.96	20	0.036	0.15

[a] Multiexponential decay (longest component considered).

Figure S25. A: comparison between normalized absorption (solid lines) and emission spectra (dashed lines) obtained from solutions of **4(BrDPA)IPN** in CH_2Cl_2 (blue) and CH_3CN (red). Key photophysical data are also shown.

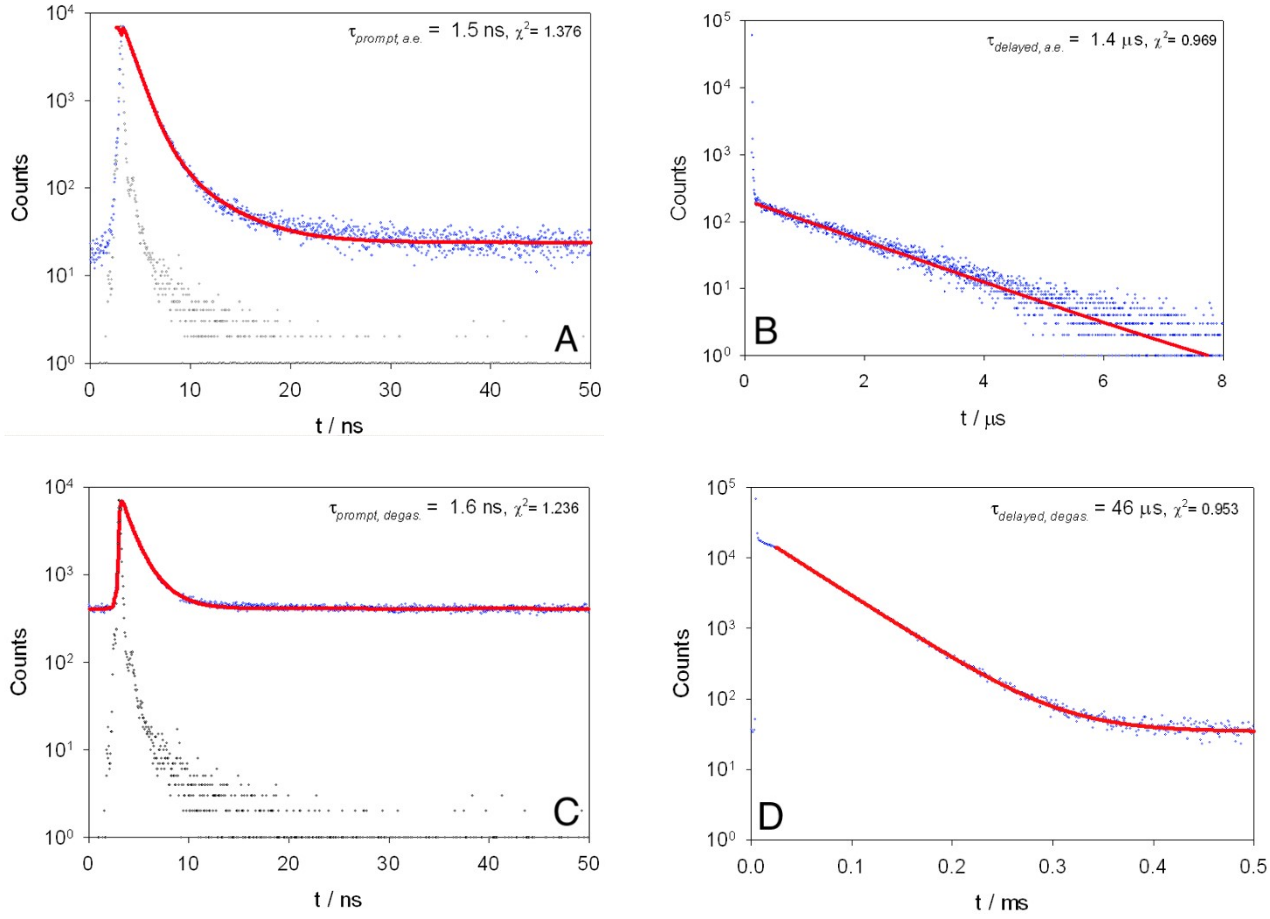

Figure S26. Emission decays for prompt and delayed fluorescence recorded for solutions of **4(BrDPA)IPN** in air-equilibrated (A-B) and deaerated (C-D) CH_2Cl_2 at RT. The calculated monoexponentail fitting functions are shown as red lines. The instrumental response function (IRF) is also shown as grey dots.

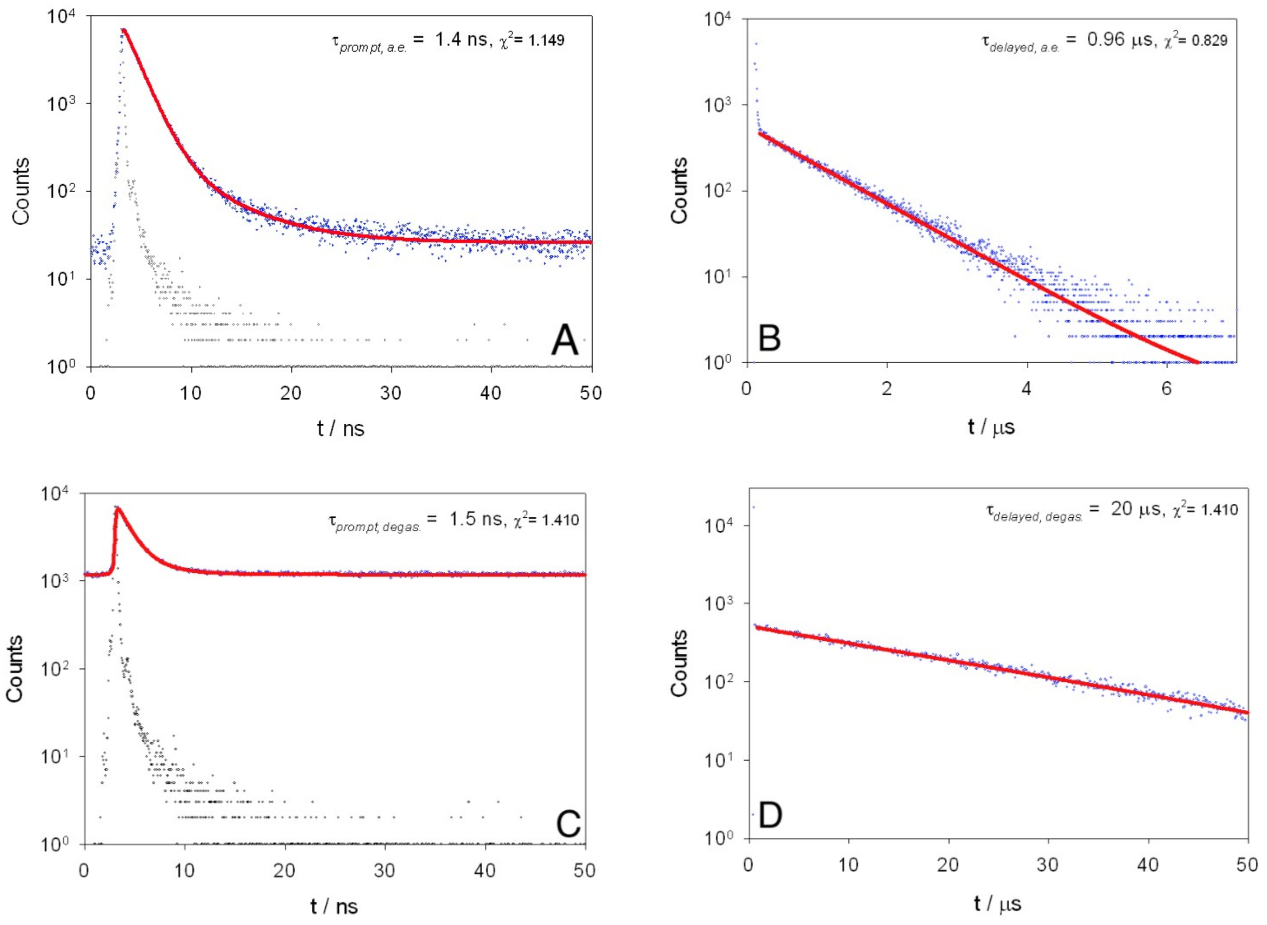

Figure S27. Emission decays for prompt and delayed fluorescence recorded for solutions of **4(BrDPA)IPN** in air-equilibrated (A-B) and deaerated (C-D) CH_3CN at RT. The calculated monoexponentail fitting functions are shown as red lines. The instrumental response function (IRF) is also shown as grey dots.

Computed DFT data for donor-modified molecules

Table S3. Frontier orbital energies (E_{HOMO} and E_{LUMO}) from B3LYP-D3/6-31G(d) calculations at the optimized groud state geometry; vertical excitation energies of the lowest singlet and triplet states, $E(S_1)$, $E(T_1)$, oscillator strength of S_1, f, and the singlet-triplet energy difference $\Delta E_{ST}=(E(S_1)- E(T_1))$, from TDA-M06-2X/6-31+G(d) in toluene (solvent described with PCM).

	E_{HOMO} /Hartree(eV)	E_{LUMO} /Hartree(eV)	$E(S_1)$/eV	$f(S_1)$	$E(T_1)$/eV	ΔE_{ST}/eV
**4(Cl_2DPA)IPN**	-0.212(-5.77)	-0.099(-2.69)	3.086	0.2086	2.817	0.269
**4(Br_2DPA)IPN**	-0.210(-5.71)	-0.099(-2.69)	3.056	0.1794	2.802	0.255
**4(BrDPA)IPN**	-0.201(-5.47)	-0.087(-2.37)	3.095	0.2025	2.822	0.273
**4DPAIPN**	-0.190(-5.17)	-0.073(-1.99)	3.110	0.1948	2.829	0.281

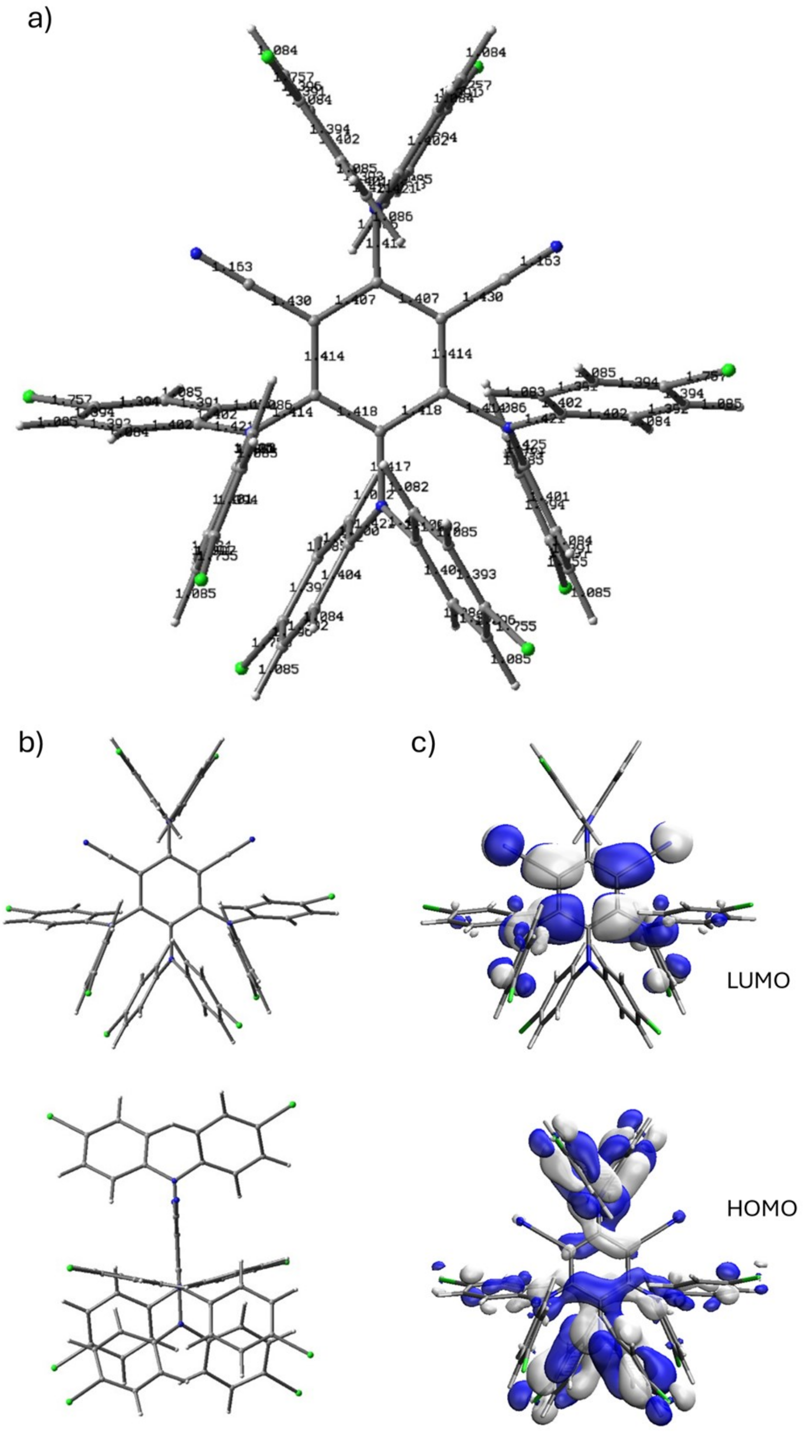

Figure S28. 4(Cl_2DPA)IPN: a) Ground state optimized bond lengths; b) front and side views of the ground state optimized geometry and c) frontier molecular orbitals. From B3LYP-D3/6-31G(d) calculations.

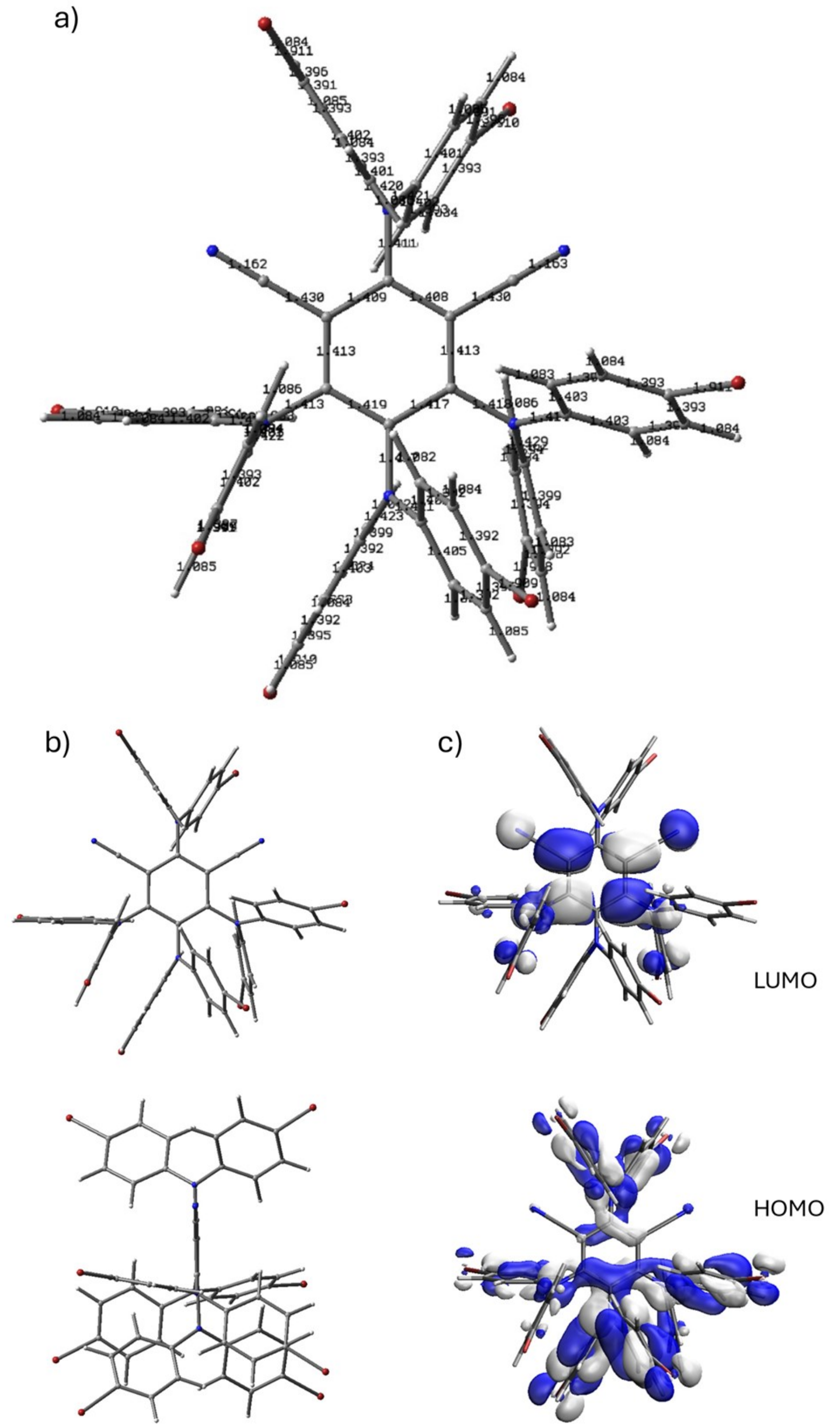

Figure S29. 4(Br_2DPA)IPN: a) Ground state optimized bond lengths; b) front and side views of the ground state optimized geometry and c) frontier molecular orbitals. From B3LYP-D3/6-31G(d) calculations.

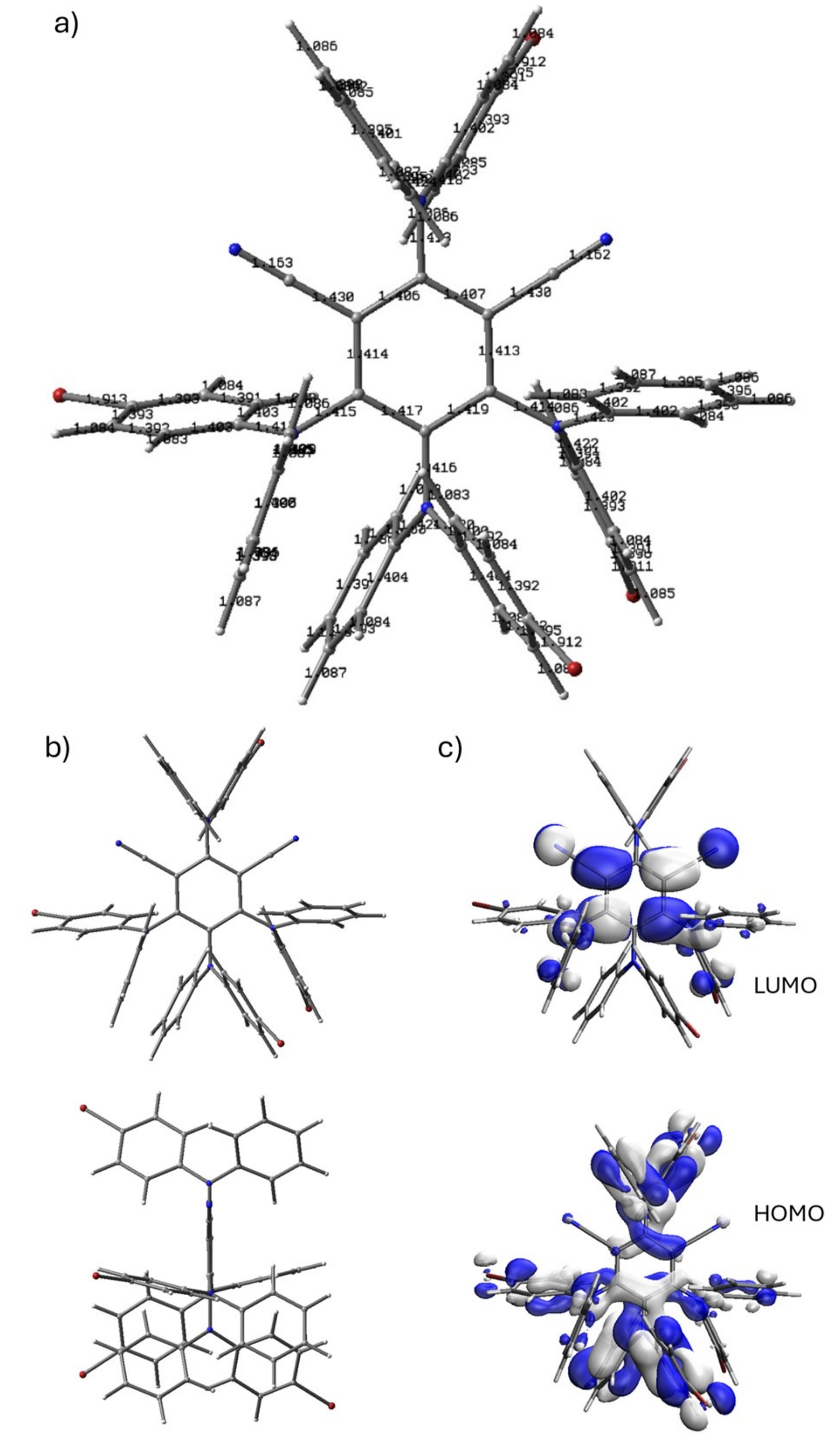

Figure S30. 4(BrDPA)IPN: a) Ground state optimized bond lengths; b) front and side views of the ground state optimized geometry and c) frontier molecular orbitals. From B3LYP-D3/6-31G(d) calculations.

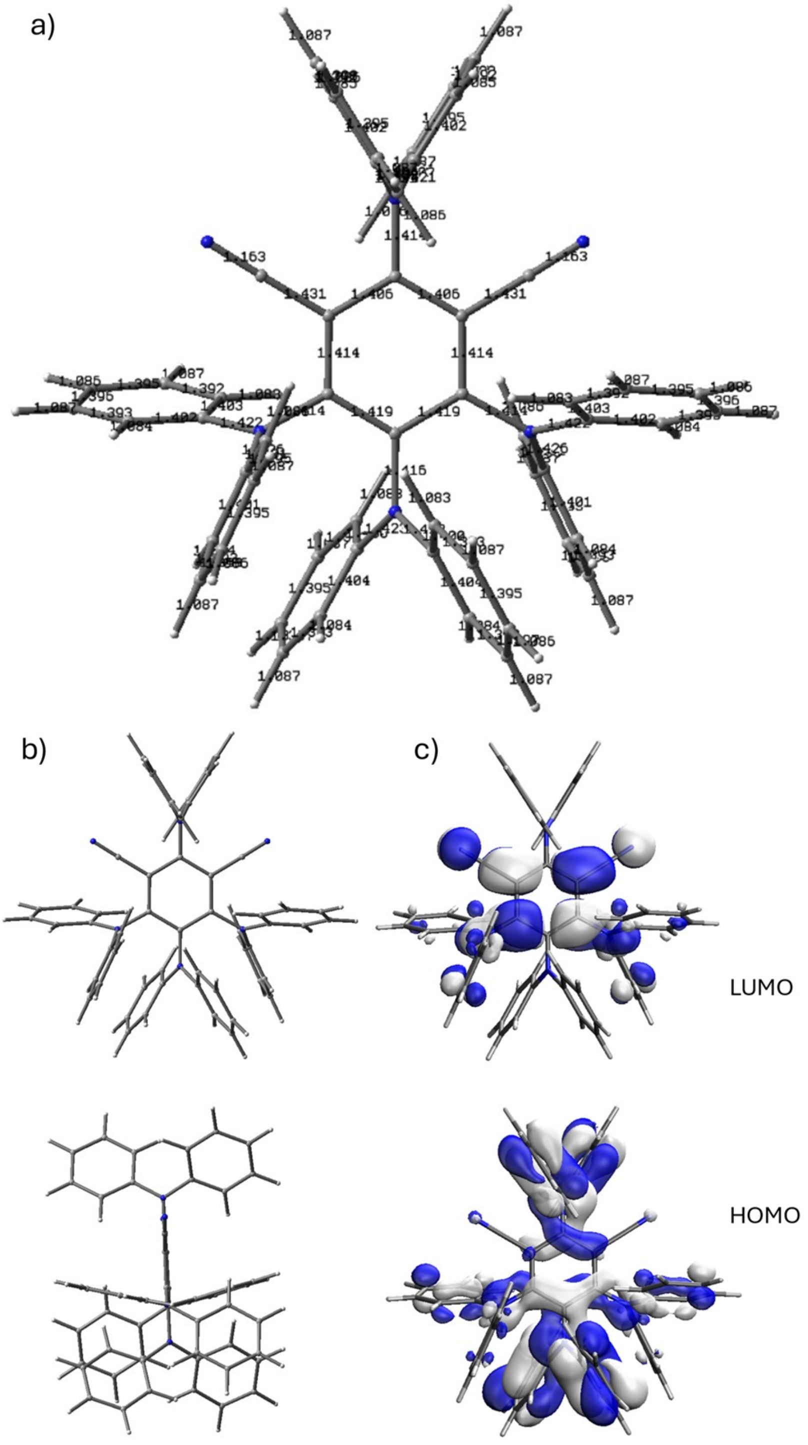

Figure S31. 4DPAIPN: a) Ground state optimized bond lengths; b) front and side views of the ground state optimized geometry and c) frontier molecular orbitals. From B3LYP-D3/6-31G(d) calculations.

Electrochemistry of donor-modified compounds

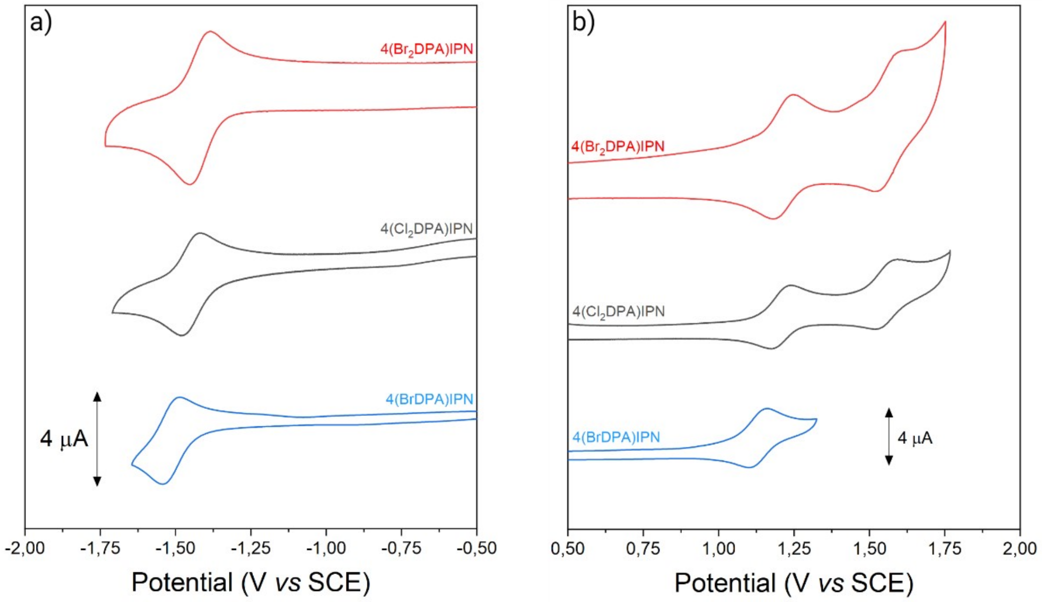

Figure S32. Cyclic voltammograms of the donator-modified molecules, highlighting the (a) cathodic and the (b) anodic regions. Measurements were performed in degassed MeCN solutions containing 1 mM luminophore and 0.1 M TBAPF$_6$ as the supporting electrolyte: 4(Br$_2$DPA)IPN (red line), 4(Cl$_2$DPA)IPN (grey line), and 4(BrDPA)IPN (blue line). Anodic and cathodic voltammograms of the same compound belong to the same scan. The Me$_{10}$Fc$^+$/Me$_{10}$Fc redox couple was used as internal standard. The cyclic voltammetries of 4(Cl$_2$DPA)IPN and 4(BrDPA)IPN were performed at a scan rate of 0.1 V/s, while the cyclic voltammetry of 4(Br$_2$DPA)IPN was performed at 1 V/s.

Electrochemiluminescence of donor-modified compounds

Annihilation ECL

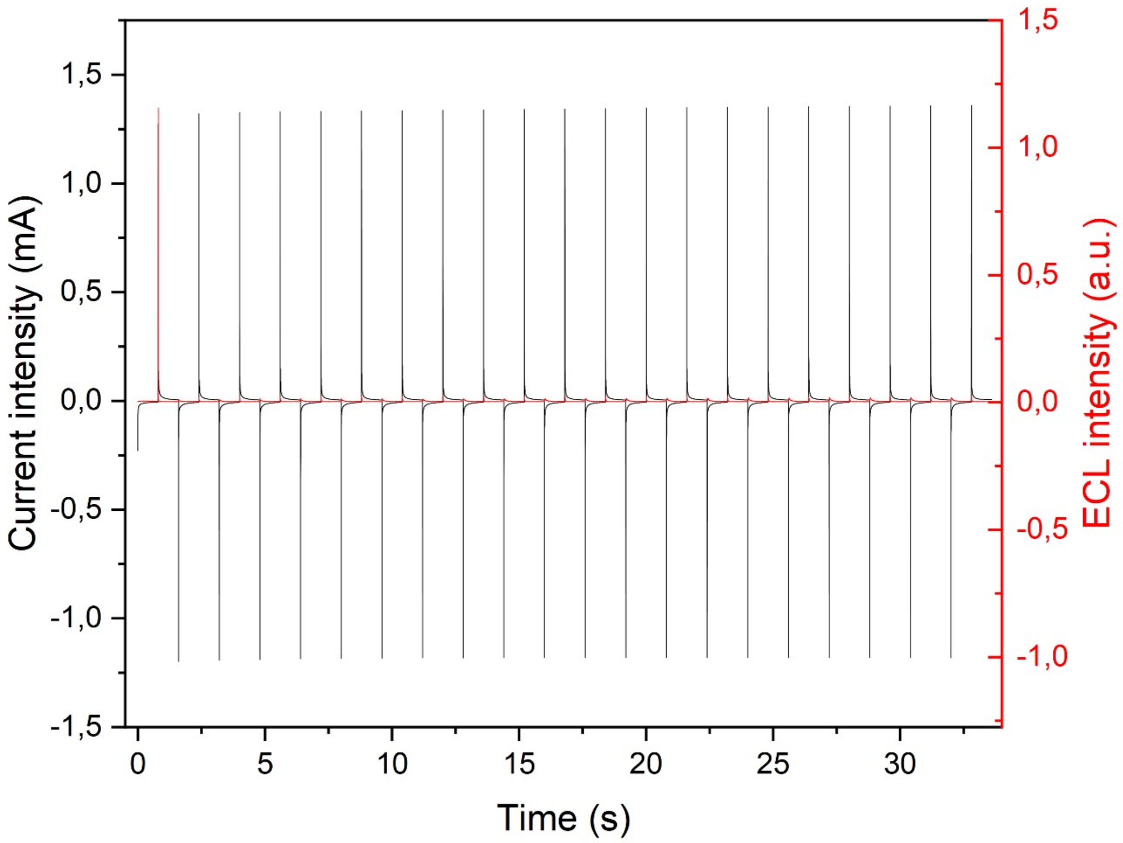

Figure S33. 21 annihilation cycles reporting current (black line) and ECL intensity (red line) of 4(BrDPA)IPN (0.5mM) in AcN/TBAH 0.1 M collected versus time. The annihilation was performed by applying a cathodic potential of -1.65 V *vs* SCE for 0.8 s and then an anodic potential of 1.26 V *vs* SCE for 0.8 s. The measurement was carried out using a Pt disc working electrode (2 mm diameter), a Ag wire quasi-reference electrode, and a Pt wire counter electrode. PMT bias of 750 V; 000.0 µA amplification.

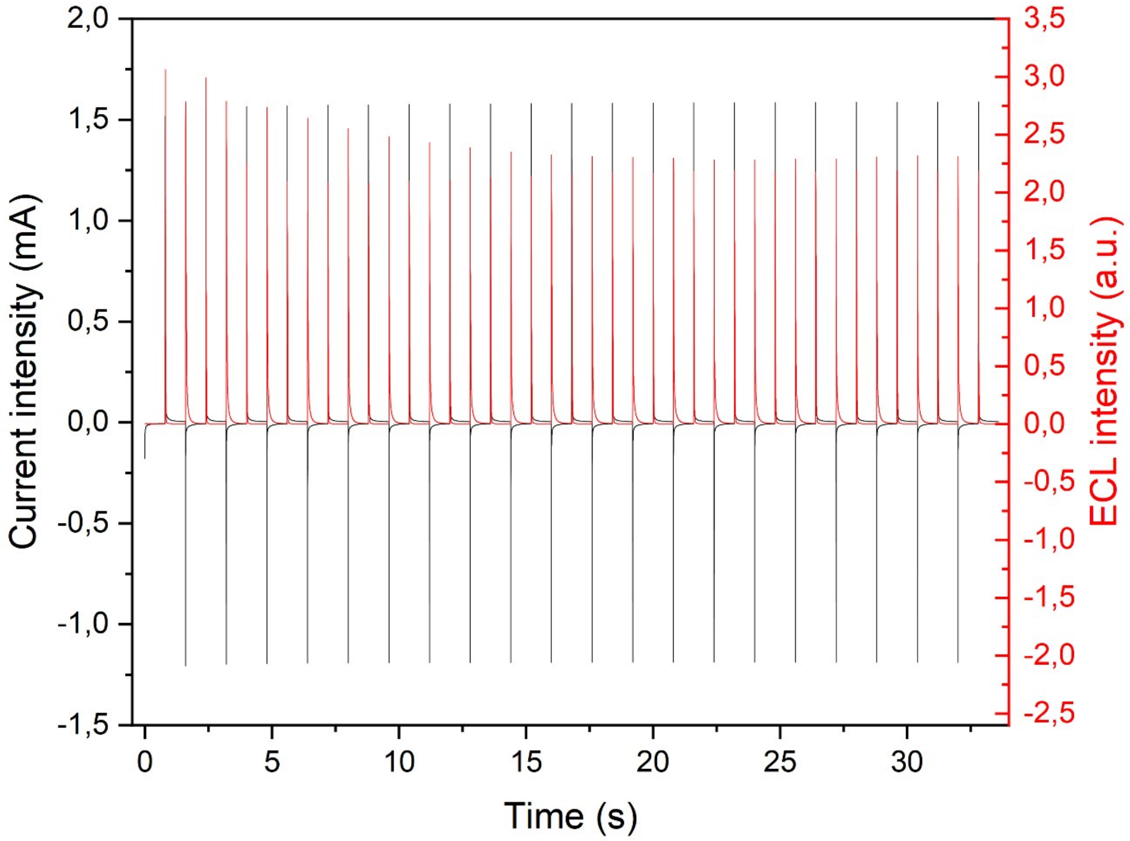

Figure S34. 21 annihilation cycles reporting current (black line) and ECL intensity (red line) of 4(Cl_2DPA)IPN (0.5mM) in AcN/TBAH 0.1 M collected versus time. The annihilation was performed by applying a cathodic potential of -1.58 V *vs* SCE for 0.8 s and then an anodic potential of 1.34 V *vs* SCE for 0.8 s. The measurement was carried out using a Pt disc working electrode (2 mm diameter), a Ag wire quasi-reference electrode, and a Pt wire counter electrode. PMT bias of 750 V; 000.0 μA amplification.

Coreactant CV-ECL of donor-modified compounds

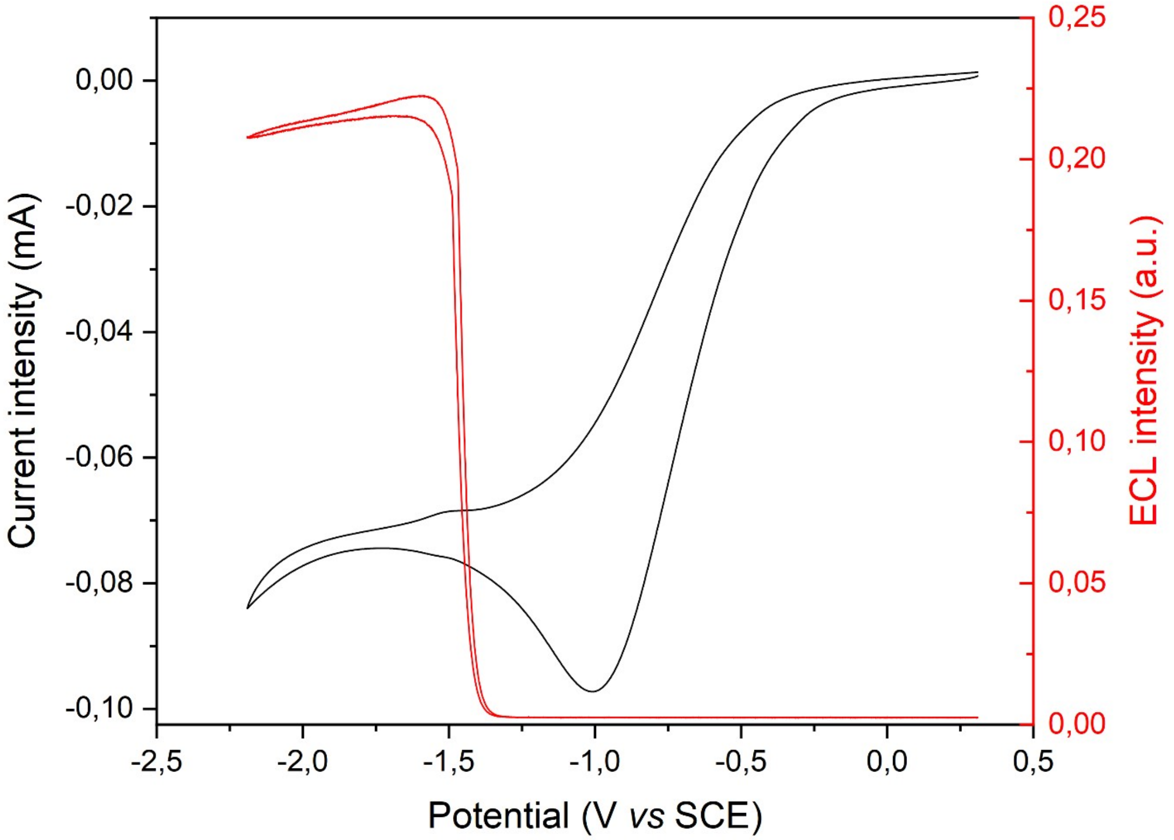

Figure S35. Current (black line) and ECL intensity (red line) of 4(BrDPA)IPN (0.5 mM) in degassed AcN/$TBAPF_6$ 0.1 M/BPO 10 mM collected versus the applied potential. The measurement was performed with a Pt disc working electrode (2 mm diameter), a Ag wire quasi-reference electrode, and a Pt wire counter electrode. The scan rate was set to 100 mV/s. PMT bias of 750 V; 0.0 mA amplification.

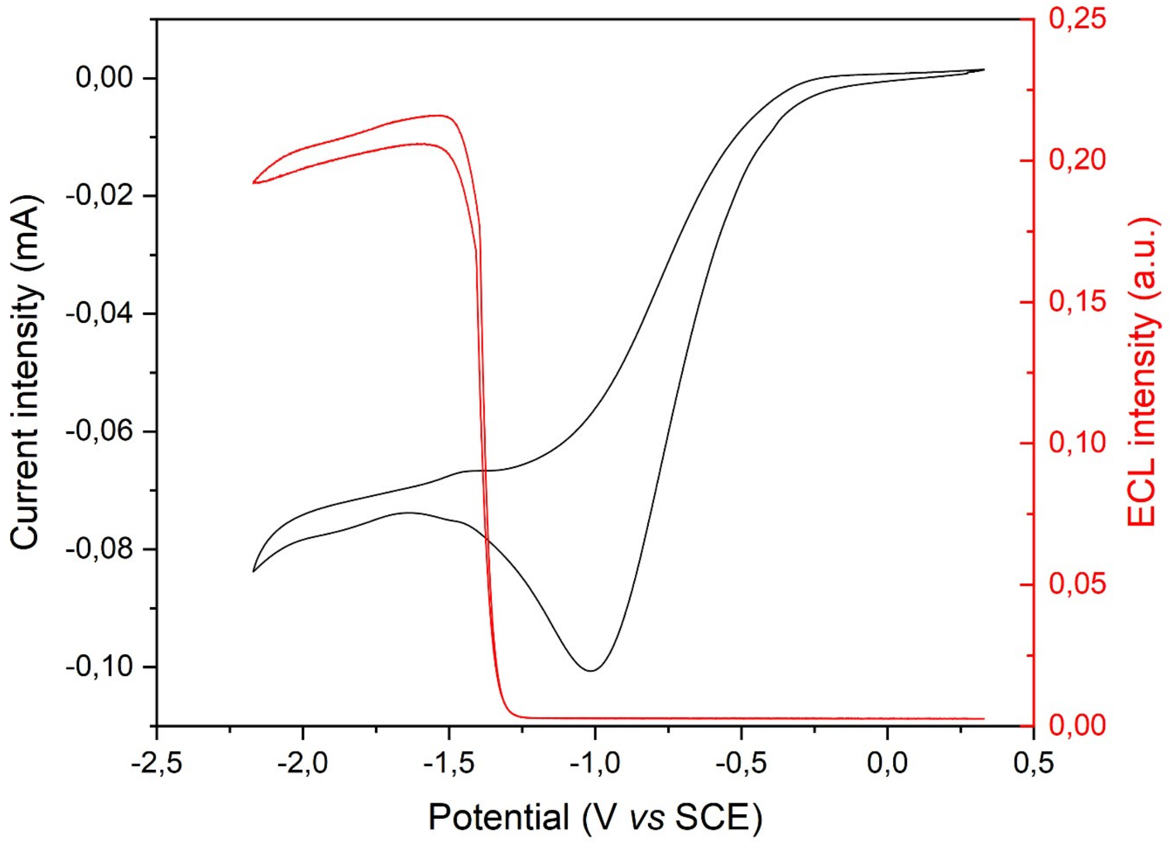

Figure S36. Current (black line) and ECL intensity (red line) of 4(Cl_2DPA)IPN (0.5 mM) in degassed AcN/$TBAPF_6$ 0.1 M/BPO 10 mM collected versus the applied potential. The measurement was performed with a Pt disc working electrode (2 mm diameter), a Ag wire quasi-reference electrode, and a Pt wire counter electrode. The scan rate was set to 100 mV/s. PMT bias of 750 V; 0.0 mA amplification.

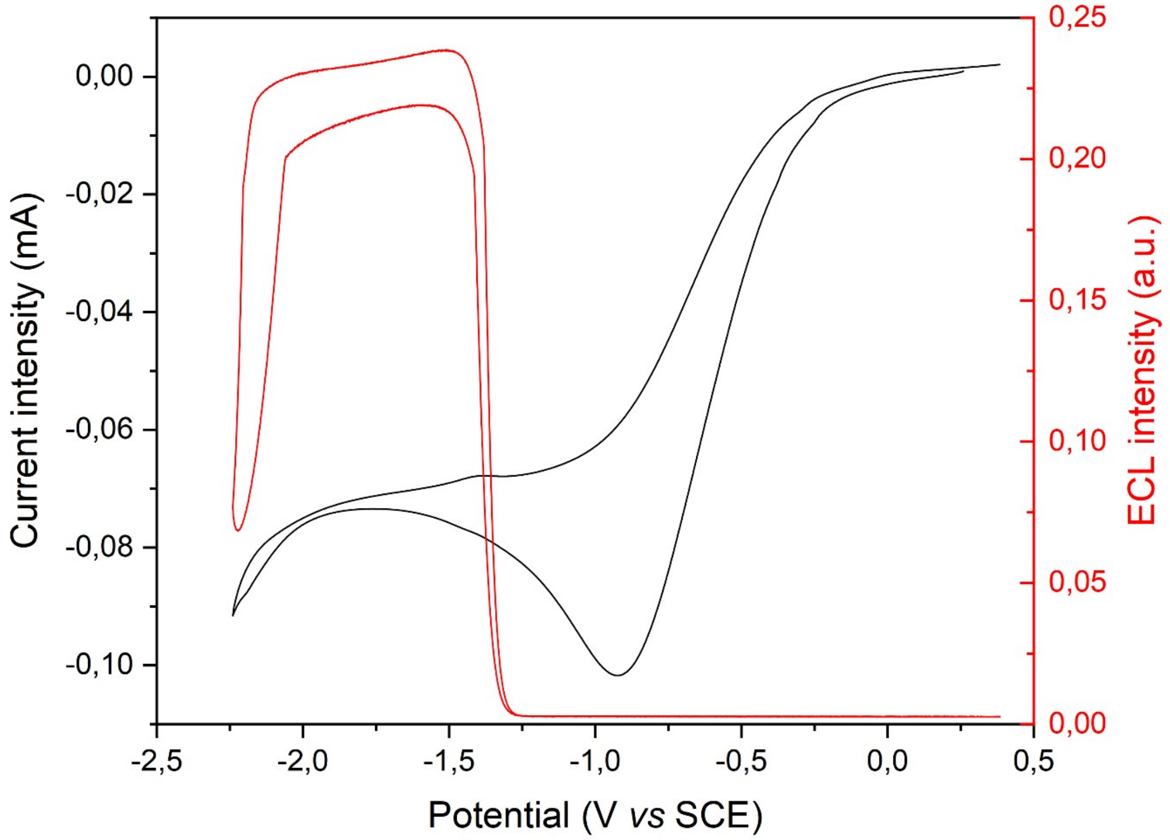

Figure S37. Current (black line) and ECL intensity (red line) of 4(Br_2DPA)IPN (0.5 mM) in degassed AcN/$TBAPF_6$ 0.1 M/BPO 10 mM collected versus the applied potential. The measurement was performed with a Pt disc working electrode (2 mm diameter), a Ag wire quasi-reference electrode, and a Pt wire counter electrode. The scan rate was set to 100 mV/s. PMT bias of 750 V; 0.0 mA amplification.

Photophysical characterization of water-soluble compounds

Absorption spectra

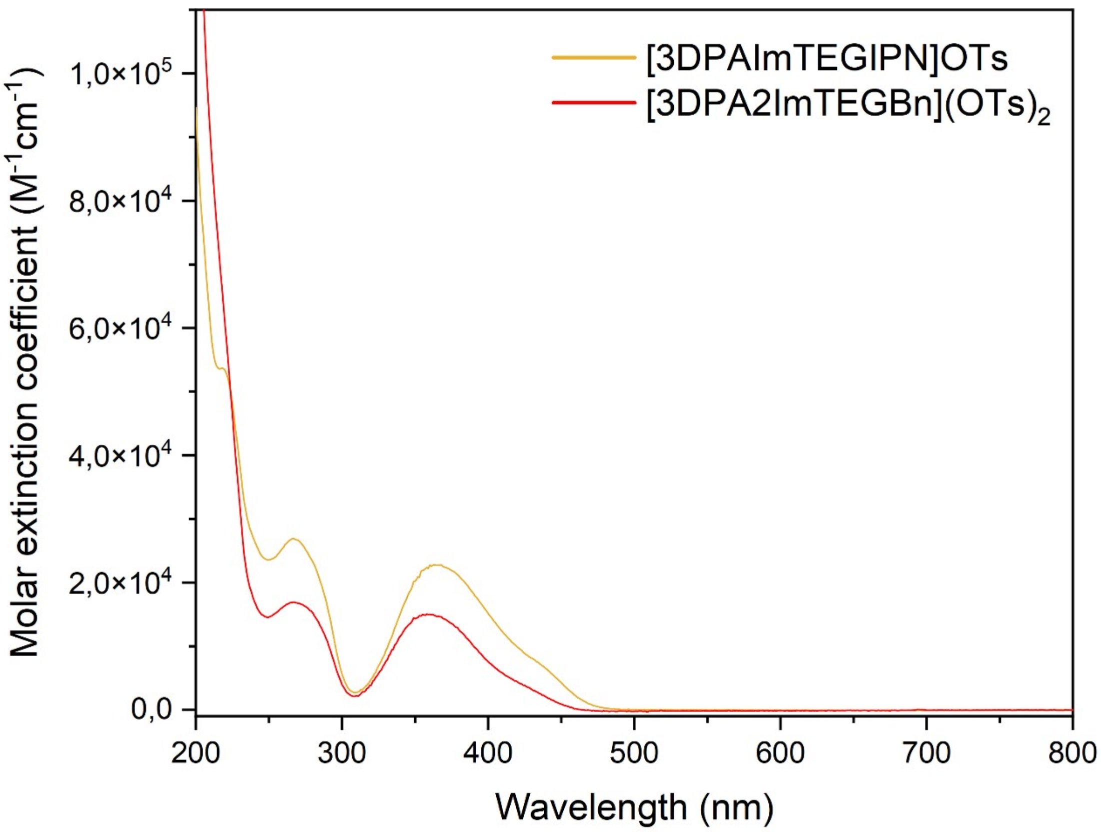

Figure S38. UV-vis spectra of $5\cdot10^{-6}$ M solutions of [3DPA(ImTEG)IPN]OTs (yellow line), and [3DPA2(ImTEG)Bn]$(OTs)_2$ (red line) in non-degassed acetonitrile.

Emission spectra

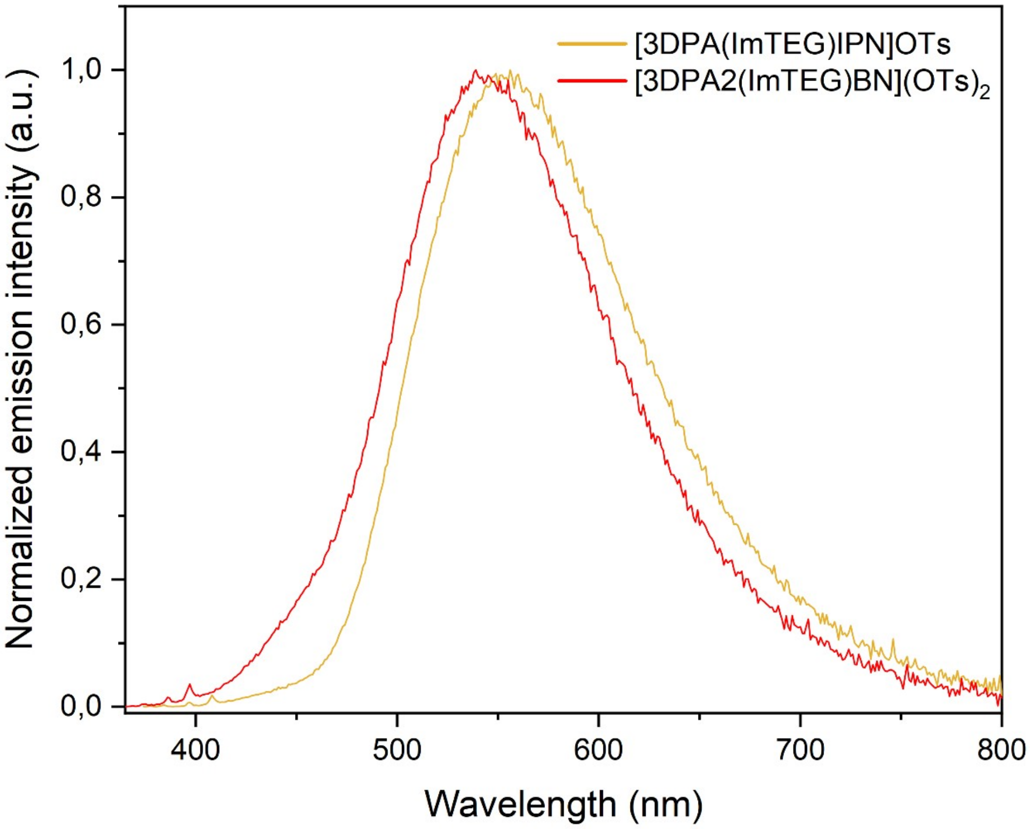

Figure S39. Normalized photoluminescence spectra of 5·10^{-6} M solutions of [3DPA(ImTEG)IPN]OTs (yellow line), and [3DPA2(ImTEG)Bn](OTs)$_2$ (red line) in non-degassed acetonitrile. λ_{exc} was set to 364 nm for [3DPA(ImTEG)IPN]OTs and to 355 nm for [3DPA2(ImTEG)BN](OTs)$_2$.

ECL signal stability of water-soluble compounds

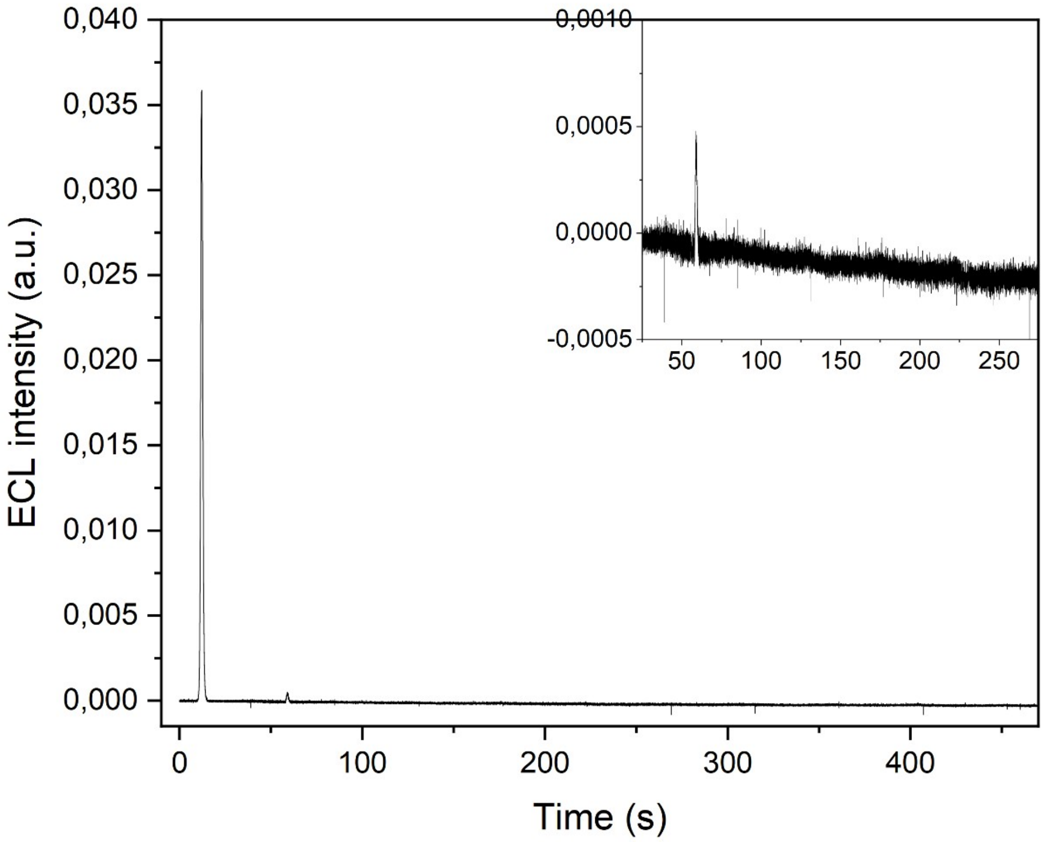

Figure S40. ECL intensity [3DPA(ImTEG)IPN]OTs (0.5 mM) in 0.3 M PB with 180 mM TPrA collected versus time. The ECL signal was recorded over 10 CV-ECL cycles at 100 mV/s in which the electrode potential was swept from the open circuit potential (OCP) to 1.75 V vs SCE, then back to -0.55 V and finally completing the last cycle by returning to OCP. PMT bias of 750 V; 000.0 µA amplification.

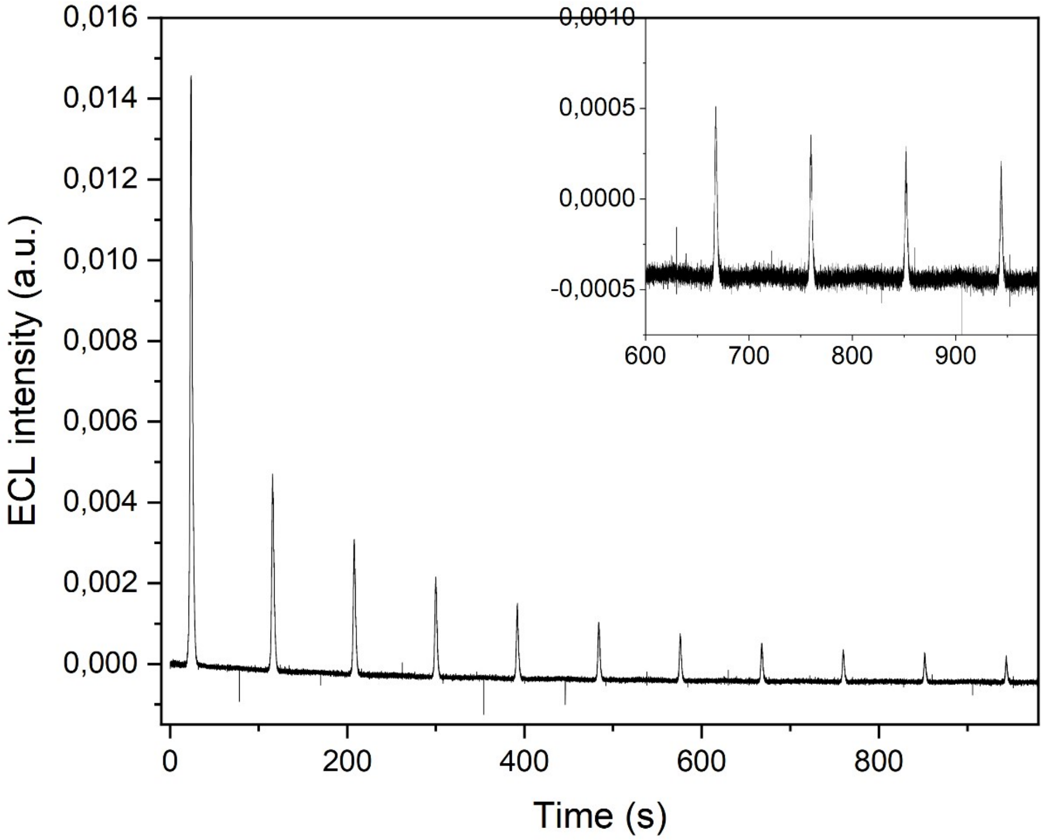

Figure S41. ECL intensity [3DPA2(ImTEG)BN](OTs)$_2$ (0.25 mM) in 0.3 M PB with 180 mM TPrA collected versus time. The ECL signal was recorded over 11 CV-ECL cycles at 50 mV/s in which the electrode potential was swept from the open circuit potential (OCP) to 1.75 V vs SCE, then back to -0.55 V and finally completing the last cycle by returning to OCP. PMT bias of 750 V; 000.0 µA amplification.

References

1 R. K. Harris, E. D. Becker, S. M. Cabral De Menezes, R. Goodfellow, P. Granger, *Pure Appl. Chem.,* 2001, ***73***, 1795–1818.

2 D. B. G. Williams, M. Lawton, *J. Org. Chem.,* 2010, ***75***, 8351–8354.

3 Liu, M. Nishiura, Y. Wang, Z. Hou, *J. Am. Chem. Soc.* **2006**, *128*, 5592–5593.

4 T. Fiala, K. Sleziakova, K. Marsalek, K. Salvadori, V. Sindelar, *J. Org. Chem.* **2018**, *83*, 1903–1912

5 J. C. Antilla and S. L. Buchwald, *Org. Lett.*, 2001, **3**, 2077–2079.

6 M. Garreau, F. Le Vaillant and J. Waser, *Angew. Chem. Int. Ed.*, 2019, **58**, 8182–8186.

7 Y. Nakamura, L. Ilies and E. Nakamura, *Org. Lett.*, 2011, **13**, 5998–6001.

8 E. Ishow, A. Brosseau, G. Clavier, K. Nakatani, R. B. Pansu, J.-J. Vachon, P. Tauc, D. Chauvat, C. R. Mendonça and E. Piovesan, *J. Am. Chem. Soc.*, 2007, **129**, 8970–8971.

8 J. A. Crosby, J. N. Demas, *J. Phys. Chem.*, 1971, **75**, 991-1024.

9 A. Paul, R. Borrelli, H. Bouyanfif, S. Gottis, F. Sauvage, *ACS Omega,* 2019, **4**, 14780–14789.

10 W. L. Wallace, A. J. Bard, *J. Phys. Chem.,* 1979, **83**, 1350–1357.

11 M. J. Frisch, G. W. Trucks, H. B. Schlegel, G. E. Scuseria, M. A. Robb, J. R. Cheeseman, G. Scalmani, V. Barone, G. A. Petersson, H. Nakatsuji, X. Li, M. Caricato, A. V. Marenich, J. Bloino, B. G. Janesko, R. Gomperts, B. Mennucci, H. P. Hratchian, J. V. Ortiz, A. F. Izmaylov, J. L. Sonnenberg, D. Williams-Young, F. Ding, F. Lipparini, F. Egidi, J. Goings, B. Peng, A. Petrone, T. Henderson, D. Ranasinghe, J. Zakrzewski, V. G.; Gao, N. Rega, G. Zheng, W. Liang, M. Hada, M. Ehara, K. Toyota, R. Fukuda, J. Hasegawa, M. Ishida, T. Nakajima, Y. Honda, O. Kitao, H. Nakai, T. Vreven, K. Throssell, J. Montgomery, J. A., J. E. Peralta, F. Ogliaro, M. J. Bearpark, J. J. Heyd, E. N. Brothers, K. N. Kudin, V. N. Staroverov, T. A. Keith, R. Kobayashi, J. Normand, K. Raghavachari, A. P. Rendell, J. C. Burant, S. S. Iyengar, J. Tomasi, M. Cossi, J. M. Millam, M. Klene, C. Adamo, R. Cammi, J. W. Ochterski, R. L. Martin, K. Morokuma, O. Farkas, J. B. Foresman, D. J. Fox, Revis. A.03, Gaussian, Inc., Wallingford CT, 2016.

12 J. Tomasi, B. Mennucci, R. Cammi, *Chem. Rev.*, 2005, **105**, 2999-3094

13 A. Lausi, M. Polentarutti, S. Onesti, J. R. Plaisier, E. Busetto, G. Bais, L. Barba, A. Cassetta, G. Campi, D. Lamba, A. Pifferi, S. C. Mande, D. D. Sarma, S. M. Sharma, G. Paolucci, *Eur. Phys. J. Plus*, 2015, **130**, 43

14 W. Kabscha, *Acta Crystallogr. D*, 2010, **66**, 125-132

15 G. M. Sheldrick*, Acta Crystallogr. A*, 2015, **71**, 3-8

16 G. M. Sheldrick*, Acta Crystallogr. C*, 2015, **71**, 3-8

17 P. Emsley, B. Lohkamp, W. G. Scott, K. Cowtan, *Acta Crystallogr. D*, 2010, **66**, 486-501

18 A. L. Spek, *Acta Crystallogr. C*, 2015, **71**, 9-18

19 L. J. Farrugia, *J. Appl. Crystallogr.*, 2012, **45**, 849-854
